# Mercury-induced epigenetic transgenerational inheritance of abnormal neurobehavior is correlated with sperm epimutations in zebrafish

**DOI:** 10.1371/journal.pone.0176155

**Published:** 2017-05-02

**Authors:** Michael J. Carvan, Thomas A. Kalluvila, Rebekah H. Klingler, Jeremy K. Larson, Matthew Pickens, Francisco X. Mora-Zamorano, Victoria P. Connaughton, Ingrid Sadler-Riggleman, Daniel Beck, Michael K. Skinner

**Affiliations:** 1 School of Freshwater Sciences, University of Wisconsin-Milwaukee, Milwaukee, Wisconsin, United States of America; 2 Zilber School of Public Health, University of Wisconsin-Milwaukee, Milwaukee, Wisconsin, United States of America; 3 Department of Biology, American University,Washington, DC, United States of America; 4 Center for Reproductive Biology, School of Biological Sciences, Washington State University, Pullman, WA, United States of America; University of Louisville School of Medicine, UNITED STATES

## Abstract

Methylmercury (MeHg) is a ubiquitous environmental neurotoxicant, with human exposures predominantly resulting from fish consumption. Developmental exposure of zebrafish to MeHg is known to alter their neurobehavior. The current study investigated the direct exposure and transgenerational effects of MeHg, at tissue doses similar to those detected in exposed human populations, on sperm epimutations *(i*.*e*., differential DNA methylation regions [DMRs]) and neurobehavior (*i*.*e*., visual startle and spontaneous locomotion) in zebrafish, an established human health model. F0 generation embryos were exposed to MeHg (0, 1, 3, 10, 30, and 100 nM) for 24 hours *ex vivo*. F0 generation control and MeHg-exposed lineages were reared to adults and bred to yield the F1 generation, which was subsequently bred to the F2 generation. Direct exposure (F0 generation) and transgenerational actions (F2 generation) were then evaluated. Hyperactivity and visual deficit were observed in the unexposed descendants (F2 generation) of the MeHg-exposed lineage compared to control. An increase in F2 generation sperm epimutations was observed relative to the F0 generation. Investigation of the DMRs in the F2 generation MeHg-exposed lineage sperm revealed associated genes in the neuroactive ligand-receptor interaction and actin-cytoskeleton pathways being effected, which correlate to the observed neurobehavioral phenotypes. Developmental MeHg-induced epigenetic transgenerational inheritance of abnormal neurobehavior is correlated with sperm epimutations in F2 generation adult zebrafish. Therefore, mercury can promote the epigenetic transgenerational inheritance of disease in zebrafish, which significantly impacts its environmental health considerations in all species including humans.

## Introduction

Heavy metals (*e*.*g*., lead [Pb], and mercury [Hg]) are among the top ten chemicals or groups of chemicals of global public health concern identified by the World Health Organization due to their known neurotoxicity [1]. Glacier ice core studies have revealed that the environmental release of such metals has greatly accelerated during the past century [2, 3]. Hg is released into the atmosphere via both natural and anthropogenic sources, with artisanal gold mining and coal combustion accounting for more than 50% of all anthropogenic inputs [4, 5]. Once Hg enters water or soil, anaerobic bacteria convert inorganic Hg to the more highly neurotoxic form, methylmercury (MeHg) [6]. MeHg enters the food web through the consumption of the Hg-converting bacteria by zooplankton [7], and is strongly biomagnified accumulating at high levels in large predatory fishes and other top predators including humans [8]. Previous observations have demonstrated dramatic impacts on phenotypes and health from Hg exposures in a variety of species, including humans [9, 10]. The zebrafish is a powerful model of human development and disease due its similar physiological, morphological, and genetic characteristics with humans [11]. This laboratory has previously demonstrated that direct developmental exposure of F0 generation zebrafish to MeHg leads to deficits in learning and memory, startle reflex responses, and retinal electrophysiology in adulthood [12, 13]. However, knowledge as to how direct developmental MeHg may impact future generations via epigenetics is severely lacking.

An appreciation that epigenetics is a complementary mechanism with genetics for the molecular control of biology has developed [14, 15]. Epigenetics being defined as “molecular factors and processes around DNA that regulate genome activity independent of DNA sequence and are mitotically stable” [15, 16]. The currently known epigenetic factors and processes include DNA methylation, histone modifications, selected non-coding RNA and chromatin structure. The ability of environmental factors to alter epigenetics while not changing DNA sequence provides a molecular mechanism for the impacts of environment on phenotypic variation, disease etiology and evolution [14, 17]. A large number of environmental factors from nutrition, stress and toxicants can developmentally alter a variety of phenotypes in species from plants to humans through epigenetic mechanisms [15]. Although direct environmental exposures on somatic cell development can influence the individual exposed, epigenetic changes in the germline (sperm or egg) are required to transmit epigenetic information to the next generation, and is termed epigenetic inheritance [15, 17]. In the event the epigenetic alterations become permanently programmed and transmitted to subsequent generations in the absence of direct exposure, this is defined as epigenetic transgenerational inheritance [15, 18]. Therefore, environmental factors can developmentally impact epigenetic programming to influence phenotypic variation of the individual exposed and if the germline is affected generational impacts develop. The potential transgenerational impacts of heavy metals, such as Hg, require further investigation. The current study is designed to investigate the direct exposure and, for the first time, the transgenerational effects of MeHg on sperm epimutations and phenotypes in the zebrafish.

The zebrafish model has emerged as alternative to rodent, the traditional laboratory model of human toxicology, in the study of toxicant-induced transgenerational inheritance of abnormal phenotypes. Studies have shown that similarities exist in the DNA methylation and demethylation mechanisms between zebrafish and mammals (*i*.*e*., human and mouse); except zebrafish lack parental imprinting [19]. Conversely, DNA methylation reprogramming during early development differ, but tissue distribution of methylated DNA is similar between zebrafish and mammals at later developmental stages. That said, zebrafish have been exploited to investigate the transgenerational effects of a few well-established toxicants (*e*.*g*., 2,3,7,8-tetracholordibenzo-*p*-dioxin [TCDD; [20–22]], bisphenol A [23], polyaromatic hydrocarbon mixtures [24], and benzo[a]pyrene [25]). For instance, Xu *et al*. [26] have shown that very high developmental MeHg exposure (100 nM) in zebrafish can promote transgenerational learning deficits assessed via an avoidance conditioning assay. However, these studies did not focus on elucidating the role of epigenetics in the inheritance of the abnormal phenotypes.

The objective of the current study was to investigate the direct exposure and transgenerational effects of MeHg on sperm epimutations and neurobehavioral phenotypes in the zebrafish. It was hypothesized that MeHg alters the epigenetic programming of the male zebrafish germline (*i*.*e*., sperm) to promote the epigenetic transgenerational inheritance of sperm epimutations and abnormal physiology, as evidenced via neurobehavioral endpoints. The molecular effects of MeHg on the germline (sperm) were also investigated in both the directly exposed F0 generation and transgenerational F2 generation. Genome-wide molecular analysis of differential DNA methylation regions (DMRs) was performed. Observations provide the first report of heavy metal-induced epigenetic transgenerational inheritance of abnormal neurobehavior.

## Methods

### Animal studies

All animal care and experiments, except for the retinal electrophysiology study, were pre-approved by the Institutional Animal Care and Use Committee, Environmental Protection, and Laboratory Safety offices of University Safety & Assurances at the University of Wisconsin-Milwaukee (UWM). All the protocols and procedures associated with the retinal electrophysiology study were pre-approved by the Institutional Animal Care and Use Committee at American University (Washington, DC). All experimental procedures comply with the *Guidelines for Use of Zebrafish in the NIH Intramural Research Program (rev*. *6/22/16)*. All animal experiments were performed at least twice with three replicate groups within each exposure. For specific analyses, every effort was made to ensure that each replicate was equally represented.

### Methylmercury exposure and breeding scheme

**Fig 1:**
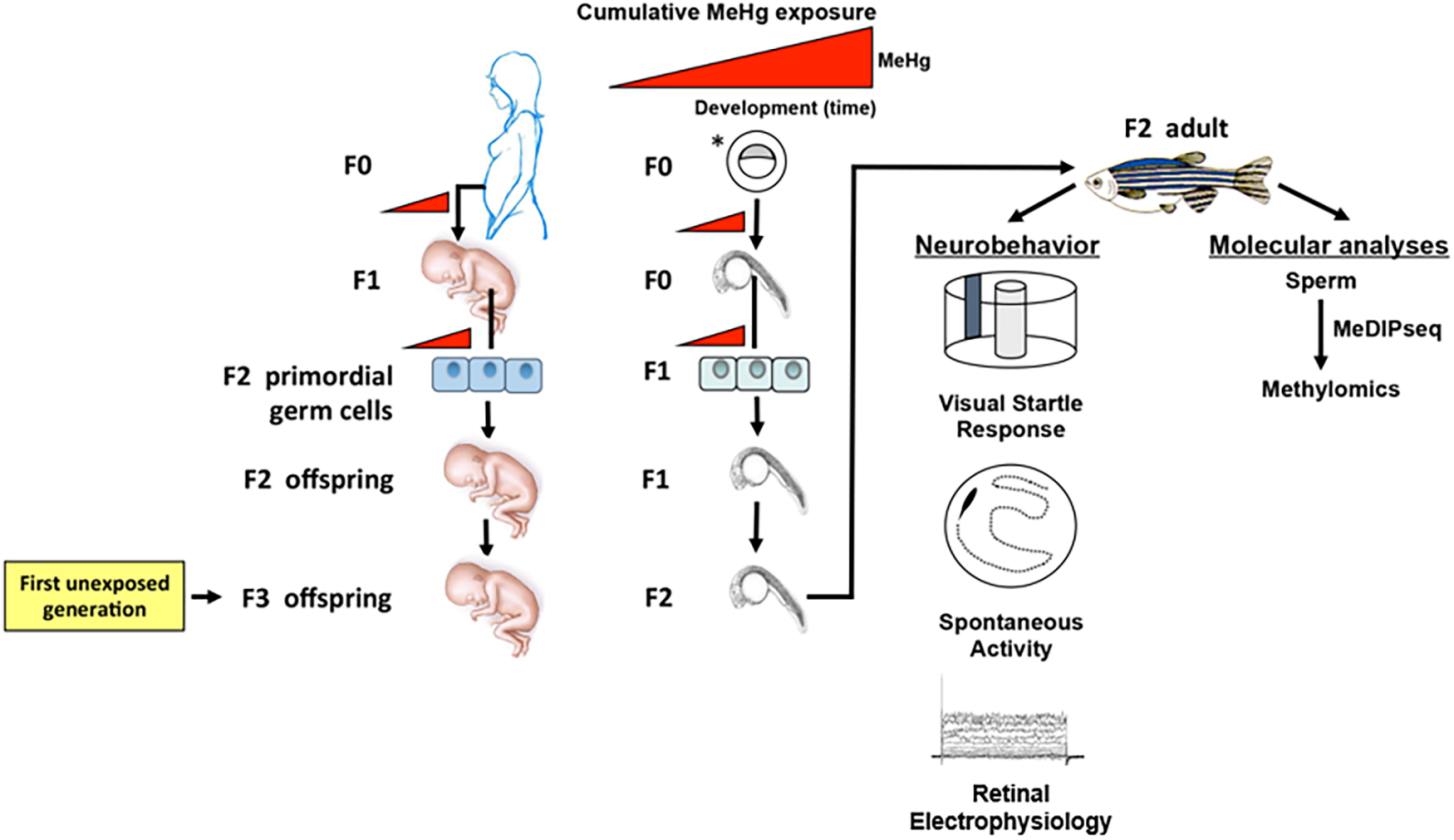
Schematic of the experimental design presented in comparison to a human exposure scenario. Breeding/exposure scheme and summary of analyses are presented. *In our paradigm, F0 generation zebrafish embryos are developmentally exposed to MeHg *ex vivo* with the intention of emulating a human maternal exposure.

In our zebrafish model, the directly exposed embryos are analogous to the F1 generation in mammalian *in utero* exposure models (Fig 1). The outbred EK strain we use was originally obtained from Ekkwill Waterlife Resources, Gibsonton, FL, and has been maintained as an outbred population in the laboratory for more than 15 generations. EkkWill has maintained its large population to supply fish to the pet store trade since 1962. For this study, large populations of greater than 200 adult zebrafish were bred *en masse* by natural breedings to create the F0, F1, and F2 generations.

Adult EK strain zebrafish were housed at a maximum density of 10 fish/L in a flow-through dechlorinated water system maintained at 26–29°C on a 14:10 hour light: dark photoperiod at the Aquatic Animal Facility of the Children’s Environmental Health Sciences Core Center at the UWM. Eggs were collected ≤1-hour post fertilization (hpf) and placed into metal-free, plastic culture dishes (100 mm diameter × 15 mm depth) in E2 medium (pH 7.2; 1 L of E2 medium contained: 0.875 g NaCl, 0.038 g KCl, 0.120 g MgSO_4_, 0.021 g KH2PO_4_, and 0.006 g Na_2_HPO_4_). The newly fertilized eggs were then transferred to two 12-well (non-treated) culture plates (10 eggs/well) and rinsed twice with E2 medium. The fertilized eggs were exposed to 1.5 ml/well of E2 medium containing MeHg (0, 1, 3, 10, 30, and 100 nM nominal concentration as methylmercury chloride—CH_3_ClHg, with ethanol as the vehicle [< 0.01% total volume]) until 24 hpf. The laboratory has previously demonstrated that direct MeHg exposure in F0 generation embryos impairs the visual startle response and other neurobehavioral phenotypes as adults [12]. The current experimental design involved exposures of F0 generation fertilized embryos to MeHg (0, 1, 3, 10, 30, and 100 nM nominal concentration) based on our previous study [12]. Martin *et al*. [27] have demonstrated that exposure of zebrafish (n = 150) to 5-azacytidine, a well-known inhibitor of DNA methyltransferases (Dnmts), from ≤1 to 24 hpf yielded a greater incidence of abnormal development at 24 hpf compared to exposure periods of 3 to 24 hpf or 6 to 24 hpf. These results suggest that zebrafish embryos are most sensitive to possible DNA methylation perturbations in the 1 to 4 hpf window. Thus, the developmental MeHg exposure period of <4 to 24 hours reported in the laboratory’s previous neurobehavioral toxicity study [12] was extended to <1 to 24 hpf in the current study.

At 24 hpf, the embryos were rinsed three times with E2 medium and transferred to 2-L tanks (60 embryos per tank) containing static E2 medium for rearing. After the initial 24-hr MeHg exposure of the F0 generation embryos, there was no additional exposure to Hg during the entire life cycle of the zebrafish beyond the very low concentration inherent to all lab animal foods. Starting at 5 days post fertilization (dpf), larvae were fed 5–100 micron Golden Pearl Reef & Larval Fish Diet (Brine Shrimp Direct, Ogden, UT). Platinum grade *Artemia* nauplii (Argent Laboratories, Redmond, WA) were fed to the larvae starting at 9 dpf. At 21 dpf, the fish were transferred to 1-L flow-through tanks and reared using standard husbandry techniques until 4 months of age. At this point, the zebrafish were ready for subsequent breeding and behavioral assays (4–8 months of age). Juvenile and adult fish were fed a combination of platinum and gold grade *Artemia* nauplii and Aquarian™ flake food (Aquarium Pharmaceuticals Inc., Chalfont, PA). The health and development of F0, F1, and F2 generation zebrafish was monitored daily throughout the course of the study. Fish that exhibited developmental abnormalities *(e*.*g*., lack of air bladder inflation and/or malformation of trunk, tail, or fins) were excluded as larvae—less than 1% of all individuals and was comparable in all exposure groups. All adult zebrafish were confirmed as morphologically normal prior to experimentation.

After each spawning, all embryos from a given lineage and generation were pooled. The F0 generation control or MeHg lineage was reared to adults and bred as a population to generate the F1 generation lineages, which was then bred as a population to generate the F2 generation with specific control and MeHg exposure lineages. The F1 and F2 generations were never exposed to exogenous MeHg, and all F2 generation lineages had Hg levels that were not significantly different from background *(i*.*e*., F2 generation control; S1 Table). The F1 generation is also considered to be directly exposed as primordial germ cells within the F0 generation embryos at the time of initial MeHg exposure. F2 generation adult fish (not directly exposed to MeHg) were used for phenotypic analyses (*i*.*e*., neurobehavior and retinal electrophysiology) and sperm was collected for molecular studies (Fig 1).

### Determination of total mercury in zebrafish embryos and exposure media

Zebrafish embryos were exposed to MeHg until 24 hpf as described above. Thereafter, the embryos were reared until chorion loss and yolk resorption (144 hpf) in clean E2 medium. MeHg accumulates rapidly in the chorion and yolk, and at that point is not directly influencing the embryo. Elimination of the chorion and yolk from the analysis ensures the accurate evaluation of total Hg accumulation within the embryo itself. At 144 hpf, embryos were collected, counted into individual samples of 200, and anesthetized on ice. Each embryo sample was then pipetted onto a circular piece (diameter = ~2.4 cm) of 200-μm nylon mesh, which then was placed briefly on a Kim-wipe® to remove excess water. All samples remained on its nylon mesh and were then weighed (wet weight). Next, each sample was carefully washed off the nylon mesh with Hg-free ultrapure water into a 1.75-ml microcentrifuge tube. Each sample had excess water removed, was freeze dried for 20 hours, and then stored at -80°C until microwave digestion.

For sample digestion, 1 ml of a concentrated nitric/sulfuric acid mixture (7 parts HNO_3_: 3 parts H_2_SO_4_) was added to each freeze-dried sample. Each acid/embryo suspension was transferred to a 3-ml Teflon® MicroVessel (CEM Corporation, Matthews, NC), incubated at room temperature for 30 minutes, and digested using a MARS 6 microwave reaction system (CEM Corporation; ramp time = 10.5 minutes, hold time = 15 minutes, temperature = 130°C) per the manufacturer’s instructions. Each 1 ml of digestant was then diluted 1:4.5. Total mercury analysis was conducted on 100-μl samples of each diluted digestant as well as both pre- and post-exposure E2 media (digestion step not required) using a MERX-T automated mercury system (Brooks Rand Instruments, Seattle, WA) with Mercury Guru^TM^ software (version 4.6.7) via the manufacturer’s protocol in accordance with *EPA Method 1631*. Three pools of 200 embryos from each exposure group (six) were generated in triplicate experiments and then analyzed for Hg content.

### Visual startle response assay

To prevent experimental bias, adult (4–6 months old, 1.5–2.0 cm standard length) male (n = 10) and female (n = 10) zebrafish from all experimental groups (120 adult zebrafish in total) were assigned random numbers to blind the observer to their F2 generation lineage. Thereafter, the behavioral response of the fish to visual stimuli was evaluated as previously described [12]. All neurobehavioral assays (visual startle response and spontaneous locomotion assays) were conducted from 12:00pm-4:00 pm to limit the effects of circadian rhythms [28]. Blinded analysis of the number of C-start escape reactions exhibited by fish during the encounter with the rotating black bar was quantified.

### Spontaneous locomotion assay

Each adult zebrafish (n = 15–20 per exposure group) assessed for the visual startle response was also evaluated for spontaneous locomotion to: (a) quantify spontaneous swimming ability and; (b) ensure that any differences in the measured visual response were not caused by swimming defects. Individual fish (exposure group blinded to the observer) were placed in a clean glass dish (10 cm diameter and 5 cm depth) in an enclosed chamber. The light intensity inside the chamber was produced by a standard computer monitor (76 lux). The chamber was equipped with four c920 USB (Logitech International S.A., Newark, CA) cameras for capturing digital video of four arenas simultaneously. Refer to the studies of Mora-Zamorano *et al*. [29, 30] for a diagram and further description of the behavioral observation chamber. The fish were acclimated for 5 minutes and then the spontaneous locomotion (*i*.*e*., unstimulated movement) of each fish was recorded for an additional 5 minutes. The digital videos were obtained in M-MJPEG format using the Matlab Image Acquisition Toolbox (MathWorks, Natick, MA) at a 960 x 720 resolution. Each video was cropped to 600 x 600 using FFmeg software (https://www.ffmpeg.org/) prior to being imported into EthoVision XT (Noldus Information Technology Inc, Leesburg, VA) for the automated analysis of the distance each fish traveled within a 5-minute period.

### Retinal electrophysiology

Live adult zebrafish from the same F2 generation lineages that were used in the neurobehavioral assays were shipped to the laboratory of Dr. Victoria Connaughton at American University (Washington, DC) for the conduction of retina electrophysiology experiments. Upon arrival, the adult zebrafish were placed in holding/quarantine aquaria (28–29°C; 14 hour light: 10 hour dark photoperiod) for at least a week. While in quarantine, the fish were monitored and fed TetraMin tropical flakes (Tetra, Melle, Germany) daily. This allowed them to acclimate and recover from shipping as well as make sure they were in good health. After the quarantine period, the fish were transferred to a main aquaria system (28–29°C; 14 hour light: 10 hour dark photoperiod) and remained there for a minimum of several weeks prior to experimentation. All fish were fed a combination diet that included TetraMin tropical flakes and live brine shrimp at a maximum of twice per day. Prior to experimentation, fish (at ~7–9 months of age) were anesthetized in a 0.02% buffered tricaine solution until gill movements had ceased. Fish were then removed from the tricaine, decapitated, and the eyes were removed. Retinal slices were prepared following established protocols [31–33]. Whole-cell voltage-gated current responses were then recorded from bipolar cells within intact zebrafish retinal slice preparations using methods described previously [12]. Recordings were made using an Axopatch 1-D patch clamp amplifier (Molecular Devices LLC, Sunnyvale, CA) and Axon™ pCLAMP® electrophysiology data acquisition & analysis software (version 8.0). The recordings were also analyzed using pCLAMP® software. All experiments for a given exposure were done in the same week.

### Sperm collection

F0 and F2 generation zebrafish sperm from the 30 nm MeHg exposure lineage were chosen for epigenetic analysis because: (a) visual deficit was observed in adult F0 generation zebrafish developmentally exposed to 30-nm MeHg in the laboratory’s previous [12] and current study and; (b) the current dosimetry results revealed a tissue dose of ~ 800 ppb THg in the F0 generation zebrafish exposed to 30 nM MeHg. A tissue dose of ~800 ppb THg is near the estimated mean measured in umbilical cord blood samples collected from developmentally exposed human populations in the Minamata poisoning event (maximum reported cord blood concentration of ~1.5 ppm) [34–36]. Fish were anesthetized for sperm collection by using 0.004% buffered tricaine. Abdominal massage followed by microcapillary suction was used to collect sperm. The mean volume of sperm collected from each adult zebrafish was estimated to be ~5.0 μL. The volume was variable as was the sperm density as observed by consistency/color of semen. Sperm samples (n = 12, 6 samples per exposure group) were immediately frozen in liquid nitrogen and stored at -80°C until shipped to the Skinner laboratory.

### DNA preparation

Upon arrival at the Skinner laboratory, the frozen zebrafish sperm samples were stored at -20°C. Genomic DNA from thawed sperm was prepared as follows: One hundred μl of sperm suspension was used then 820 μl DNA extraction buffer (50 mM Tris pH 8, 10 mM EDTA pH 8, 0.5% SDS) and 80 μl 0.1 M Dithiothreitol (DTT) added and the sample incubated at 65°C for 15 minutes. Proteinase K (80 μl, 20 mg/ml) was added and the sample incubated on a rotator at 55°C for 2 hours. After incubation, 300 μl of protein precipitation solution (Promega, A795A, Madison, WI) was added, the sample mixed and incubated on ice for 15 minutes, then spun at 4°C at 16,000 x g for 20 minutes. The supernatant was transferred to a fresh tube, then precipitated over night with the same volume 100% isopropanol and 2 μl glycoblue at -20°C. The sample was then centrifuged and the pellet washed with 75% ethanol, then air-dried and resuspended in 100 μl H_2_O. DNA concentration was measured using the Nanodrop (Thermo Fisher, Waltham, MA).

### Methylated DNA immunoprecipitation MeDIP

Methylated DNA Immunoprecipitation (MeDIP) with genomic DNA was performed as follows: three zebrafish sperm DNA pools were generated from each exposure group (control and MeHg). Each pool contained genomic DNA from two individuals, and each individual contributed 3 μg of genomic DNA. Each pool contained 2 individuals for a total of n = 6 for biological (animal) variation and n = 3 for technical variation per exposure group. The resulting 6 μg of genomic DNA per pool was diluted to 150 μl with 1x Tris-EDTA (TE, 10 mM Tris, 1 mM EDTA) and sonicated with a probe sonicator using 5 x 20 pulses at 20% amplitude. Fragment size (200–800 bp) was verified on a 1.5% agarose gel. Sonicated DNA was diluted to 400 μl with 1x TE and heated to 95°C for 10 minutes, then incubated in ice water for 10 minutes. Then, 100 μl of 5x immunoprecipitation (IP) buffer (50 mM Sodium Phosphate pH 7, 700 mM NaCl, 0.25% Triton X-100) and 5 μg of 5-mC monoclonal antibody (Diagenode, Denville, NJ, C15200006-500) were added and the sample was incubated on a rotator at 4°C overnight. The next day, Protein A/G Agarose Beads from Santa Cruz were prewashed with 1x PBS/0.1% BSA and resuspended in 1x IP buffer. Eighty μl of the bead slurry were added to each sample and incubated at 4°C for 2 hours on a rotator. The bead-DNA-antibody complex was washed 3 times with 1x IP buffer by centrifuging at 6,000 rpm for 2 minutes and resuspending in 1x IP buffer. After the last wash, the bead-complex was resuspended in 250 μl of digestion buffer (50 mM Tris pH 8, 10 mM EDTA pH 8, 0.5% SDS) with 3.5 μl Proteinase K (20mg/ml) per sample and incubated on a rotator at 55°C for 2 hours. After incubation, DNA was extracted with the same volume of Phenol-Chloroform-Isoamyalcohol and then with the same volume chloroform. To the supernatant from chloroform extraction, 2 μl glycoblue, 20 μl 5M sodium chloride and 500 μl 100% cold ethanol were added. DNA was precipitated at -20°C overnight, then spun for 20 minutes at 13,000 rpm at 4°C, washed with 75% ethanol and air-dried. Dry pellet was resuspended in 20 μl H_2_O and concentration measured in Qubit using the Qubit ssDNA Assay Kit (Life Technologies, Carlsbad, CA).

### MeDIP-Seq analysis

The MeDIP pools were used to create libraries for next generation sequencing (NGS) at the University of Reno, NV, Genomics Core Laboratory using the NEBNext® Ultra™ RNA Library Prep Kit for Illumina® (San Diego, CA) starting at step 1.4 of the manufacturer’s protocol to generate double stranded DNA. After this step, the manufacturer’s protocol was followed. Each pool received a separate index primer. NGS was performed at that same laboratory using the Illumina HiSeq 2500 with a PE50 application, with a read size of approximately 50 bp and approximately 100 million reads per pool. Two libraries each were run in one lane comparing one control with one MeHg exposed pool in each lane.

### Statistics and bioinformatics

Power analyses based on previously published data [12] and the data collected from the visual startle response and spontaneous locomotion assays of the F0 generation MeHg-exposed and control lineages in the current study revealed that a minimum sample size of 15 animals was sufficient to obtain 95% power, at a p-value of 0.05, in the neurobehavioral assays. Normality and equal variance of each neurobehavioral data set was assessed using SigmaPlot 12.0 software (Systat software Inc, San Jose, CA) prior to further statistical evaluation between experimental groups. Data collected from the spontaneous locomotion assays were confirmed to be normal and of equal variance, thus justifying our use of the parametric one-way analysis of variance (ANOVA, SigmaPlot 12.0). Data from the visual startle response assays was determined to be non-normal, but of equal variance, thus supporting our use of the non-parametric ANOVA on Ranks (SigmaPlot 12.0) to analyze these results. Chi-square analysis (SigmaPlot 12.0) was conducted to evaluate the expected versus observed inheritance of the neurobehavioral phenotypes. After confirmation of data normality and equal variance, one-way ANOVA was also used to assess the concentration of total Hg in MeHg F2 generation lineages compared to control. Based on preliminary Hg analysis data and our previous work [12], a sample size of 3 was chosen for the evaluation of low variance endpoints (nominal exposure concentrations and F2 generation lineages), and a sample size of 9 was deemed appropriate for those with greater variance (F0 generation lineages). If a one-way ANOVA revealed statistical significance, either the Holm-Sidak (spontaneous locomotion assay) or Tukey (retinal electrophysiology) method was conducted for multiple comparison *post-hoc* analyses. Multiple comparison *post-hoc* analysis of statistically significant non-parametric data (ANOVA on Ranks) was performed via Dunn’s Method.

Statistical analyses for the bipolar cell electrophysiology data were conducted using SPSS 20.0 for Windows software (IBM Corp., Armonk, NY) and one-way ANOVA (SigmaPlot 12.5). For the retinal electrophysiology recordings, all results were included in data analyses (*i*.*e*., no outliers). Samples sizes were based on previous research [12]. The sample size refers to the number of cells examined, with each recording being treated as a separate response or replicate. For each cell, two recordings were taken, with the 2nd (presumed more stable) one use for data analysis. This approach was taken for all the fish (10 fish per exposure group, 2 replicates of each for a total of 20 fish per group) collected for these experiments. One bipolar cell (I_A_ current, control) was omitted from analysis because a full response was not obtainable. Furthermore, the samples were blinded for the electrophysiology experiments. Recordings from two fish were collected per day until the completion of all exposure groups. For example, if ten fish were present in a given exposure group, these fish were included in experiments over the course of 5 consecutive days before progressing to the next exposure group. However, which exposure group was used, in which order, and which fish were selected from each group was random. A p-value <0.05 was considered statistically significant in the analyses of all phenotype data.

In regards to bioinformatics, the basic read quality was verified using summaries produced by the FastQC program. The reads for each sample for DMR analyses were mapped to the GRChz10 zebrafish genome using Bowtie2 [36] with default parameter options. The mapped read files were then converted to sorted BAM files using SAMtools [37, 38]. To identify DMRs, the reference genome was broken into 100 bp windows. The MEDIPS R package [39] was used to calculate differential coverage between control and exposure sample groups. The edgeR p-value [40] was used to determine the relative difference between the two groups for each genomic window. Windows with an edgeR p-value less than 10^−7^ were considered DMRs. The DMR edges were extended until no genomic window with an edgeR p-value less than 0.1 remained within 1000 bp of the DMR. CpG density and other information was then calculated for the DMR based on the reference genome. The DMRs that included at least two windows with an edgeR p-value <10^−7^ were then selected for further analysis and annotated.

DMRs were annotated using the biomaRt R package [41] to access the Ensembl database [42]. The genes that overlapped with DMR were then input into the KEGG pathway search [43, 44] to identify associated pathways. The DMR associated genes were manually then sorted into functional groups by consulting information provided by the DAVID [45], Panther [46], and Uniprot databases incorporated into an internal curated database (www.skinner.wsu.edu under genomic data). All molecular data has been deposited into the public database at NCBI (GEO # GSE89144).

## Results

### Neurobehavioral phenotypes

The mean tissue dose of total Hg in the MeHg-exposed F0 generation lineages ranged from ~ 20 ppb to ~3000 ppb; the unexposed (control) lineage yielded a mean tissue dose of ~5 ppb (S1 Table). Visual startle response and spontaneous locomotion assays were performed on each zebrafish to assess potential effects of MeHg on vision and swimming ability within our paradigm. Importantly, these assays also served to confirm that spontaneous locomotion problems were not influencing performance in the visual assay. An individual fish was considered to have a visual deficit or be hyperactive if its assay performance was either less than the 5^th^ percentile or greater than the 95^th^ percentile, of the control group values, respectively. Based on these criteria, individual fish were identified as being *normal* or *abnormal* with respect to each neurobehavior. Any individual fish that moved less than 25% of the total recording time was eliminated as an outlier (more than four standard deviations below the mean percentage of the time moved). A maximum of 2 fish were eliminated as outliers from any exposure lineage. In visual startle assays, one fish was eliminated from our analyses because the total time captured was less than four minutes.

**Fig 2:**
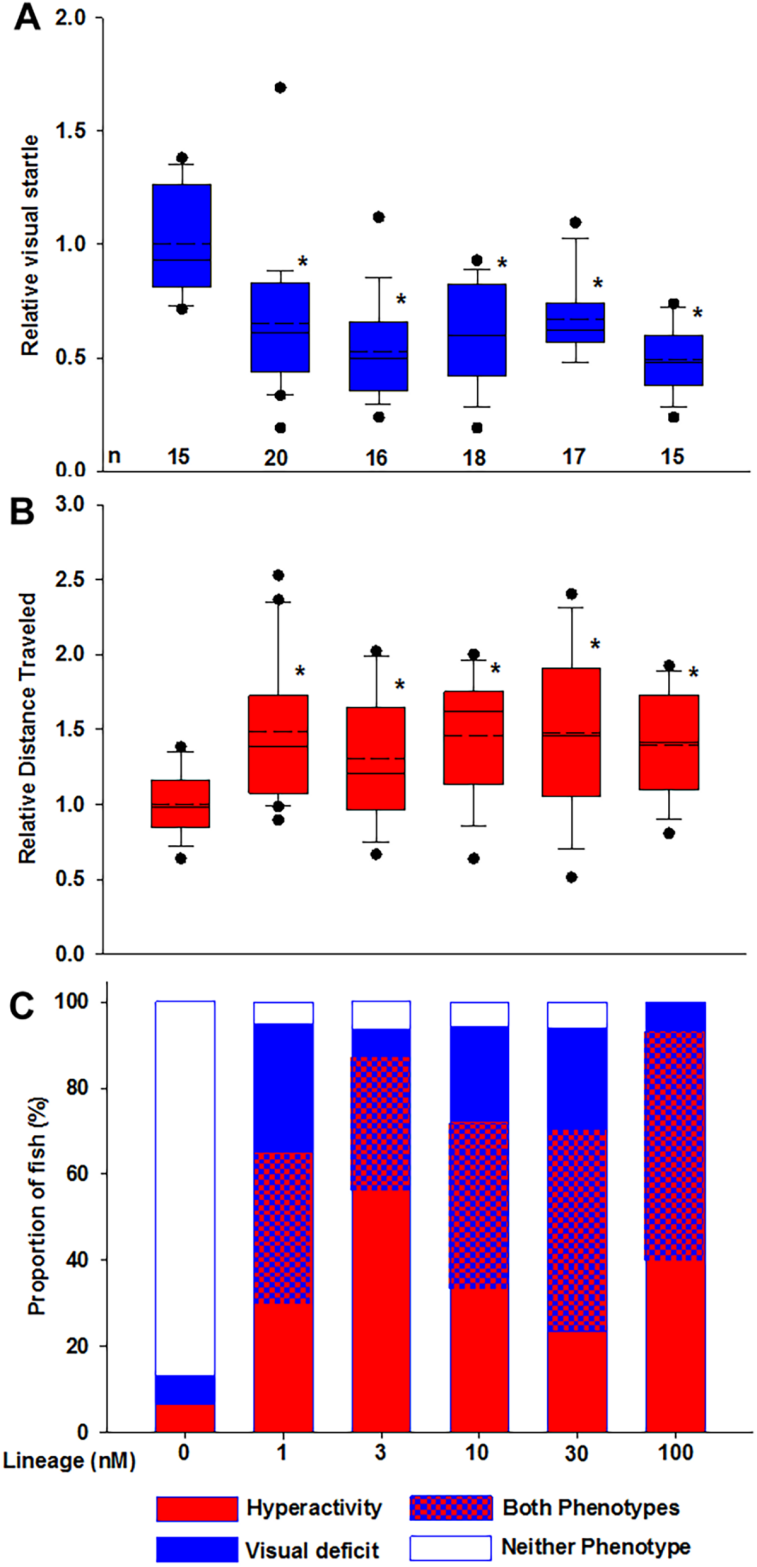
Transgenerational phenotypes. (A) Visual deficit and (B) hyperactivity observed in F2 generation zebrafish due to ancestral MeHg exposure (*p<0.001). (A) Analysis of variance (ANOVA) on Ranks and post-hoc analysis via Dunn’s Method revealed a statistically significant decrease in the visual startle response of the MeHg lineages compared to control (df = 5, H = 34.596, p<0.001). (B) One-way ANOVA and post-hoc Holm-Sidak analyses showed a statistically significant increase in the spontaneous locomotion in all MeHg lineages compared to control (df = 102, F = 3.498, p = 0.006). n = number of individual adult zebrafish. Solid horizontal lines represent the median, dashed horizontal lines represent the mean, the box represents the 25th and 75th percentiles, the whiskers show the 5th and 95th percentiles and outliers are represented by dots. (C) Proportion of the F2 generation lineages showing transgenerational phenotypes.

The visual startle and spontaneous locomotion assays were performed three times with similar results. During the final replication, each animal was subjected to both assays to explore the possibility that the phenotypes were dependent upon one another and possibly attributed to a problem at the same locus. The data from the final experiment are shown in Fig 2. Our results revealed visual deficits (Fig 2A) and hyperactivity (Fig 2B) in all MeHg F2 generation lineages compared to control. Therefore, a transgenerational effect of MeHg on the spontaneous locomotion and visual response of F2 generation fish was observed. A high percentage of animals had effects in both spontaneous locomotion and visual startle response and the highest exposure lineage showed phenotypic alteration in all animals (Fig 2C). As the ancestral MeHg exposure increased from 3–100 nM, the proportion of fish that exhibited both phenotypes also increased (Fig 2C, S2 Table). Intriguingly, the phenotypes appear to be inherited independently as the observed proportion of fish exhibiting both phenotypes are not significantly different from the expected value based on the proportion of each individual neurobehavioral abnormality (S2 Table).

### Retinal electrophysiology

Previously [12], the laboratory has shown that the outward potassium currents (I_K_ and I_A_) in adult zebrafish retinal bipolar cells are altered by developmental MeHg exposure. Here, whole-cell voltage-gated currents were measured in retinal bipolar cells from F2 generation zebrafish ancestrally exposed to MeHg. Most bipolar cells examined (n = 47) expressed I_K_; while the remainder (n = 27) expressed I_A_. For both currents, there was a clear trend of increasing current amplitude with increasing MeHg (Table 1, S1 Fig). Mean I_K peak_ current amplitudes elicited from a voltage step to +60 mV (V_hold_ = -60 mV) were significantly different (ANOVA, df = 34, F = 3.601, p = 0.039; S1 Fig) among control, 10 nM, and 30 nM MeHg lineages and a Tukey *post-hoc* test revealed the difference to be between the 30 nM and control lineages only.

**Table 1:** Amplitude changes in response to ancestral MeHg exposure of the Ik and IA currents.

**Ik**	**MeHg lineage**	**l**_**K**_ **peak amplitude in response to +60mV step**	**Percent change in peak amplitude from control**
	Control	382 ± 51.6pA (16)	—
	10 nM MeHg	584 ± 102.8pA (12)	53% increase
	30 nM MeHg	914 ± 313.9pA (7)	139% increase
	100 nM MeHg	561 ± 141.8pA (12)	47% increase
**IA**	**MeHg lineage**	**I**_**A**_ **peak amplitude in response to +60mV step**	**Percent change in peak amplitude from control**
	Control	556 ± 83.1pA (12)	—
	10 nM MeHg	734 ± 53pA (4)	32% increase
	30 nM MeHg	936 ± 156.5pA (6)	68% increase
	100 nM MeHg	597 ± 123.5pA (5)	7% increase

Mean (± SEM) peak amplitudes and the corresponding percent change in amplitude of the delayed rectifying I_K_ current, and the depolarization elicited I_A_ current, recorded from retinal bipolar cells in F2 generation zebrafish. Currents were elicited in response to a voltage step to +60mV. A significant trend (df = 34, F = 7.175, p = 0.012) of increased I_K_ current amplitude with increased MeHg was observed: current amplitude increased 53% in the 10 nM lineage and 139% in the 30 nM lineage. Peak I_K_ currents recorded from cells within the 100 nM exposure group were reduced and comparable to amplitudes recorded from cells in the 10 nM lineage (S1 Fig). Including the 100 nM lineage in the statistical analysis caused the differences in peak current amplitude to become non-significant (ANOVA, df = 46, F = 2.256, p = 0.095). The range of recorded peak current amplitudes was large. Variability was smallest in control animals (139–764 pA) and the greatest variability was observed in the 30 nM lineage, which was also the exposure that showed the greatest increase in current amplitude. A significant trend of increased I_A_ current amplitude was observed with increasing MeHg (df = 21, F = 6.256, p = 0.022) when control, 10 nM, and 30 nM lineages were compared (S1 Fig). However, mean peak current amplitudes were not significantly different (ANOVA, df = 21, F = 3.175, p = 0.065), even after inclusion of the 100 nM lineage (ANOVA, df = 26, F = 2.32, p = 0.102). Current amplitude increased 32% in the 10 nM lineage and 68% in the 30 nM lineage. As noted for I_K_, I_A_ peak current amplitude varied among cells; the smallest variability was seen in the 10 nM lineages (range = 628–878 pA) while variability within control, 30 nM, and 100 nM lineages were comparable. Sample sizes (n = number of individually recorded retinal bipolar cells) are given in parentheses. Control recordings include both water- and ethanol-treated fish are shown.

### Sperm epigenetic analysis

The differential DMRs between the control and 30 nM MeHg F0 and F2 generation lineages were identified in the sperm. The DNA from five different males were collected and pooled. Three different pools with different fish created n = 6 for biological (animal) variation and n = 3 for technical variation for both the control and MeHg lineages. Equal amounts of DNA from each individual was used in the pools and then the DNA was fragmented via sonication and the methylated DNA was immunoprecipitated (MeDIP) with an antibody to methylcytosine as previously described [47] in the Methods. The MeDIP DNA was used to generate libraries with specific bar codes and then analyzed with next generation sequencing (Seq) for an MeDIP-Seq analysis to identify the DMR, see Methods. The different pools of the control and MeHg lineages were paired for individual comparisons so three different experiments were performed and different sets of animals compared. The sperm from the F0 and F2 generations were analyzed separately.

A comparison of the control and MeHg lineages using MeDIP-Seq identified differential DMRs (Table 2). The analysis of several different statistically significant p-values is shown for single sites (100 bp windows) and for multiple sites (≥2 100 bp windows) in the same DMRs for both the F0 generation sperm and for the F2 generation comparisons (Table 2). As the statistical significance increases (p-value) the number of identified DMRs decreases as expected and the p<10^−7^ significance was selected for subsequent use and data presentation. The multiple site DMRs were the focus of the further analysis as this reduces the issue of false positives in the single site DMRs and provides a set of DMRs that are reproducible between all the experiments and presumably all animals. Although the data analyses focused on the most stringently selected DMRs, the other single site DMRs are also important as well. The DMR numbers for the sperm are presented in Table 2 and the most predominant number of adjacent (*i*.*e*., multiple) sites within a DMR is 2 (100 bp each), with the highest number of sites being 41 for the F0 generation DMRs and 27 for the F2 generation DMRs (Table 2). Therefore, the more stringently identified DMRs (multiple sites with p<10^−7^) provide a highly reproducible set (*i*.*e*., signature) that is presumed present in all animals, but the majority of DMRs were more variable among the populations. A list of DMRs for the sperm are presented in S3 Table for the F0 generation and S4 Table for the F2 generation.

**Table 2:** DMR numbers and statistics.

**Number of F0 generation DMRs**													
p-value	All Windows						Multiple Windows						
0.001	10125						2966						
1.00E-04	3005						1171						
1.00E-05	1383						634						
1.00E-06	811						413						
1.00E-07	533						291						
Number of significant windows		1	2	3	4	5		6	7	8	9	10	>10
Number of DMRs		242	94	52	33	27		16	16	13	9	5	26
**Number of F2 generation DMRs**													
p-value	All Windows						Multiple Windows						
0.001	22877						8370						
1.00E-04	8499						3429						
1.00E-05	4093						1771						
1.00E-06	2307						985						
1.00E-07	1414						617						
Number of significant windows		1	2	3	4	5		6	7	8	9	10	>10
Number of DMRs		797	278	148	80	45		19	7	10	8	4	18

The number of DMRs versus the p-value are shown for single site DMRs and multiple site DMRs for the zebrafish F0 generation sperm and for the zebrafish F2 generation sperm. The number of DMR and significant sites/windows at p<10^−7^

**Fig 3:**
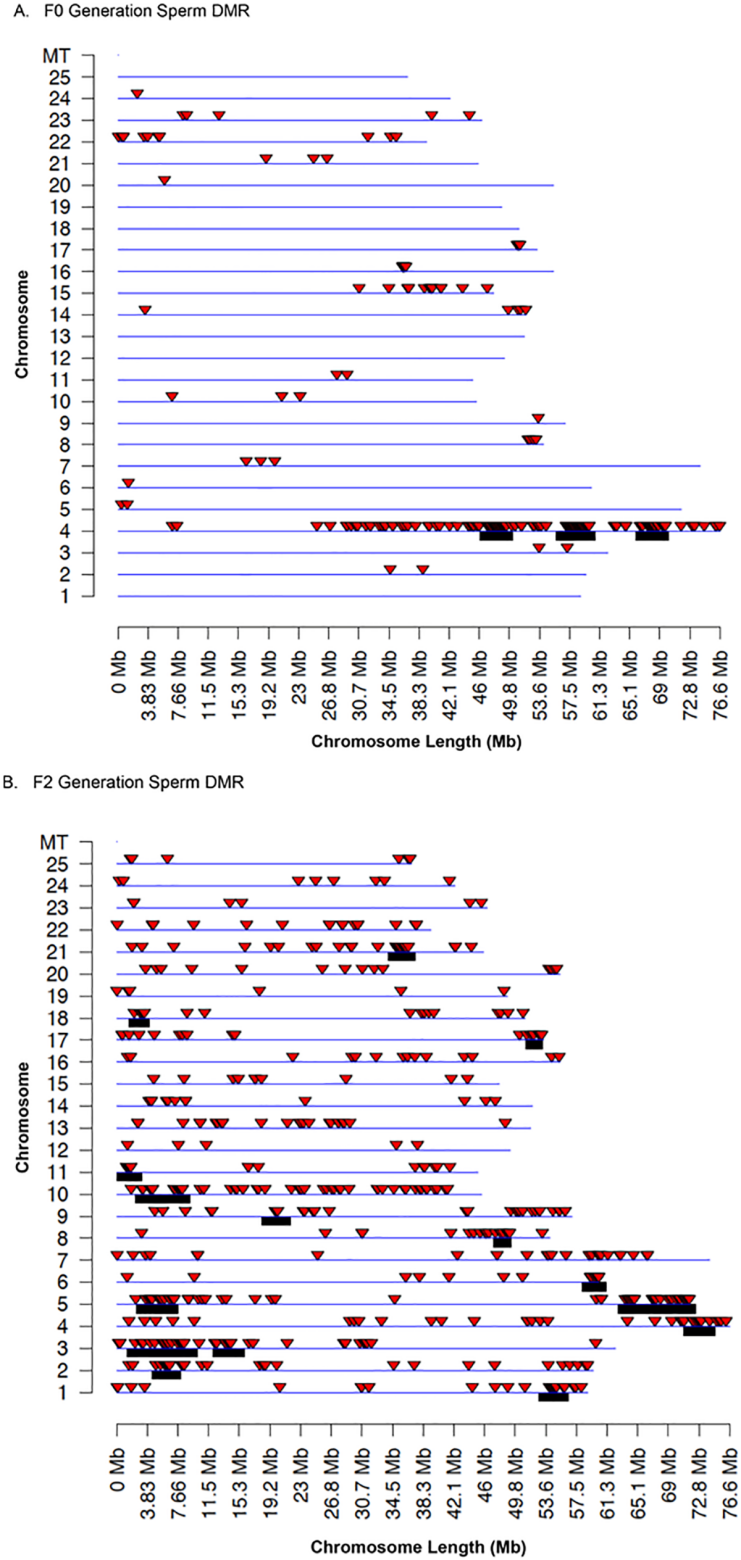
DMR chromosomal locations. (A) The F0 generation sperm DMR locations on the individual chromosomes. Only multiple window DMRs at a p-value threshold of 1e-07 are shown here. (B) The F2 generation sperm DMR locations on the individual chromosomes. Only multiple window DMRs at a p-value threshold of 1e-07 are shown here. The red arrowhead identifies the location of the DMR and black box identifies the DMR cluster site.

The chromosomal locations of the sperm multiple site DMRs on the zebrafish genome are presented in Fig 3A for the F0 generation DMRs and Fig 3B for the F2 generation DMRs. The read alignment rate was generally 90% for the zebrafish sequencing with approximately 100 million reads per pool/sample for comparison. The F0 generation DMRs were less in number with many chromosomes having DMRs, but five chromosomes not having DMRs (Fig 3A). An odd over representation of DMRs were observed on the right arm of the chromosome 4. This was presumed to be due to the previously described nature of its chromosomal structure which is enriched in repeat elements, high in GC density, impoverished of genes, late replicating and heterochromatic relative to the rest of the zebrafish genome [48]. Therefore, direct MeHg exposure altered the DNA methylation in this region due to the susceptibility to epigenetic programming. The F2 generation sperm DMRs mapped to all the chromosomes and had a higher number than the F0 generation sperm DMRs (Fig 3B). Previous studies have demonstrated that DMRs can cluster on the genome to statistically over-represented groups of DMRs [49]. Analyses of DMR clusters indicated for the F0 generation sperm DMRs only cluster on chromosome 4 which was identified with the black box below the chromosome line and size of the cluster represented (Fig 3A). A large number of DMR clusters were identified in the F2 generation sperm DMRs with some in large size clusters, (Fig 3B). A list of the DMR clusters and characteristics are presented in S5 Table.

**Fig 4:**
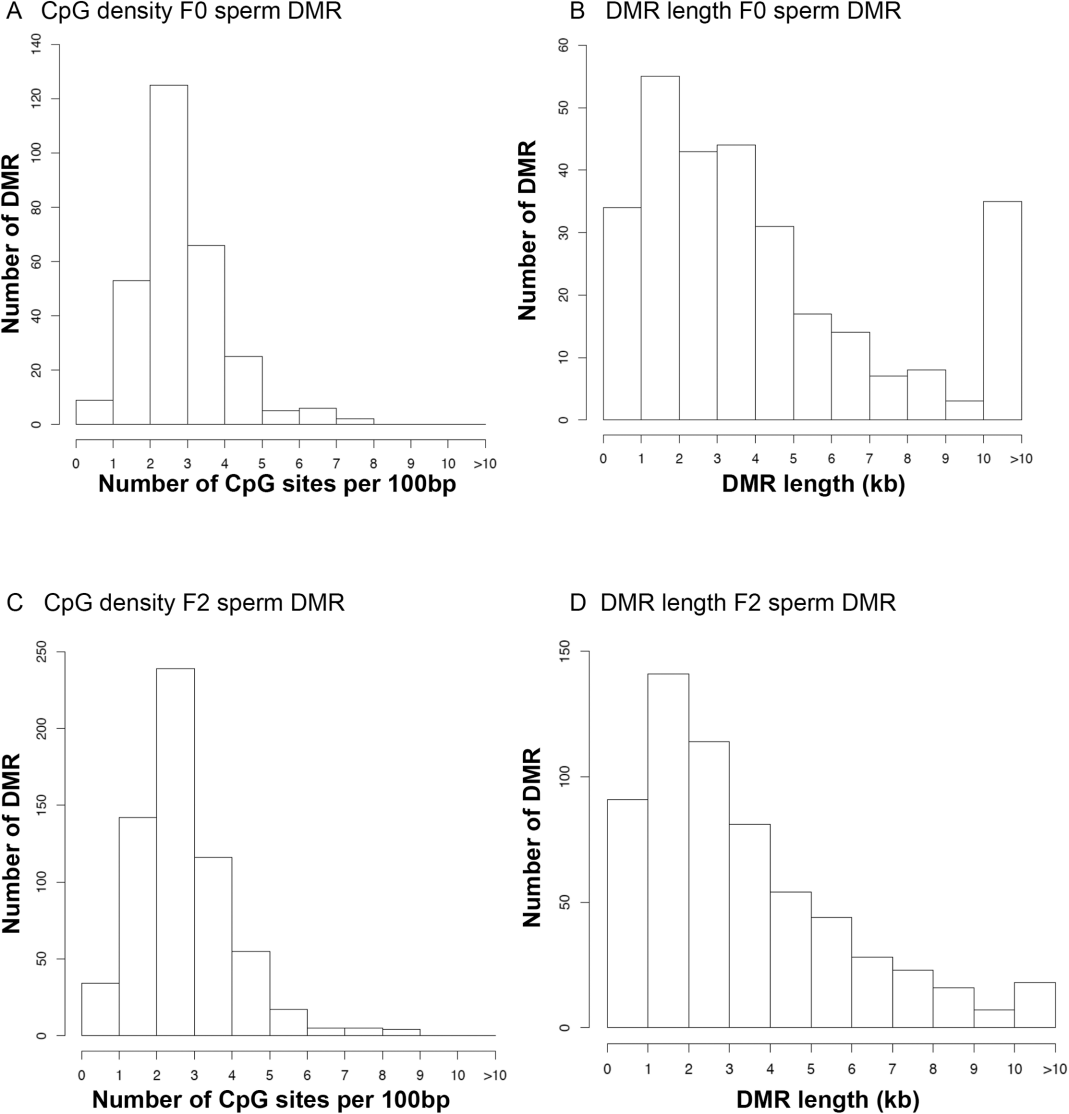
DMR CpG density and length. (A) The F0 generation sperm DMR CpG density. (B) The F0 generation sperm DMR lengths. (C) The F2 generation DMR CpG density. (D) The F2 generation DMR lengths. Only DMRs containing at least two significant sites/windows at a p-value threshold of <10^−7^.

Previous studies have demonstrated that DMRs often have low density CpG content and exist in CpG deserts [50]. Analysis of the CpG density of the DMRs identified in the current study demonstrated the DMRs also have a low density (Fig 4). The predominant CpG density in the DMR data sets was 2–4 CpG per 100 bp for both the F0 and F2 generation DMRs. In the CpG deserts of a few thousand bases, the CpG generally cluster to presumably act as regulatory sites [50]. The size of the DMRs in regards to length was found to be predominantly 1–5 kb (Fig 4B and 4D). A few DMRs were greater than 10 kb, but the majority were smaller. Therefore, the genomic features of the zebrafish sperm DMRs identified were similar to DMRs previously characterized [15, 50, 51].

Analysis of potential similarities in the DMRs between the F0 and F2 generation sperm demonstrated 13 multiple site DMRs and 135 single site DMRs with overlap. Therefore, the vast majority of DMRs are unique between the F0 and F2 generation sperm. Since the initial populations of zebrafish in the control or MeHg lineages are different between the three pools analyzed, the analysis of internal population variation in differential DNA methylation was investigated. The different pools of the population, control or MeHg, were individually pairwise compared to identify DMRs with a p<10^−7^ signature. The DMRs generated can be hypervariable DNA methylation regions, termed metastable epialleles, within the population [52]. In the control F0 generation sperm, the variable comparison of the population identified 9 potential hypervariable DMRs and in the MeHg F0 generation the pairwise comparison identified 3 hypervariable DMR (S2 Fig). In the F2 generation internal-populations analysis, 3 potential hypervariable DMRs were identified in the control lineage and 3 hypervariable DMRs in the MeHg lineage (S2 Fig). None of the hypervariable DMRs overlapped between each pairwise comparison and the control versus MeHg comparison F0 or F2 generation DMR. Therefore, internal population variation did not impact the DMRs identified between the control and MeHg lineage comparisons.

**Fig 5:**
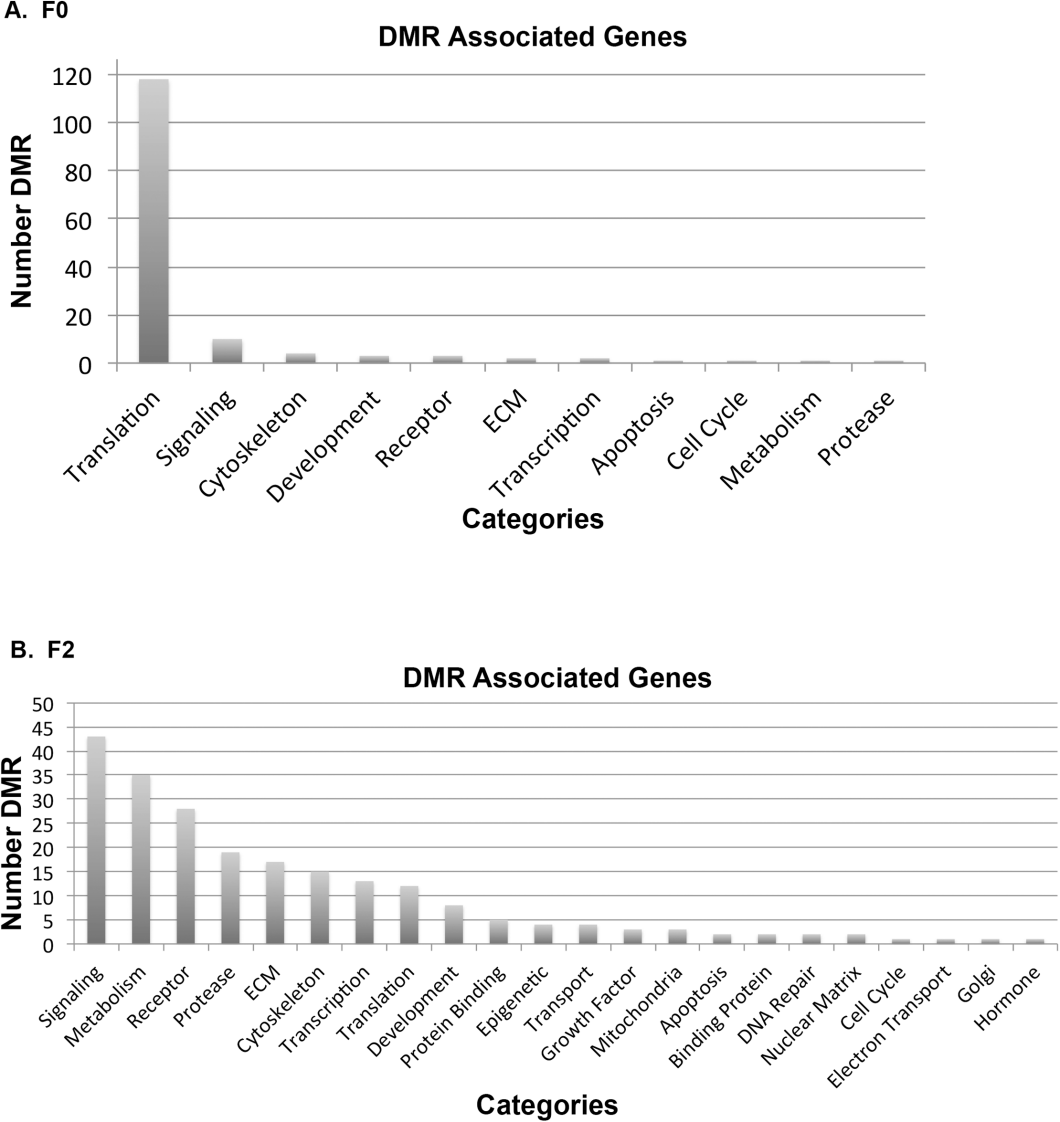
DMR association gene categories. (A) F0 generation and (B) F2 generation sperm DMR associated gene categories separated by gene classification for the sperm and number of DMR presented.

**Fig 6:**
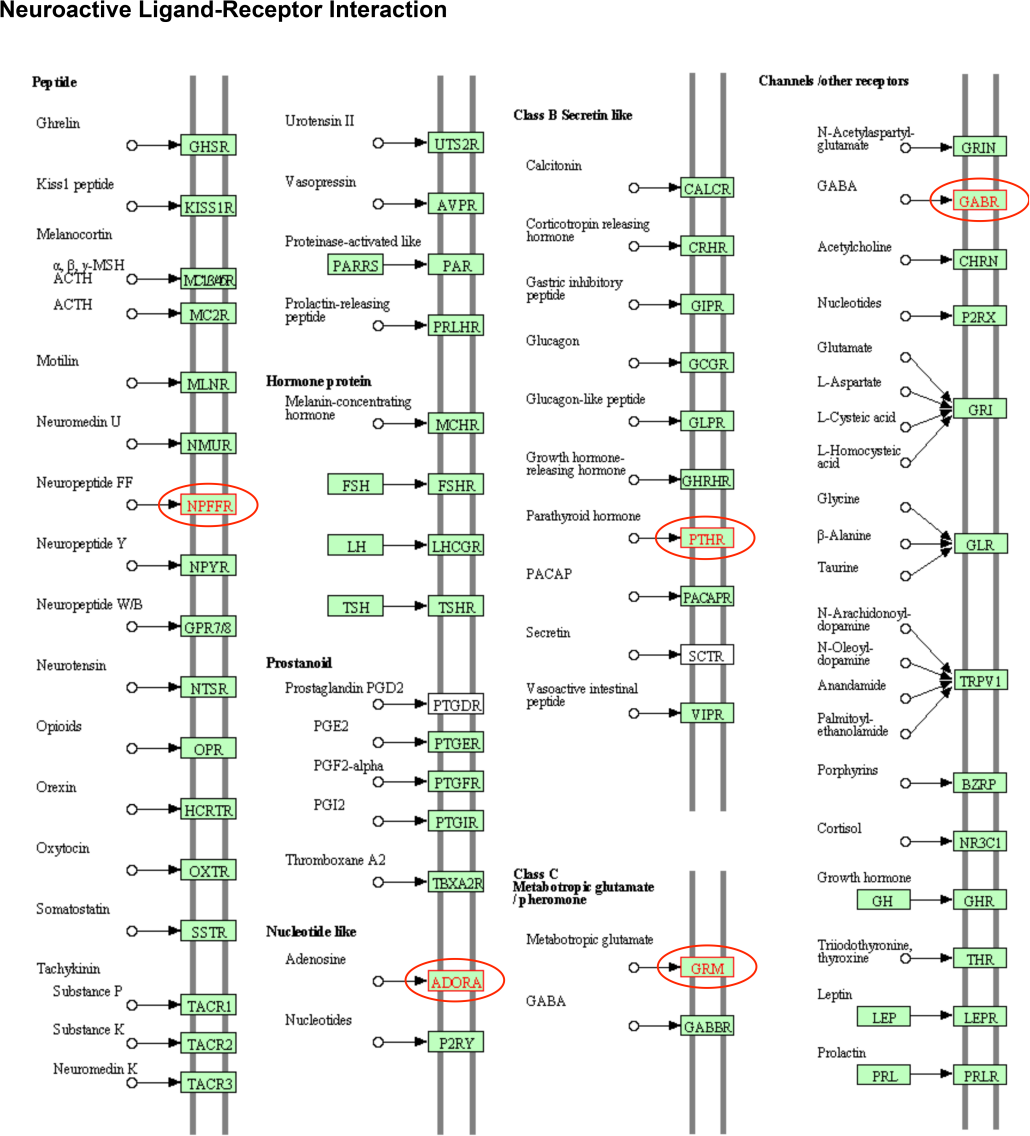
DMR associated neuroactive ligand-receptor gene pathway. The pathway with the DMR associated genes identified (circled).

The DMR associated genes were investigated using the zebrafish genome. The DMR associated genes are presented in S3 and S4 Tables with gene details presented in S6 and S7 Tables. The majority of DMRs did not have gene associations and are labeled as not applicable. The gene associations required a maximum of 10 kb distance from the known genes. The groups of gene-classification categories associated with the DMRs are shown in Fig 5. The most predominant gene category associated with the F0 generation sperm DMRs is *translation* which is primarily due to repeat elements and gene amplifications in chromosome 4 of the zebrafish involving RNH1, U1 and unknown genes. This correlates well with the DMRs clustering on chromosome 4 in the F0 generation. These epigenetic alterations on chromosome 4 were corrected by the F2 generation. When the genes are put into a pathway analysis (KEGG) no predominant pathways are observed within the F0 generation sperm DMR associated genes. In contrast, the F2 generation DMR associated genes identified signaling, metabolism, receptor and protease as major groups of genes (Fig 5B and S7 Table). The major gene pathways identified were the metabolic pathway with 16 genes, neuroactive ligand-receptor interactions with 6 genes, regulation of actin cytoskeleton with 5 genes, focal adhesion with 4 genes, and MAPK signaling pathway with 4 genes. The neuroactive ligand-receptor interaction pathway and DMR associated genes are shown in Fig 6 and the regulation of actin cytoskeleton pathway in S2 Fig. Therefore, the DMR data sets identified have the capacity to regulate genome activity (Fig 5) and are relevant to the abnormal neurobehavioral phenotypes observed (Fig 6 and S2 Fig).

## Discussion

Significant literature exists on the neurotoxicity of MeHg upon the direct exposure of humans [35, 53] and animal models [12, 13, 54]. For instance, Weber *et al*. [12] showed that direct developmental exposure (*ex vivo* from <4 to 24 hpf) of F0 generation zebrafish embryos to MeHg (10, 30, 60, 100, and 300 nM) yielded abnormal vision-related phenotypes in adult F0 generation zebrafish analogous to those observed in the F0 and F2 generations in the current study, compared to control. Moreover, Smith *et al*. [13] employed the same exposure paradigm (except the exposure was from 2–24 hpf) and demonstrated that direct developmental MeHg exposure caused learning deficits in all MeHg-exposure groups relative to control. In conclusion, these results in tandem with those of the current study (which included the same route of exposure with inclusion of even lower MeHg exposures [*i*.*e*., 1 and 3 nm]) reveal that developmental MeHg can elicit learning and visual deficits in zebrafish, a model of human health. However, studies investigating the epigenetic transgenerational inheritance of Hg-induced neurotoxicity have not been previously reported.

Several studies have shown that direct MeHg exposure can promote epigenetic effects via alterations in DNA methylation *in vitro* [55] and *in vivo* [56, 57]. Developmental (*i*.*e*., *in utero*) MeHg exposure has been shown to yield gene-specific hypomethylation in newborn infants [56]. Low MeHg-exposures (2.5 nM and 5 nM) were shown to decrease cell proliferation and dysregulate gene expression related to cell cycle [*i*.*e*., *p16* and *p21*], mitochondria function [*i*.*e*., *Nd3* and *Cytb*], and senescence [*i*.*e*., *Bmi1*]) in directly exposed neural stem cells *in vitro* [55]. Global DNA hypomethylation with concurrent downregulation of DNA methyltransferase 3b (*Dnmt3b)* was also observed MeHg-exposed cells and persisted in the daughter cells after removal of MeHg. The dysregulation of genes associated with reduced cell proliferation (*i*.*e*., *p16*, *p21*, *Nd3*, *Cytb*, and *Bmi1*) also persisted in the daughter cells. Presented here is the first report that developmental exposure of zebrafish to MeHg can promote the epigenetic transgenerational inheritance of phenotypes (*i*.*e*., visual deficits, hyperactivity, and altered retinal electrophysiology) in correlation with sperm epimutations.

Dosimetric analysis of THg in each MeHg-exposed F0 generation lineage (S1 Table) demonstrated that the mean internal doses of Hg in the lower exposure lineages were 19, 51, 257, 836, and 2819 ppb (respectively for 1, 3, 10, 30, and 100 nM). The USEPA reference dose for MeHg (the level that is likely to be without appreciable risk) is equivalent to about 5.8 ppb in maternal blood and 3.5 ppb in umbilical cord blood [58]. THg in blood samples was not measured in the current study due to pragmatic considerations. Alternatively, THg from whole zebrafish embryo was measured to allow rough comparison to concentrations in human umbilical cord blood. Eleven percent of women of child-bearing age in the United States have levels in excess of the reference dose, and that number rises to 27% among self-identified Asian, Pacific Islander, Native American, or multiracial women; indicating that their children might be at risk from the effects of MeHg toxicity [58]. Moreover, Boucher *et al*. [59] have reported that prenatal MeHg exposure (range of umbilical cord blood concentration = 11.4 to 99.3 ppb) is associated with symptoms of attention deficit, hyperactivity disorder (ADHD) in school-age Inuit children (n = 179) from artic Canada. Analysis of umbilical cord blood spots from the Lake Superior region (Northern Minnesota, Wisconsin, Michigan) reveal that 1% of newborns in that population have levels higher than 58 ppb, with a maximum concentration of 211 ppb [34]. Prenatally exposed humans (n = 278) from the poisoning event of Minamata, Japan, yielded an umbilical cord concentration of 590 ± 750 (mean ± SD) ppb Hg [35], which was associated with neurocognitive dysfunction later in life [60]. Collectively, this evidence supports that the measured internal doses of Hg in the 1, 3, 10, and 30-nM exposure lineages are within the range of human relevancy.

THg levels in all F2 generation lineages were identical, as expected, supporting the role of epigenetics in the inheritance of the phenotypes rather than chronic MeHg exposure across generations. However, the tissue dose of the F2 generation lineages (~9.0 ppb) were greater than the F0 generation control lineage (~5.0 ppb). It is reasonable to postulate that the higher tissue dose of THg in the F2 generation lineage is likely due to the endogenous levels of Hg in the food fed to the fish in current study. The platinum and gold grade *Artemia* nauplii fed to the zebrafish, from larva to adult, contain Hg since the *Artemia* were harvested from aquatic environments and that background level can vary lot-by-lot [61]. All lineages (1, 3, 10, 30, and 100 nM) exhibited an overall significant increase in spontaneous locomotion (*i*.*e*., hyperactivity) and a significant decrease in visual startle (*i*.*e*., visual deficit) compared to control. The visual deficit and altered retinal electrophysiology observed in the F2 generation were similar to the observations in our previous study that employed a direct developmental MeHg-exposure (F0 generation) [12]. Robust statistical analyses (ANOVA) yielded a statistically significant alteration in each neurobehavior (compared to control), but phenotypic variation was very apparent among the individuals of each F2 generation lineage. This individual variability may be due to the fact that our EK zebrafish population has been intentionally maintained as a genetically diverse, highly polymorphic population [48] analogous to that of humans. Genetic diversity can impact an individual’s susceptibility to toxicity upon exposure to an environmental contaminant and, the observed phenotype. For instance, Llop *et al*. [62] have proposed that inherent variation in genes related to Hg toxicokinetics (*i*.*e*., metabolism, excretion, and distribution) mediates the half-life of Hg and, in turn, the degree of molecular impact (*i*.*e*., toxicodynamics), which subsequently yields the variation in individual neurobehavioral phenotypes. The genetic diversity of our zebrafish population likely contributed to the phenotypic variation between individuals. Additionally, it was determined that the MeHg-induced hyperactivity and visual deficits observed in the present study are independently inherited phenotypes. This evidence implies that multiple, independent molecular pathways were disrupted by F0 generation epimutations in the MeHg lineages. Importantly, the persistence of visual deficit and/or hyperactivity in the F2 generation suggests that the MeHg-induced the epigenetic transgenerational inheritance of sperm epimutations and similar phenotypic alterations.

The epigenetic alterations observed involved differential DMRs identified in the germline (sperm). The presence of a reproducible DMR is termed an epimutation [14–16], The DMRs identified have a low CpG density and exist in CpG deserts [50]. Evolutionarily CpG methylation has been shown to increase the susceptibility of C to T conversions and lead to regions of the genome with low CpG content. Small clusters of CpG in these CpG deserts are likely conserved due to the regulatory roles of these CpG clusters [50]. The genome-wide locations of the DMRs also demonstrate a genome-wide distribution on most chromosomes. The comparison of the control and MeHg F0 and F2 generation lineages identified a large number of single site DMRs and, using a more stringent selection of multiple sites, yielded a highly reproducible set of DMRs. The internal population variation for hypervariable DMRs did not have a significant contribution on the DMR sets identified. In addition, negligible genetic mutations, copy number variation (CNV), were identified and had no impact on the DMR identified. Interestingly, the F0 generation sperm DMRs had lower numbers than the F2 generation sperm DMRs and negligible overlap. The F0 generation had a large cluster of DMRs on chromosome 4 that is thought to be due to its nature as a late replicating, heterochromatic chromosome with abundant repeat elements, high GC density, and a general impoverishment of protein-coding genes [48]. The direct exposure alterations of the DNA methylation in this region were apparently corrected during epigenetic programming at the next generation and was not present in the F2 generation sperm DMRs. Therefore, the direct exposure effects of MeHg on sperm DMRs in the F0 generation are distinct from the epigenetic programming observed in the transgenerational generations when the epimutations are protected from DNA erasure after fertilization [14]. Previously, number and location of direct exposure DMRs and transgenerational DMRs have been shown to be distinct [14]. The F2 generation sperm DMRs did have a large number of DMR clusters as previously observed [15]. These DMR clusters may provide critical regulatory regions to influence genome activity. Observations demonstrate MeHg can promote the epigenetic transgenerational inheritance of sperm epimutations. Although the current study focused on DNA methylation, other epigenetic processes such as ncRNA and histone modifications will also likely be involved in the transgenerational phenomenon [14]. An integration of epigenetic processes and genetics [63] will be involved and needs to be further assessed.

The DMR associated genes indicate that the epigenetic alterations observed can have major effects on genome activity and potentially correlate to the phenotypic alterations observed. The majority of DMRs did not have gene associations as previously observed in other transgenerational models [64]. Previous studies have shown the clustering of genes and DMRs in epigenetic control regions (ECR) [49]. These ECR are speculated to provide distal gene regulation through intermediates such as ncRNA. The currently identified DMR clusters in the F2 generation sperm DMRs may provide such distal regulation of gene expression (S3 Table). A large number of F2 generation MeHg lineage DMR associated genes were identified and the predominant classification categories included signaling, receptor and metabolism. Associated pathways included metabolic pathways, neuroactive receptor-ligand interactions, actin cytoskeleton, focal adhesion, and MAPK. Interestingly, the neuroactive ligand-receptor interaction (Fig 6) and actin cytoskeleton pathways (S3 Fig) may correlate to the behavioral phenotypes observed via the possible MeHg-mediated disruption of neuronal and/or retinal signaling, neuromuscular junctions, and/or muscle physiology. The DMR associated genes are speculated to impact the F2 generation phenotype and health effects observed.

The current study demonstrates the ability of MeHg to promote the epigenetic transgenerational inheritance of phenotypic alterations and sperm epimutations in zebrafish. The molecular data obtained suggests a more dramatic and reproducible alteration in epigenetics than genetics. The observations correlate with the phenotypic alterations promoted by MeHg exposure. Although reproducible signatures of DMRs were found, the functional impacts of these DMRs need to be further investigated. Clearly, the current study supports a critical role of epigenetics in the transgenerational impacts of MeHg. However, it will be an integration of epigenetics and genetics that will influence the molecular control of phenotypic variation and health [63]. The current study provides the first observation that a heavy metal can promote the epigenetic transgenerational inheritance of health effects. Heavy metals such as Hg need to be added to the growing list of environmental exposures that can promote transgenerational effects [14].

## Conclusions

Future studies focused on elucidating the transgenerational actions of heavy metals is required to advance our understanding of potentially adverse health effects so that national and local governments and their communities can make informed decisions about this exposure risk. In recent years, international (*i*.*e*., Minamata Convention on Mercury; http://mercuryconvention.org/) and domestic (*i*.*e*., Mercury and Air Toxics Standards; http://www3.epa.gov/airquality/powerplanttoxics/) regulatory policies focused on reducing Hg emissions have been adopted. However, the potential generational impacts of Hg exposures on humans or other species were not considered in these policies. Consequently, the continued emergence of scientific research focused on elucidating the inherited health impacts of heavy metals will fortify generational environmental justice issues [65] that will demand consideration in future public policy and legislation for the sake of public health.

## Supporting information

S1 Fig Change in mean peak amplitude of depolarization-elicited outward potassium currents recorded from bipolar cells in retinas from F2 zebrafish ancestrally exposed to various concentrations of MeHg. Both I_K_ **(A, C)** and I_A_ **(B, D)** currents were recorded. Representative whole-cell **(A)** I_K_ and **(B)** I_A_ current traces recorded from bipolar cells in control, 10 nM, 30 nM, and 100 nM MeHg exposure groups. Current-voltage relationship of **(C)** I_K_ and **(D)** I_A_ currents showing the mean peak currents elicited at different voltage steps from a holding potential of -60 mV. For I_K_, a one-way ANOVA comparing peak current at a voltage step to +60 mV was not significant (df = 46, F = 2.256, p = 0.095), due to the reduction in current amplitude seen in the 100 nM exposure (open triangles). If this high exposure is removed from the analysis, ANOVA results become significant (df = 34, F = 3.601, *p = 0.039), with a larger amplitude in the 30 nM group (solid triangles) compared to control (solid circles) or the 10 nM group (open circles). There was also a significant linear trend of increasing current amplitude with increasing MeHg exposure concentration for the control, 10 nM, and 30 nM exposure groups (df = 34, F = 7.175, p = 0.012). For the I_A_ current, a one-way ANOVA comparing peak currents at a voltage step to +60 mV was also not significant (df = 26, F = 2.32, p = 0.102). However, there was a significant linear trend in the data (df = 21, F = 6.256, p = 0.022) of increasing current amplitude with increasing MeHg exposure concentration (control, 10 nM, and 30 nM groups only). Samples sizes (n = number of individually recorded retinal bipolar cells) for these analyses are identical to Table 1.
(PDF)

S2 Fig Internal population DMR variation. Pairwise comparison of individual pools for control or MeHg treated for the F0 generation **(A)** and F2 generation **(B)** are presented with number of DMR with multiple sites at p<10^−7^ presented and compared to the full analysis of control vs. MeHg. The Venn diagrams demonstrate the overlap for each and no overlap is observed between all three pairwise comparisons and the full analysis.
(PDF)

S3 Fig Regulation of actin cytoskeleton gene pathway. The DMR associated genes are circled within the pathway.
(PDF)

S1 Table Total Hg analysis and evaluation of dosimetry. All values are represented as mean ± SEM. Measured exposure: n = 3; F0 generation tissue dose: n = 9; F2 generation tissue dose: n = 3. One-way analysis of variance showed no significant difference regarding the tissue dose of total Hg present in the F2 generation MeHg lineages compared to negative control (df = 17, F = 1.449, p = 0.276).
(PDF)

S2 Table Evaluation of expected versus observed inheritance of neurobehavioral phenotypes. The control group (0.0 nM MeHg) was excluded from the chi-square analysis because the number of expected and observed animals to have both neurobehavioral abnormalities was zero. Numbers in parentheses represent the number of fish in each cohort.
(PDF)

S3 Table F0 generation sperm multiple site DMR list. The DMR name, chromosome number, DMR start site, length in base pair (bp), number of multiple sites, minimum p-value, CpG number per sequence length, CpG density (CpG number/100 bp) and DMR gene association with the symbol listed and NA indicating not applicable with gene but no name.
(PDF)

S4 Table F2 generation sperm multiple site DMR list. The DMR name, chromosome number, DMR start site, length in base pair (bp), number of multiple sites, minimum p-value, CpG number per sequence length, CpG density (CpG number/100 bp) and DMR gene association with the symbol listed and NA indicating not applicable with gene but no name.
(PDF)

S5 Table DMR cluster analysis lists. Information for **(A)** F0 generation DMR cluster sperm list, and **(B)** F2 generation DMR cluster sperm list. The DMR cluster is presented with the specific DMR in the cluster listed, chromosome number, start and stop sites, cluster size and minimum p-value of the cluster identification.
(PDF)

S6 Table F0 generation sperm DMR associated genes. The DMR name, gene symbol, entrez gene identification, chromosome number, start position site, ensemble gene identifications, gene description, and gene classification category are presented.
(PDF)

S7 Table F2 generation sperm DMR associated genes. The DMR name, gene symbol, entrez gene identification, chromosome number, start position site, ensemble gene identifications, gene description, and gene classification category are presented.
(PDF)
