# Supplementary figures and images for: Mercury-induced epigenetic transgenerational inheritance of abnormal neurobehavior is correlated with sperm epimutations in zebrafish
Source: PLoS One. 2017 May 2;12(5):e0176155. doi: 10.1371/journal.pone.0176155 (PMC5413066; doi:10.1371/journal.pone.0176155)

Supplemental Figure S1

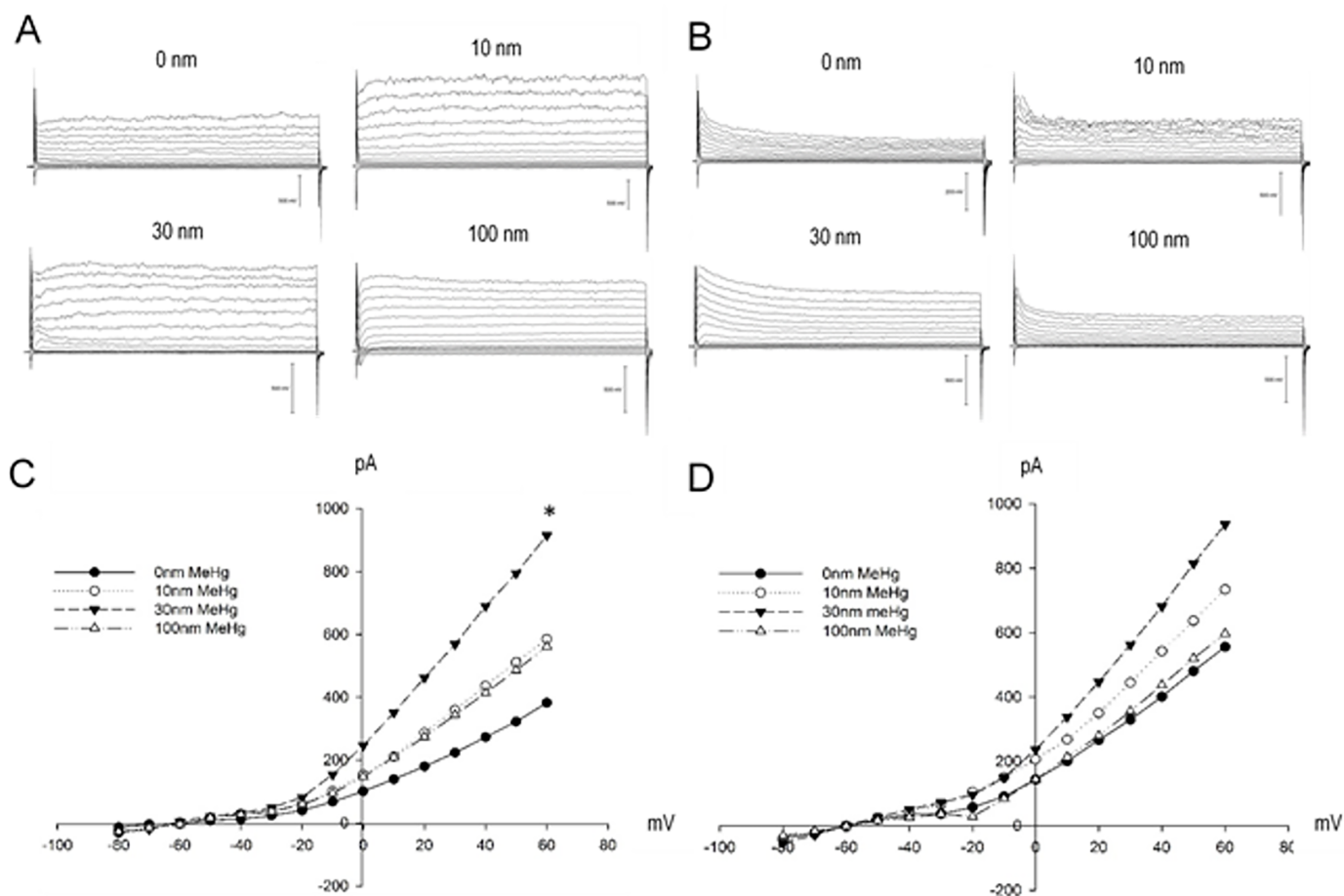

Supplement: S1 Fig — Both IK (A, C) and IA (B, D) currents were recorded. Representative whole-cell (A) IK and (B) IA current traces recorded from bipolar cells in control, 10 nM, 30 nM, and 100 nM MeHg exposure groups. Current-voltage relationship of (C) IK and (D) IA currents showing the mean peak currents elicited at different voltage steps from a holding potential of -60 mV. For IK, a one-way ANOVA comparing peak current at a voltage step to +60 mV was not significant (df = 46, F = 2.256, p = 0.095), due to the reduction in current amplitude seen in the 100 nM exposure (open triangles). If this high exposure is removed from the analysis, ANOVA results become significant (df = 34, F = 3.601, *p = 0.039), with a larger amplitude in the 30 nM group (solid triangles) compared to control (solid circles) or the 10 nM group (open circles). There was also a significant linear trend of increasing current amplitude with increasing MeHg exposure concentration for the control, 10 nM, and 30 nM exposure groups (df = 34, F = 7.175, p = 0.012). For the IA current, a one-way ANOVA comparing peak currents at a voltage step to +60 mV was also not significant (df = 26, F = 2.32, p = 0.102). However, there was a significant linear trend in the data (df = 21, F = 6.256, p = 0.022) of increasing current amplitude with increasing MeHg exposure concentration (control, 10 nM, and 30 nM groups only). Samples sizes (n = number of individually recorded retinal bipolar cells) for these analyses are identical to Table 1. (PDF) [file pone.0176155.s001.pdf]

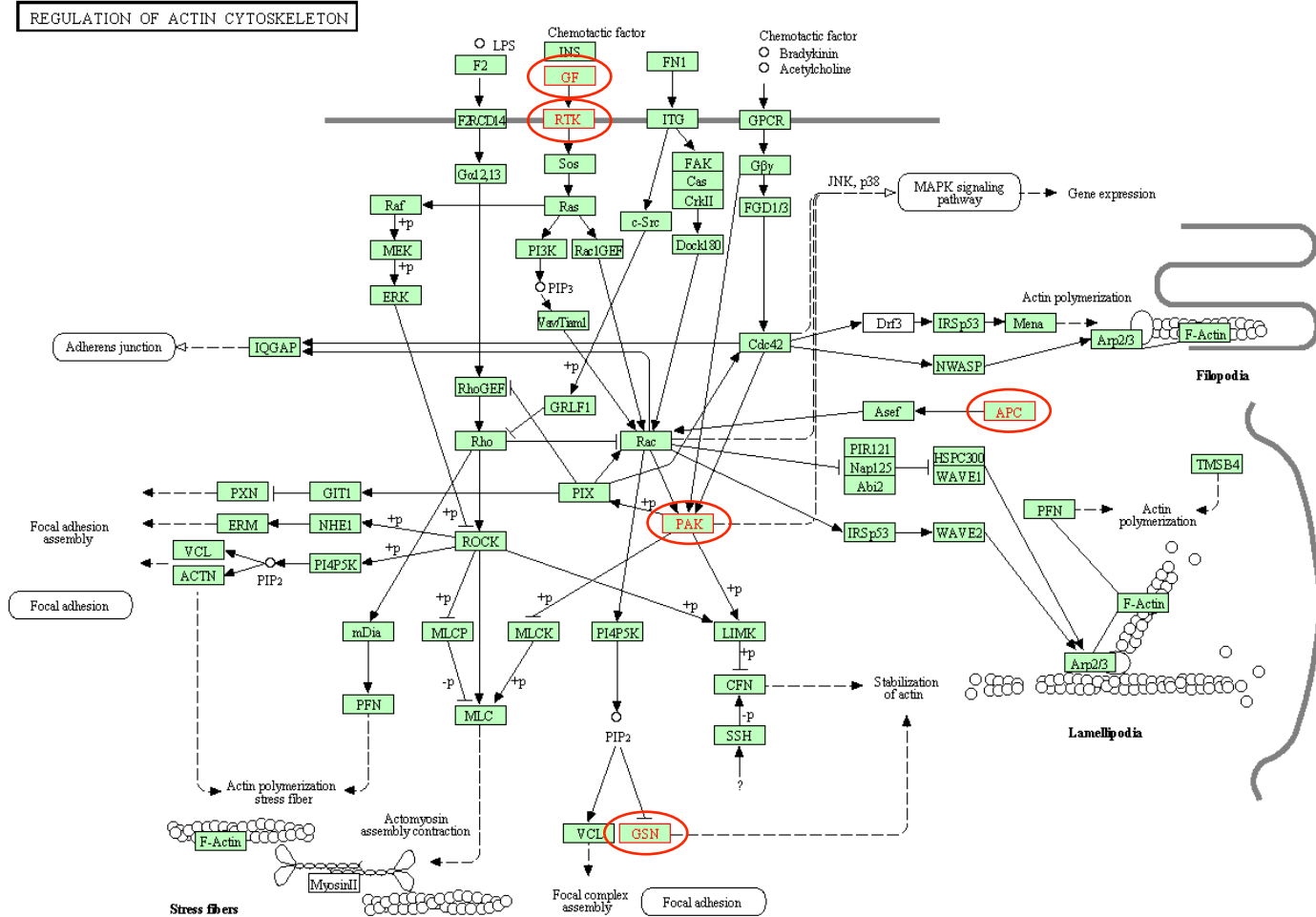

Supplement: S3 Fig — The DMR associated genes are circled within the pathway. (PDF) [file pone.0176155.s003.pdf]
