# Supplementary material for: Mercury-induced epigenetic transgenerational inheritance of abnormal neurobehavior is correlated with sperm epimutations in zebrafish
Source: PLoS One. 2017 May 2;12(5):e0176155. doi: 10.1371/journal.pone.0176155 (PMC5413066; doi:10.1371/journal.pone.0176155)
Supplement: S2 Fig — Pairwise comparison of individual pools for control or MeHg treated for the F0 generation (A) and F2 generation (B) are presented with number of DMR with multiple sites at p<10−7 presented and compared to the full analysis of control vs. MeHg. The Venn diagrams demonstrate the overlap for each and no overlap is observed between all three pairwise comparisons and the full analysis. (PDF) [file pone.0176155.s002.pdf]

Supplemental Figure S2

A. MeDIP pairwise internal population analysis summary F0 generation

Analysis

Full Analysis	C1vsC2	C1vsC3	C2vsC3	T1vsT2	T1vsT3	T2vsT3
291	724	423	541	403	338	349

The number of DMR found for each analysis. All DMR have at least two significant windows at a p-value threshold of 1e-07.

Overlapping DMR

The following figures show the number of overlapping DMR between each pairwise analysis and the full analysis

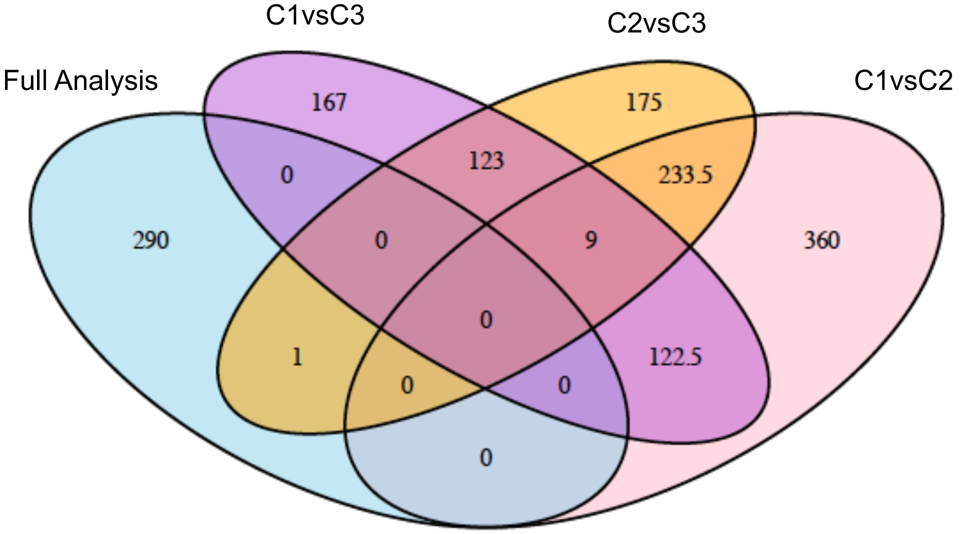

This figure shows the overlapping DMR between the control vs. control pairwise analyses and the full analysis.

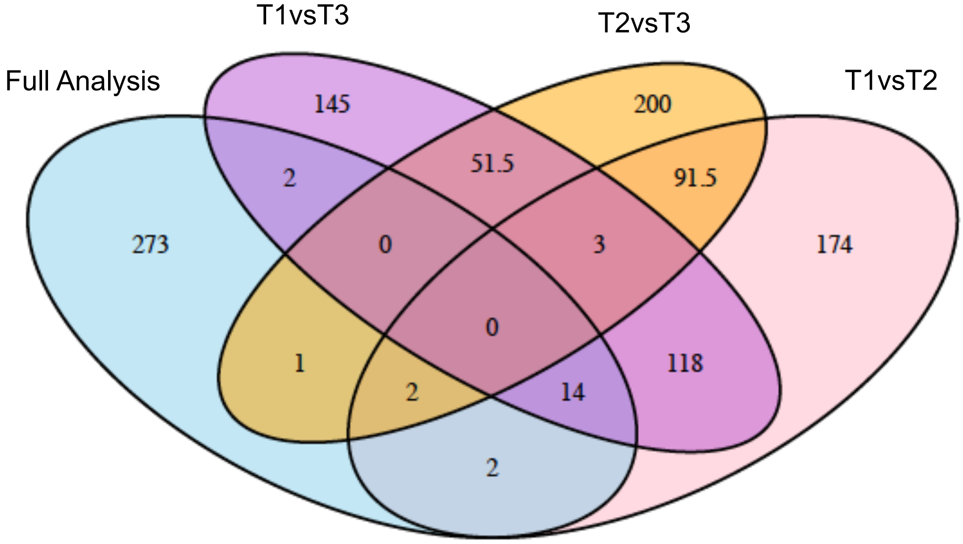

This figure shows the overlapping DMR between the treatment vs. treatment pairwise analyses and the full analysis.

Supplemental Figure S2 (continued)

B. MeDIP pairwise internal population analysis summary F2 generation

Analysis

Full Analysis	C1vsC2	C1vsC3	C2vsC3	T1vsT2	T1vsT3	T2vsT3
617	865	298	436	774	394	353

The number of DMR found for each analysis. All DMR have at least two significant windows at a p-value threshold of 1e-07.

Overlapping DMR

The following figures show the number of overlapping DMR between each pairwise analysis and the full analysis

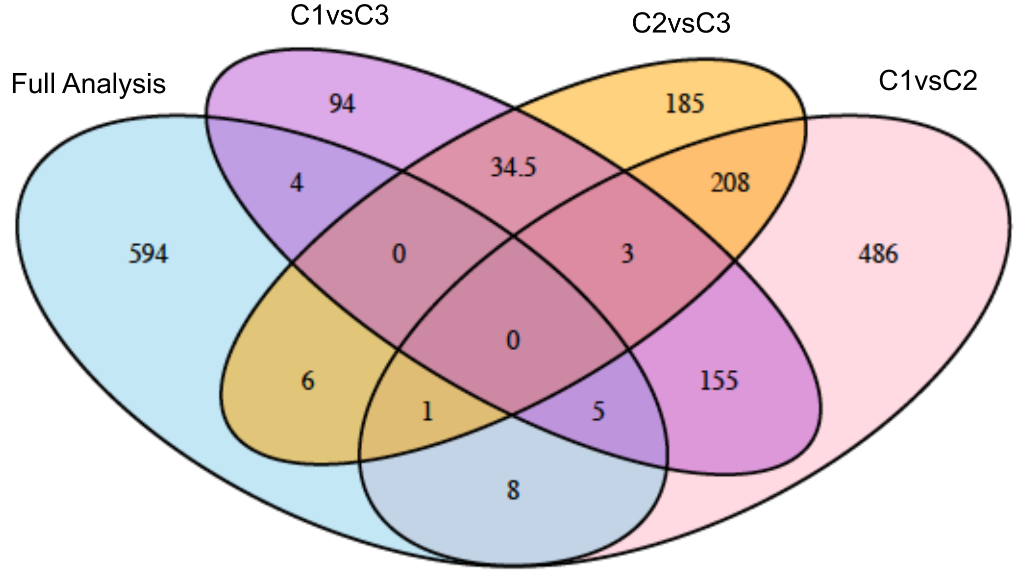

This figure shows the overlapping DMR between the control vs. control pairwise analyses and the full analysis.

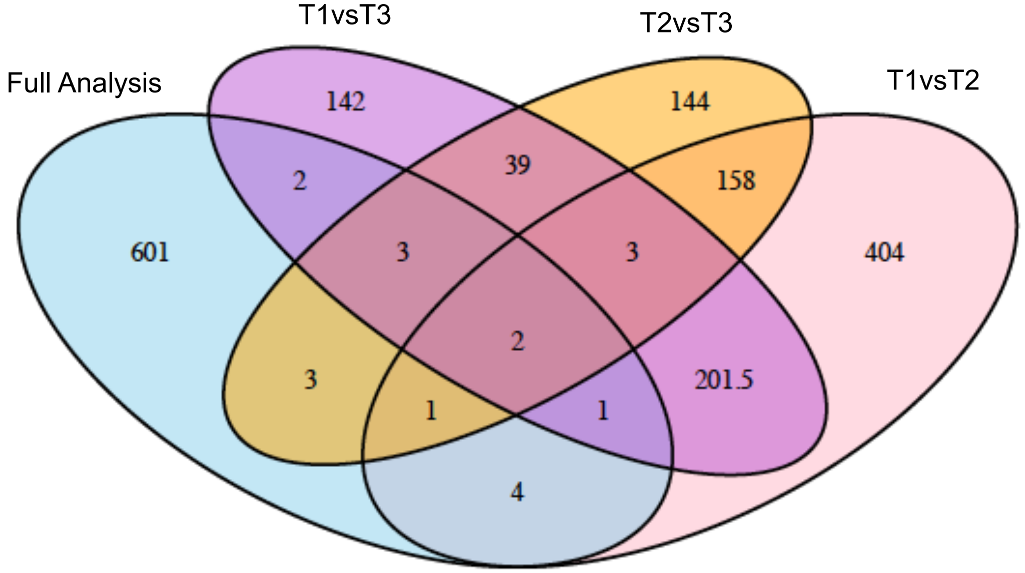

This figure shows the overlapping DMR between the treatment vs. treatment pairwise analyses and the full analysis.
