# Supplementary material for: Mercury-induced epigenetic transgenerational inheritance of abnormal neurobehavior is correlated with sperm epimutations in zebrafish
Source: PLoS One. 2017 May 2;12(5):e0176155. doi: 10.1371/journal.pone.0176155 (PMC5413066; doi:10.1371/journal.pone.0176155)
Supplement: S1 Table — All values are represented as mean ± SEM. Measured exposure: n = 3; F0 generation tissue dose: n = 9; F2 generation tissue dose: n = 3. One-way analysis of variance showed no significant difference regarding the tissue dose of total Hg present in the F2 generation MeHg lineages compared to negative control (df = 17, F = 1.449, p = 0.276). (PDF) [file pone.0176155.s004.pdf]

**Supplemental Table S1.** Total Hg Analysis and Evaluation of Dosimetry

| Exposure (nM) | | Tissue Dose (ppb) | |
|---|---|---|---|
| Nominal | Measured | F0 generation | F2 generation |
| 0 | 0.15 ± 0.02 | 5.5 ± 0.5 | 9.0 ± 0.4 |
| 1 | 1.5 ± 0.4 | 19.4 ± 1.0 | 8.2 ± 0.2 |
| 3 | 2.9 ± 0.2 | 51.0 ± 3.5 | 9.2 ± 0.1 |
| 10 | 10 ± 0.05 | 257.4 ± 10.7 | 9.1 ± 0.2 |
| 30 | 32 ± 0.6 | 836.1 ± 67.8 | 8.7 ± 0.4 |
| 100 | 104 ± 0.4 | 2819.2 ± 152.4 | 8.5 ± 0.4 |
