# Supplementary material for: Mercury-induced epigenetic transgenerational inheritance of abnormal neurobehavior is correlated with sperm epimutations in zebrafish
Source: PLoS One. 2017 May 2;12(5):e0176155. doi: 10.1371/journal.pone.0176155 (PMC5413066; doi:10.1371/journal.pone.0176155)
Supplement: S2 Table — The control group (0.0 nM MeHg) was excluded from the chi-square analysis because the number of expected and observed animals to have both neurobehavioral abnormalities was zero. Numbers in parentheses represent the number of fish in each cohort. (PDF) [file pone.0176155.s005.pdf]

Supplemental Table S2: Evaluation of expected versus observed inheritance of neurobehavioral phenotypes.

F2 lineage (nM)	n	Neither Phenotype	Visual deficit	Hyperactivity	Both Phenotypes		*Chi-square*
					Expected	Observed	
0	15	87% (13)	7% (1)	7% (1)	0% (0)	0% (0)	
1	20	5% (1)	65% (13)	65% (13)	42% (8)	35% (7)	
3	16	6% (1)	93% (14)	38% (6)	35% (6)	31% (5)	*χ^2=0.0 670 df=4 p=0.999*
10	18	6% (1)	72% (13)	61% (11)	44% (8)	39% (7)	
30	17	6% (1)	71% (12)	71% (12)	50% (9)	47% (8)	
100	15	0% (0)	93% (14)	60% (9)	56% (8)	53% (8)	
