# Supplementary material for: Mercury-induced epigenetic transgenerational inheritance of abnormal neurobehavior is correlated with sperm epimutations in zebrafish
Source: PLoS One. 2017 May 2;12(5):e0176155. doi: 10.1371/journal.pone.0176155 (PMC5413066; doi:10.1371/journal.pone.0176155)
Supplement: S3 Table — The DMR name, chromosome number, DMR start site, length in base pair (bp), number of multiple sites, minimum p-value, CpG number per sequence length, CpG density (CpG number/100 bp) and DMR gene association with the symbol listed and NA indicating not applicable with gene but no name. (PDF) [file pone.0176155.s006.pdf]

Supplemental Table S3 F0 Generation DMR List

DMR Name	Chr	Start	Length (bp)	# Signature Windows	min P Value	CpG #	CpG Density (#/100bp)	Gene Association
DMR2:34637701	2	34637701	2100	2	3.64E-09	74	3.5	astn1
DMR2:38796201	2	38796201	400	2	6.31E-10	5	1.2	
DMR3:53595001	3	53595001	1900	4	2.71E-10	52	2.7	col5a3a
DMR3:57175901	3	57175901	4800	2	5.66E-09	143	2.9	fscn2a
DMR4:6914401	4	6914401	1200	4	4.42E-10	14	1.1	dock4b
DMR4:7479801	4	7479801	1600	4	1.36E-17	16	1	cald1l2
DMR4:25320901	4	25320901	900	4	7.17E-11	12	1.3	sfmbt2
DMR4:26986301	4	26986301	5600	4	1.38E-09	260	4.6	slc6a1l
DMR4:29125601	4	29125601	4600	3	3.10E-10	169	3.6	fb11h05
DMR4:29131601	4	29131601	3500	3	1.35E-16	75	2.1	fb11h05
DMR4:29170301	4	29170301	800	3	2.77E-12	22	2.7	
DMR4:29690601	4	29690601	4300	8	9.97E-34	60	1.3	ch211-214c20.1
DMR4:30279501	4	30279501	1200	4	3.88E-11	29	2.4	RNH1 (6 of 55)
DMR4:30506601	4	30506601	1900	4	2.87E-11	37	1.9	dkey-199m13.6
DMR4:31576001	4	31576001	1700	3	3.49E-10	40	2.3	
DMR4:32114901	4	32114901	8800	3	6.80E-10	261	2.9	
DMR4:32127101	4	32127101	1400	2	9.85E-11	58	4.1	
DMR4:33270701	4	33270701	2000	5	3.76E-11	59	2.9	
DMR4:33640001	4	33640001	500	3	5.12E-20	6	1.2	
DMR4:33980601	4	33980601	1500	3	5.19E-09	26	1.7	dkey-28i19.3
DMR4:34064901	4	34064901	1700	3	4.20E-23	101	5.9	U1
DMR4:34967901	4	34967901	2300	3	2.51E-30	71	3	
DMR4:34980901	4	34980901	10800	2	2.46E-15	360	3.3	
DMR4:36150301	4	36150301	1800	5	1.29E-14	53	2.9	
DMR4:36156101	4	36156101	800	3	1.40E-11	18	2.2	
DMR4:36157901	4	36157901	2300	6	1.52E-12	42	1.8	
DMR4:36504301	4	36504301	3500	9	6.10E-12	72	2	dkeyp-87d1.10
DMR4:36909601	4	36909601	14200	8	3.82E-14	516	3.6	
DMR4:37866401	4	37866401	2200	2	6.86E-15	48	2.1	RNH1 (39 of 55)
DMR4:39532701	4	39532701	5400	3	2.64E-10	112	2	dkey-16p6.1
DMR4:39614001	4	39614001	1600	7	5.61E-20	36	2.2	174700
DMR4:40514901	4	40514901	900	2	2.90E-09	37	4.1	
DMR4:40931001	4	40931001	4200	9	9.30E-17	117	2.7	ch211-231i17.4
DMR4:42207201	4	42207201	1600	4	2.95E-14	62	3.8	
DMR4:43184201	4	43184201	5200	2	7.77E-09	162	3.1	ch211-226o13.1
DMR4:44610401	4	44610401	6700	8	7.06E-16	308	4.5	ch211-162i8.7
DMR4:44695001	4	44695001	2600	3	1.56E-10	64	2.4	TRIM14 (4 of 28)
DMR4:44702301	4	44702301	1700	3	2.18E-18	40	2.3	dkey-256i11.2
DMR4:44992201	4	44992201	4500	6	1.02E-10	63	1.4	ch211-162i8.7
DMR4:45117401	4	45117401	3500	7	7.32E-13	82	2.3	
DMR4:45259701	4	45259701	3900	4	2.57E-21	73	1.8	ch211-215p11.3
DMR4:45266201	4	45266201	4100	5	2.96E-11	115	2.8	
DMR4:45277301	4	45277301	600	2	2.68E-08	17	2.8	
DMR4:45704101	4	45704101	1700	5	2.38E-11	55	3.2	
DMR4:45955701	4	45955701	11200	8	2.42E-12	275	2.4	dkey-240b24.1
DMR4:45970001	4	45970001	2500	3	7.91E-12	91	3.6	BX470149.1
DMR4:47020701	4	47020701	1900	2	3.66E-15	38	2	

DMR4:47079901	4	47079901	3000	3	1.33E-10	105	3.5	
DMR4:47083901	4	47083901	9900	6	3.27E-20	278	2.8	
DMR4:47163001	4	47163001	21300	5	1.66E-14	689	3.2	
DMR4:47389201	4	47389201	6800	2	2.11E-08	185	2.7	
DMR4:47400601	4	47400601	1300	2	2.49E-10	49	3.7	
DMR4:47403401	4	47403401	8300	5	1.60E-10	184	2.2	
DMR4:47413001	4	47413001	1200	3	7.85E-13	25	2	
DMR4:47448201	4	47448201	1100	2	8.34E-15	30	2.7	ch211-42i6.2
DMR4:47454401	4	47454401	5100	38	2.08E-27	258	5	ch211-42i6.2
DMR4:47481601	4	47481601	2200	8	6.98E-20	35	1.5	
DMR4:47736101	4	47736101	3600	2	1.15E-08	36	1	NA
DMR4:47792001	4	47792001	14600	7	3.11E-12	358	2.4	ch211-197e7.3
DMR4:47860901	4	47860901	12400	18	2.44E-23	420	3.3	ch211-197e7.2
DMR4:47942501	4	47942501	3500	5	4.16E-12	109	3.1	
DMR4:47963601	4	47963601	8300	41	3.55E-30	193	2.3	ch211-197e7.1
DMR4:48007701	4	48007701	12700	31	3.36E-16	402	3.1	RNH1 (7 of 55)
DMR4:48028101	4	48028101	23100	50	6.40E-31	699	3	RNH1 (7 of 55)
DMR4:48052301	4	48052301	2800	3	1.56E-09	60	2.1	RNH1 (7 of 55)
DMR4:48060201	4	48060201	2200	5	1.06E-21	53	2.4	RNH1 (7 of 55)
DMR4:48072401	4	48072401	1200	8	1.31E-27	39	3.2	RNH1 (7 of 55)
DMR4:48075001	4	48075001	8100	4	5.00E-13	278	3.4	RNH1 (7 of 55)
DMR4:48095701	4	48095701	2800	3	1.12E-11	80	2.8	RNH1 (7 of 55)
DMR4:48103601	4	48103601	5100	5	2.48E-16	158	3	RNH1 (7 of 55)
DMR4:48173901	4	48173901	11300	12	1.81E-24	245	2.1	
DMR4:48201001	4	48201001	3000	6	1.67E-20	129	4.3	
DMR4:48244901	4	48244901	2000	2	1.69E-09	60	3	
DMR4:48425601	4	48425601	7400	2	1.77E-12	163	2.2	173619
DMR4:48454401	4	48454401	7000	3	4.29E-09	350	5	173619;dkey-16p6.1
DMR4:48593601	4	48593601	1400	2	7.45E-09	36	2.5	173619
DMR4:48788101	4	48788101	10000	5	5.34E-12	247	2.4	
DMR4:48800401	4	48800401	3400	3	6.99E-12	68	2	
DMR4:48863901	4	48863901	7400	22	4.99E-26	284	3.8	
DMR4:48872801	4	48872801	4200	11	2.47E-21	88	2	
DMR4:48976901	4	48976901	4800	3	3.35E-17	110	2.2	
DMR4:49060001	4	49060001	4700	5	2.80E-14	168	3.5	
DMR4:49117801	4	49117801	1700	3	2.35E-12	60	3.5	
DMR4:49294001	4	49294001	900	4	2.84E-15	35	3.8	
DMR4:49296501	4	49296501	1800	2	1.06E-21	85	4.7	
DMR4:49333101	4	49333101	5200	9	1.37E-16	76	1.4	dkey-82i20.3;
DMR4:49361201	4	49361201	2200	4	4.75E-10	106	4.8	dkey-82i20.3;
DMR4:49379001	4	49379001	5100	4	8.10E-14	98	1.9	dkey-82i20.3;
DMR4:49421301	4	49421301	7800	3	2.35E-10	178	2.2	dkey-82i20.3
DMR4:49436801	4	49436801	4900	4	4.43E-09	111	2.2	NA
DMR4:50122001	4	50122001	3300	3	1.20E-11	62	1.8	dkey-22a18.1
DMR4:50542601	4	50542601	11100	3	3.14E-11	331	2.9	
DMR4:51339401	4	51339401	2500	9	4.32E-23	138	5.5	dkey-250k10.1
DMR4:51347901	4	51347901	1100	5	2.69E-16	24	2.1	dkey-250k10.1
DMR4:51353101	4	51353101	3800	5	1.61E-14	108	2.8	dkey-250k10.1
DMR4:52846601	4	52846601	1600	3	1.55E-13	38	2.3	
DMR4:52939601	4	52939601	6400	13	3.01E-16	262	4	dkey-56m15.9
DMR4:52956401	4	52956401	2400	7	1.52E-16	55	2.2	dkey-56m15.9

DMR4:53449201	4	53449201	3100	3	3.46E-10	99	3.1	dkey-257e4.2
DMR4:53590201	4	53590201	2900	2	6.74E-15	87	3	ZFP28 (17 of 19)
DMR4:53870001	4	53870001	4100	2	3.99E-08	274	6.6	
DMR4:54485701	4	54485701	900	2	6.92E-08	38	4.2	ch211-237a4.2
DMR4:57066201	4	57066201	3600	3	6.39E-20	143	3.9	ch211-233h3.1
DMR4:57094301	4	57094301	7600	4	3.32E-14	199	2.6	ch211-233h3.1
DMR4:57106001	4	57106001	2000	2	2.29E-16	34	1.7	ch211-233h3.1
DMR4:57109501	4	57109501	6600	8	1.29E-10	141	2.1	
DMR4:57147501	4	57147501	5000	3	6.79E-16	103	2	RNH1 (4 of 55)
DMR4:57230701	4	57230701	6400	2	2.67E-09	108	1.6	ch211-241n15.1
DMR4:57308501	4	57308501	6600	24	9.44E-48	82	1.2	ch211-241n15.1
DMR4:57412801	4	57412801	3000	3	1.22E-11	72	2.4	
DMR4:57417301	4	57417301	5200	5	1.09E-11	146	2.8	
DMR4:57424301	4	57424301	5400	2	3.73E-11	156	2.8	
DMR4:57453101	4	57453101	1300	2	3.03E-09	45	3.4	dkey-149m13.2
DMR4:57462301	4	57462301	400	2	4.32E-11	13	3.2	
DMR4:57497101	4	57497101	8000	8	8.46E-26	270	3.3	
DMR4:57506101	4	57506101	5100	2	6.48E-16	102	2	
DMR4:57514901	4	57514901	3100	4	1.13E-12	55	1.7	
DMR4:57555201	4	57555201	42500	10	1.17E-09	1582	3.7	
DMR4:57635201	4	57635201	4800	9	6.02E-12	135	2.8	173710
DMR4:57694501	4	57694501	6800	4	9.14E-12	220	3.2	
DMR4:57720201	4	57720201	3200	5	2.82E-28	65	2	
DMR4:57782601	4	57782601	3300	13	7.85E-21	54	1.6	dkey-196n19.2
DMR4:57871901	4	57871901	1300	2	5.73E-11	1	0	
DMR4:57928701	4	57928701	3600	3	1.39E-22	113	3.1	ch211-178j18.2
DMR4:57989001	4	57989001	3400	8	9.85E-22	51	1.5	
DMR4:58020001	4	58020001	1700	4	1.15E-17	70	4.1	ch211-178j18.4
DMR4:58047101	4	58047101	4900	10	4.18E-36	158	3.2	U1
DMR4:58063601	4	58063601	18100	7	5.14E-14	1142	6.3	U1
DMR4:58082901	4	58082901	800	2	3.61E-08	33	4.1	
DMR4:58271901	4	58271901	6300	3	8.00E-09	226	3.5	BX465848.1
DMR4:58323301	4	58323301	2100	2	4.60E-09	32	1.5	dkey-248e17.7
DMR4:58451201	4	58451201	4500	5	1.62E-11	183	4	
DMR4:58493101	4	58493101	3800	2	2.81E-08	95	2.5	
DMR4:58501801	4	58501801	7600	11	4.06E-13	265	3.4	
DMR4:58538501	4	58538501	4300	3	1.62E-13	120	2.7	
DMR4:58637101	4	58637101	1200	2	2.29E-09	24	2	dkey-211i20.5
DMR4:58654201	4	58654201	3100	5	3.42E-11	80	2.5	dkey-211i20.2
DMR4:58668001	4	58668001	3600	6	8.93E-15	98	2.7	dkey-211i20.2
DMR4:58705601	4	58705601	900	3	5.33E-14	32	3.5	dkey-211i20.2
DMR4:58720601	4	58720601	3400	8	3.34E-12	91	2.6	dkey-211i20.2
DMR4:58753201	4	58753201	4200	2	2.86E-09	111	2.6	
DMR4:58758601	4	58758601	3800	10	3.20E-13	91	2.3	
DMR4:58764601	4	58764601	10500	31	5.60E-25	353	3.3	
DMR4:58786201	4	58786201	6700	11	6.57E-12	122	1.8	
DMR4:58888201	4	58888201	3400	4	6.38E-11	113	3.3	dkey-204l2.3
DMR4:58954601	4	58954601	3300	2	1.64E-08	73	2.2	dkey-204l2.3
DMR4:58967201	4	58967201	1500	6	9.93E-13	57	3.8	dkey-204l2.3
DMR4:58969801	4	58969801	3200	9	3.96E-11	54	1.6	dkey-204l2.3
DMR4:58999501	4	58999501	3000	3	5.15E-11	82	2.7	

DMR4:59007101	4	59007101	2100	3	5.28E-12	38	1.8	
DMR4:59125101	4	59125101	15400	8	1.09E-12	575	3.7	dkey-9p20.9
DMR4:59154001	4	59154001	2200	5	7.00E-15	41	1.8	
DMR4:59209101	4	59209101	2600	2	1.18E-09	67	2.5	
DMR4:59408801	4	59408801	2300	6	1.30E-16	57	2.4	
DMR4:59554401	4	59554401	36100	18	2.73E-11	2230	6.1	U1
DMR4:59613801	4	59613801	1600	3	6.30E-10	60	3.7	
DMR4:59695601	4	59695601	4400	5	3.28E-10	123	2.7	
DMR4:59779301	4	59779301	2200	2	1.40E-10	50	2.2	dkey-199m13.1
DMR4:59861601	4	59861601	9000	4	3.23E-11	305	3.3	
DMR4:59905301	4	59905301	8700	6	9.96E-17	190	2.1	dkey-146c18.5
DMR4:59925901	4	59925901	4800	4	2.28E-12	127	2.6	RNH1 (37 of 55)
DMR4:59955601	4	59955601	11600	11	2.56E-13	411	3.5	dkey-146c18.5
DMR4:63181501	4	63181501	1100	2	5.07E-10	29	2.6	dkey-9i5.5
DMR4:63231501	4	63231501	4600	4	3.06E-11	146	3.1	
DMR4:63260201	4	63260201	11100	3	1.41E-08	414	3.7	
DMR4:63302501	4	63302501	5200	13	3.11E-22	128	2.4	
DMR4:63477901	4	63477901	400	2	3.26E-16	3	0.7	ZFP28 (8 of 19)
DMR4:64608901	4	64608901	1000	4	4.28E-11	45	4.5	174311
DMR4:64644301	4	64644301	3300	2	2.52E-09	61	1.8	174311
DMR4:66478801	4	66478801	3100	7	2.37E-13	82	2.6	dkey-237g15.2
DMR4:66661501	4	66661501	7200	2	2.12E-15	293	4	
DMR4:66805101	4	66805101	2400	2	2.28E-14	64	2.6	dkey-264f17.1
DMR4:67433401	4	67433401	25600	37	1.88E-15	1616	6.3	U1
DMR4:67491001	4	67491001	2200	7	3.38E-11	49	2.2	ch211-120c15.2
DMR4:67494501	4	67494501	700	2	5.84E-08	15	2.1	ch211-120c15.2
DMR4:67506201	4	67506201	8100	3	6.20E-09	304	3.7	ch211-120c15.2
DMR4:67527001	4	67527001	4000	2	9.12E-18	49	1.2	dkey-246j6.2
DMR4:67553101	4	67553101	6100	5	7.78E-14	130	2.1	dkey-246j6.2
DMR4:67560501	4	67560501	5900	3	7.34E-11	126	2.1	dkey-246j6.2
DMR4:67567601	4	67567601	700	2	3.19E-09	18	2.5	ch211-120c15.3
DMR4:67615701	4	67615701	2000	2	3.02E-10	66	3.3	ch211-120c15.2
DMR4:67669801	4	67669801	11400	10	6.82E-12	288	2.5	
DMR4:67696201	4	67696201	3300	11	2.58E-22	61	1.8	174944
DMR4:67751901	4	67751901	10900	20	7.04E-27	228	2	dkey-238o14.6
DMR4:67780901	4	67780901	4500	4	4.83E-14	99	2.2	
DMR4:67804401	4	67804401	1700	2	6.40E-11	34	2	
DMR4:67828601	4	67828601	13900	7	1.34E-10	375	2.6	dkey-238o14.9
DMR4:67874101	4	67874101	8400	11	1.55E-21	139	1.6	
DMR4:67928701	4	67928701	54500	89	1.98E-17	3357	6.1	U1
DMR4:68042201	4	68042201	4400	3	1.18E-13	114	2.5	dkey-3h2.4
DMR4:68086601	4	68086601	10100	3	2.45E-10	440	4.3	dkey-3h2.4
DMR4:68116701	4	68116701	1000	2	4.64E-10	27	2.7	dkey-3h2.4
DMR4:68128701	4	68128701	4600	4	3.62E-12	103	2.2	dkey-3h2.4
DMR4:68175701	4	68175701	3900	6	3.57E-11	92	2.3	dkey-3h2.4
DMR4:68185601	4	68185601	3200	6	2.32E-13	85	2.6	dkey-3h2.4
DMR4:68210601	4	68210601	3000	2	3.59E-11	129	4.3	dkey-3h2.4
DMR4:68260701	4	68260701	10800	4	8.08E-13	503	4.6	dkey-3h2.4
DMR4:68306001	4	68306001	5100	2	6.59E-11	88	1.7	dkey-29j8.1
DMR4:68326801	4	68326801	9200	7	5.74E-14	193	2	TRIM14 (24 of 28)
DMR4:68367101	4	68367101	13200	7	8.42E-14	586	4.4	

DMR4:68702301	4	68702301	1500	2	1.18E-08	33	2.2	dkey-28k24.2
DMR4:68704801	4	68704801	2800	3	4.22E-09	65	2.3	
DMR4:68760201	4	68760201	900	2	6.48E-10	25	2.7	
DMR4:68804101	4	68804101	6200	2	9.05E-09	220	3.5	dkey-254e13.6
DMR4:69312801	4	69312801	10300	5	1.27E-15	449	4.3	ch211-76m11.3
DMR4:69367501	4	69367501	1100	2	3.41E-10	32	2.9	ch211-76m11.8
DMR4:69450801	4	69450801	1200	7	3.93E-19	31	2.5	ch211-76m11.5
DMR4:69458101	4	69458101	2700	6	6.23E-10	44	1.6	ch211-76m11.5
DMR4:69461901	4	69461901	3600	5	1.28E-23	68	1.8	ch211-76m11.5
DMR4:69498101	4	69498101	1000	4	5.23E-11	28	2.8	ch211-76m11.5
DMR4:69560401	4	69560401	3500	3	6.48E-16	90	2.5	dkey-27n6.4
DMR4:69675101	4	69675101	2400	5	9.56E-37	59	2.4	dkeyp-4f2.1
DMR4:71674701	4	71674701	5100	2	1.75E-12	174	3.4	162958
DMR4:73099301	4	73099301	3200	13	4.26E-15	102	3.1	
DMR4:73126801	4	73126801	4800	7	1.49E-17	181	3.7	
DMR4:73395701	4	73395701	2700	2	2.72E-09	31	1.1	PHF21B
DMR4:74622301	4	74622301	200	2	4.76E-13	2	1	ch211-106j21.5
DMR4:76085901	4	76085901	1500	2	4.71E-16	17	1.1	
DMR4:76150501	4	76150501	2400	2	6.55E-14	174	7.2	
DMR4:76592301	4	76592301	1800	7	8.02E-15	18	1	pacsin2;terfa
DMR5:433201	5	433201	400	2	2.14E-09	2	0.5	thap1
DMR5:1172501	5	1172501	11500	7	4.83E-11	83	0.7	
DMR6:1313101	6	1313101	2700	12	3.72E-32	83	3	
DMR7:16313501	7	16313501	6100	2	5.77E-12	112	1.8	
DMR7:18169701	7	18169701	1600	3	1.31E-10	59	3.6	
DMR7:19951901	7	19951901	400	2	4.58E-28	9	2.2	CU019646.1
DMR8:52201101	8	52201101	3400	6	2.15E-21	73	2.1	tcf7l1b
DMR8:52435501	8	52435501	3500	2	4.70E-11	155	4.4	gins4
DMR8:52846001	8	52846001	1100	2	1.73E-15	21	1.9	
DMR8:53196401	8	53196401	4500	4	4.09E-09	241	5.3	cacna1db
DMR9:53496501	9	53496501	3500	2	2.25E-09	107	3	CABZ01054962.1
DMR10:6863601	10	6863601	1500	2	1.55E-11	45	3	ppip5k2
DMR10:20834601	10	20834601	600	2	1.67E-09	19	3.1	
DMR10:23174401	10	23174401	700	5	1.11E-17	27	3.8	
DMR11:27863601	11	27863601	1000	5	3.84E-14	23	2.3	ch211-220m17.6
DMR11:29151701	11	29151701	1700	2	7.58E-09	55	3.2	arhgef10la
DMR14:3439901	14	3439901	5000	4	1.54E-11	149	2.9	
DMR14:49679901	14	49679901	800	2	1.42E-09	15	1.8	
DMR14:50908501	14	50908501	300	2	4.02E-22	5	1.6	
DMR14:51233201	14	51233201	6100	3	2.17E-17	148	2.4	
DMR14:51898101	14	51898101	2800	9	5.10E-21	17	0.6	atp6v0e1
DMR15:30691901	15	30691901	1500	3	4.07E-10	51	3.4	msi2b
DMR15:34472201	15	34472201	3500	2	1.83E-10	87	2.4	
DMR15:36875901	15	36875901	600	2	7.11E-09	8	1.3	
DMR15:36993601	15	36993601	6000	3	5.00E-11	167	2.7	
DMR15:39000301	15	39000301	2200	2	2.16E-10	34	1.5	
DMR15:39669001	15	39669001	2200	6	1.02E-22	24	1	robo1
DMR15:39828301	15	39828301	1100	2	1.04E-11	18	1.6	t2gtl4a
DMR15:39906501	15	39906501	3500	3	1.20E-15	116	3.3	CR626875.1
DMR15:39990501	15	39990501	3900	2	3.19E-14	87	2.2	
DMR15:41062101	15	41062101	800	2	1.02E-09	39	4.8	

DMR15:41124001	15	41124001	2700	5	1.63E-15	52	1.9	
DMR15:43848401	15	43848401	5400	4	6.46E-12	134	2.4	
DMR15:47004301	15	47004301	3000	2	2.47E-08	51	1.7	inppl1a
DMR16:36226201	16	36226201	3800	7	2.23E-10	102	2.6	
DMR16:36249101	16	36249101	29500	8	7.36E-10	672	2.2	
DMR16:36279601	16	36279601	4500	2	1.90E-09	127	2.8	
DMR16:36287601	16	36287601	20600	6	2.05E-10	350	1.6	
DMR16:36310901	16	36310901	5400	4	2.52E-09	149	2.7	
DMR16:36317601	16	36317601	14500	6	3.26E-10	294	2	
DMR16:36340501	16	36340501	14400	11	7.96E-11	352	2.4	
DMR16:36373201	16	36373201	4600	6	8.21E-11	80	1.7	
DMR16:36379601	16	36379601	33700	9	3.76E-10	783	2.3	CR974440.1
DMR16:36418601	16	36418601	12100	2	3.85E-08	245	2	
DMR16:36431701	16	36431701	15700	19	2.48E-11	327	2	
DMR16:36645401	16	36645401	2200	2	9.02E-09	53	2.4	
DMR17:50811801	17	50811801	1000	2	8.12E-12	32	3.2	
DMR17:50915701	17	50915701	4200	7	2.46E-19	130	3	
DMR17:50945101	17	50945101	2000	5	1.39E-12	68	3.4	aqr
DMR17:51062001	17	51062001	1000	3	1.05E-40	61	6.1	paplna
DMR17:51144601	17	51144601	500	2	3.65E-13	23	4.6	trappc12
DMR20:5911101	20	5911101	1200	4	5.50E-31	28	2.3	nrxn3b
DMR21:18842201	21	18842201	1000	2	2.34E-08	38	3.8	dkey-65l23.2
DMR21:24893001	21	24893001	2100	2	1.55E-10	39	1.8	
DMR21:26600201	21	26600201	12900	2	7.33E-10	691	5.3	
DMR22:53601	22	53601	2000	10	1.11E-11	147	7.3	mrpl20
DMR22:498001	22	498001	1600	2	2.92E-10	40	2.5	
DMR22:676601	22	676601	2800	2	1.56E-19	79	2.8	arl8a
DMR22:3303901	22	3303901	2800	2	1.33E-23	125	4.4	gipc3
DMR22:3771601	22	3771601	1400	8	3.05E-37	57	4	
DMR22:5169901	22	5169901	3700	2	5.60E-10	100	2.7	
DMR22:5267001	22	5267001	1400	2	5.71E-16	36	2.5	
DMR22:31811901	22	31811901	2100	2	6.13E-12	35	1.6	
DMR22:34701701	22	34701701	5000	4	4.74E-14	150	3	CU896691.1
DMR22:35403901	22	35403901	4000	2	3.89E-10	96	2.4	HTR3C
DMR23:8283001	23	8283001	3100	2	1.24E-09	77	2.4	
DMR23:8733301	23	8733301	2000	3	1.37E-12	46	2.3	rgs19
DMR23:12841301	23	12841301	4200	3	2.12E-12	134	3.1	
DMR23:39923401	23	39923401	600	2	6.48E-14	21	3.5	ppp1r14c
DMR23:44716801	23	44716801	3100	2	2.80E-08	109	3.5	eif4e2rs1
DMR24:2446201	24	2446201	1600	9	4.36E-20	57	3.5	rreb1a
