# Supplementary material for: Mercury-induced epigenetic transgenerational inheritance of abnormal neurobehavior is correlated with sperm epimutations in zebrafish
Source: PLoS One. 2017 May 2;12(5):e0176155. doi: 10.1371/journal.pone.0176155 (PMC5413066; doi:10.1371/journal.pone.0176155)
Supplement: S4 Table — The DMR name, chromosome number, DMR start site, length in base pair (bp), number of multiple sites, minimum p-value, CpG number per sequence length, CpG density (CpG number/100 bp) and DMR gene association with the symbol listed and NA indicating not applicable with gene but no name. (PDF) [file pone.0176155.s007.pdf]

Supplemental Table S4 F2 Generation DMR List

DMR Name	Chr	Start	Length (bp)	# Signature Windows	min P Value	CpG #	CpG Density (#/100bp)	Gene Association
DMR1:50801	1	50801	9400	3	4.84E-14	227	2.4	
DMR1:138101	1	138101	12400	4	3.79E-14	311	2.5	f10;PROZ (2 of 2);f7i
DMR1:1820401	1	1820401	1600	3	6.23E-10	44	2.7	PTGFRN (1 of 2)
DMR1:3489501	1	3489501	2900	2	1.82E-09	105	3.6	
DMR1:20390501	1	20390501	3900	7	3.72E-23	166	4.2	
DMR1:30600701	1	30600701	1900	2	2.76E-16	55	2.8	rims1b
DMR1:30614301	1	30614301	7800	15	3.00E-15	287	3.6	rims1b
DMR1:30638601	1	30638601	3100	3	5.13E-13	91	2.9	rims1b
DMR1:31510501	1	31510501	4100	2	3.06E-10	54	1.3	
DMR1:31529201	1	31529201	800	2	3.36E-08	11	1.3	
DMR1:44449201	1	44449201	5300	2	3.31E-11	74	1.3	mucms1
DMR1:47308001	1	47308001	600	2	1.68E-20	18	3	cfap58
DMR1:48885801	1	48885801	900	3	2.19E-14	22	2.4	ch211-281g13.5
DMR1:51030101	1	51030101	1300	6	2.99E-19	45	3.4	
DMR1:54091401	1	54091401	2000	3	9.13E-10	73	3.6	136791
DMR1:54259701	1	54259701	3800	5	1.81E-14	70	1.8	crtac1a
DMR1:54393201	1	54393201	3600	3	1.51E-13	19	0.5	
DMR1:54466201	1	54466201	3200	4	3.03E-11	88	2.7	mb
DMR1:54529001	1	54529001	1300	2	1.41E-11	40	3	mri1
DMR1:54692701	1	54692701	4800	3	5.92E-11	100	2	
DMR1:54750201	1	54750201	11600	4	3.05E-11	132	1	SIGLEC1 (24 of 51)
DMR1:54788101	1	54788101	2100	3	7.14E-13	40	1.9	SIGLEC1 (51 of 51)
DMR1:54948301	1	54948301	3900	2	2.46E-11	79	2	
DMR1:54966901	1	54966901	17400	3	1.04E-12	417	2.3	dkey-9c18.12
DMR1:55824201	1	55824201	1800	2	4.52E-10	33	1.8	
DMR1:55989101	1	55989101	3100	2	3.53E-09	62	2	CABZ01059415.2
DMR1:57179801	1	57179801	5000	2	6.55E-13	99	1.9	
DMR1:57335801	1	57335801	3600	4	8.54E-10	159	4.4	ch211-114l13.4
DMR1:57340601	1	57340601	700	2	2.98E-09	9	1.2	ch211-114l13.4
DMR1:57373101	1	57373101	5200	2	4.48E-08	118	2.2	caspbl
DMR1:58083101	1	58083101	3900	6	4.09E-15	331	8.4	
DMR2:1532301	2	1532301	3700	3	6.36E-15	49	1.3	adgrl2b.1
DMR2:2001801	2	2001801	700	3	1.43E-11	18	2.5	pth1ra
DMR2:4909301	2	4909301	4500	4	2.29E-11	110	2.4	tnk2b
DMR2:4915001	2	4915001	800	4	6.65E-15	30	3.7	tnk2b
DMR2:5487101	2	5487101	4200	6	2.77E-11	226	5.3	DUSP18
DMR2:6025601	2	6025601	2200	9	2.96E-12	159	7.2	tmem125b
DMR2:6174001	2	6174001	4800	2	3.35E-11	92	1.9	aldh9a1b
DMR2:6287501	2	6287501	3700	2	1.15E-09	83	2.2	
DMR2:6351201	2	6351201	2000	5	2.80E-12	71	3.5	
DMR2:6392701	2	6392701	2300	4	4.29E-16	67	2.9	otol1a
DMR2:6417001	2	6417001	11000	4	2.31E-11	284	2.5	SMC6
DMR2:6583401	2	6583401	11500	5	2.27E-16	277	2.4	
DMR2:6612801	2	6612801	2900	2	9.85E-12	55	1.8	
DMR2:8129401	2	8129401	1100	2	5.65E-09	38	3.4	ephb3a
DMR2:8318901	2	8318901	1400	2	1.72E-10	26	1.8	
DMR2:8439601	2	8439601	900	2	2.58E-13	23	2.5	
DMR2:10654201	2	10654201	1000	2	1.80E-10	21	2.1	scinlb
DMR2:11311101	2	11311101	900	3	8.42E-11	37	4.1	lrrc53
DMR2:17892701	2	17892701	4900	2	4.15E-13	93	1.8	
DMR2:18351301	2	18351301	4600	2	9.29E-11	117	2.5	

DMR2:18578601	2	18578601	4400	2	1.09E-09	74	1.6	
DMR2:20001201	2	20001201	1500	4	1.30E-12	40	2.6	
DMR2:34632601	2	34632601	400	2	1.50E-11	16	4	astn1
DMR2:37190101	2	37190101	500	2	1.67E-09	23	4.6	
DMR2:44005601	2	44005601	2200	2	9.95E-10	121	5.5	nlrb5
DMR2:47250001	2	47250001	3500	2	3.21E-08	78	2.2	
DMR2:53948501	2	53948501	2200	2	1.46E-08	24	1	
DMR2:55744801	2	55744801	2300	2	7.63E-11	60	2.6	
DMR2:56637701	2	56637701	4300	2	2.41E-08	113	2.6	CABZ01024426.1
DMR2:57658801	2	57658801	7600	3	2.63E-12	327	4.3	
DMR2:58650901	2	58650901	1800	2	1.94E-09	51	2.8	
DMR2:58874601	2	58874601	5200	2	3.54E-09	248	4.7	PTPRM
DMR3:247801	3	247801	17100	3	3.90E-10	557	3.2	BX004816.2
DMR3:289401	3	289401	4900	2	1.60E-08	151	3	
DMR3:364001	3	364001	7200	2	3.27E-10	291	4	dkey-30g5.2
DMR3:391101	3	391101	6000	5	2.43E-09	187	3.1	
DMR3:398501	3	398501	8600	11	9.23E-14	125	1.4	BX004816.4
DMR3:2308001	3	2308001	2100	3	7.35E-12	38	1.8	ch211-254c8.1
DMR3:3143601	3	3143601	1200	2	1.20E-09	34	2.8	
DMR3:3147701	3	3147701	6500	7	2.03E-12	237	3.6	
DMR3:3162801	3	3162801	9100	9	1.06E-11	199	2.1	
DMR3:3178901	3	3178901	1800	2	1.52E-09	66	3.6	
DMR3:3188401	3	3188401	11700	4	9.55E-11	350	2.9	
DMR3:3240901	3	3240901	1800	2	8.86E-11	32	1.7	
DMR3:3324801	3	3324801	7400	2	1.22E-08	123	1.6	CR388047.1
DMR3:4201201	3	4201201	8300	3	8.33E-13	189	2.2	dkey-36h5.1
DMR3:4742901	3	4742901	4000	2	4.69E-08	90	2.2	slc25a38a
DMR3:5771201	3	5771201	2200	2	9.95E-10	49	2.2	
DMR3:5784601	3	5784601	5600	2	1.08E-12	188	3.3	5S_rRNA
DMR3:5802301	3	5802301	4300	4	8.21E-11	193	4.4	TGFBR3L
DMR3:5878601	3	5878601	1200	3	2.07E-19	78	6.5	pkn1b
DMR3:5887101	3	5887101	7200	10	1.04E-24	240	3.3	pkn1b
DMR3:5897901	3	5897901	800	2	6.99E-24	34	4.2	pkn1b
DMR3:5947101	3	5947101	7700	5	1.85E-15	226	2.9	
DMR3:6007601	3	6007601	3300	2	5.04E-11	101	3	
DMR3:6323901	3	6323901	1300	3	4.47E-11	39	3	ch73-144l3.2
DMR3:6469201	3	6469201	3300	2	5.07E-08	74	2.2	ch211-12p12.4
DMR3:6877601	3	6877601	3100	2	2.34E-10	42	1.3	mast1b
DMR3:7581301	3	7581301	4400	3	2.58E-16	114	2.5	174234
DMR3:7925701	3	7925701	5100	10	2.29E-17	138	2.7	hook2
DMR3:7952001	3	7952001	1300	3	9.57E-13	44	3.3	
DMR3:7959301	3	7959301	8300	3	9.25E-11	295	3.5	ubn2b
DMR3:8006101	3	8006101	1800	2	7.88E-08	51	2.8	ubn2b
DMR3:8026501	3	8026501	13400	5	3.80E-11	477	3.5	trim35-25
DMR3:8048501	3	8048501	5600	4	9.49E-11	133	2.3	trim35-25
DMR3:8063401	3	8063401	6100	9	3.05E-18	134	2.11	trim35-23
DMR3:8076201	3	8076201	8400	4	1.92E-10	155	1.8	trim35-22
DMR3:8086001	3	8086001	8800	5	1.94E-10	211	2.3	trim35-22
DMR3:8100101	3	8100101	3400	3	8.62E-12	92	2.7	TRIM35 (28 of 38)
DMR3:8104701	3	8104701	9800	6	8.31E-14	128	1.3	TRIM35 (28 of 38)
DMR3:8115901	3	8115901	5100	2	2.05E-09	150	2.9	TRIM35 (26 of 38)
DMR3:8126701	3	8126701	10900	5	6.55E-12	466	4.2	TRIM35 (26 of 38)
DMR3:8209101	3	8209101	6600	3	8.20E-13	111	1.6	
DMR3:8271901	3	8271901	6300	2	2.24E-10	228	3.6	TRIM35 (9 of 38)
DMR3:8287501	3	8287501	8900	2	9.93E-17	238	2.6	TRIM35 (9 of 38)

DMR3:8418501	3	8418501	5900	3	6.94E-16	182	3	ch73-322f21.2
DMR3:10293501	3	10293501	2200	3	9.20E-11	21	0.9	
DMR3:12268901	3	12268901	3000	4	1.53E-20	29	0.9	
DMR3:12395201	3	12395201	1500	2	1.74E-10	11	0.7	
DMR3:12500901	3	12500901	1600	3	2.05E-12	53	3.3	abca3b
DMR3:13572001	3	13572001	7200	12	1.01E-42	102	1.4	
DMR3:13606001	3	13606001	7000	5	5.87E-14	107	1.5	
DMR3:13639201	3	13639201	3300	6	5.12E-15	120	3.6	
DMR3:13975401	3	13975401	1900	2	3.41E-14	38	2	
DMR3:14007701	3	14007701	2600	3	1.72E-25	63	2.4	ch211-108d22.2
DMR3:14011501	3	14011501	1400	5	6.70E-13	48	3.4	ch211-108d22.2
DMR3:14015501	3	14015501	3800	5	2.58E-36	184	4.8	ch211-108d22.2
DMR3:14081101	3	14081101	17600	18	4.52E-20	603	3.4	5S_rRNA
DMR3:14111101	3	14111101	3600	3	1.64E-12	95	2.6	
DMR3:14143001	3	14143001	1700	2	2.60E-13	61	3.5	
DMR3:14175401	3	14175401	2800	3	5.31E-11	32	1.1	lppr2a
DMR3:14217601	3	14217601	2600	5	5.87E-33	56	2.1	lppr2a
DMR3:14257601	3	14257601	5300	3	6.85E-14	131	2.4	tmem56b
DMR3:16535601	3	16535601	2200	2	8.16E-10	67	3	
DMR3:17045901	3	17045901	1100	2	2.44E-11	16	1.4	
DMR3:21315301	3	21315301	3400	2	1.03E-11	37	1	ch73-54a8.2
DMR3:28391001	3	28391001	500	2	4.17E-12	18	3.6	12-Sep
DMR3:28587101	3	28587101	1700	2	1.22E-12	16	0.9	gsg1l
DMR3:30394101	3	30394101	5500	3	1.70E-09	124	2.2	syt3
DMR3:30941901	3	30941901	1400	2	1.39E-17	60	4.2	tceb2
DMR3:31137001	3	31137001	1300	5	1.73E-10	38	2.9	
DMR3:31306901	3	31306901	1700	2	1.34E-12	17	1	
DMR3:31850101	3	31850101	800	2	1.06E-11	16	2	kcnc3a
DMR3:59892001	3	59892001	1900	2	1.85E-10	94	4.9	xylt2
DMR4:1530701	4	1530701	2600	2	1.80E-11	82	3.1	
DMR4:3494601	4	3494601	1700	2	1.33E-08	8	0.4	grm8a
DMR4:4873301	4	4873301	1000	5	3.05E-11	43	4.3	ptprz1b
DMR4:7031501	4	7031501	2200	3	3.71E-10	28	1.2	
DMR4:9689801	4	9689801	1500	3	1.26E-13	8	0.5	
DMR4:29125001	4	29125001	7400	8	6.84E-21	246	3.3	fb11h05
DMR4:29687601	4	29687601	9100	14	2.22E-17	120	1.3	BX248122.1
DMR4:30277801	4	30277801	2300	3	9.48E-11	56	2.4	RNH1 (6 of 55)
DMR4:33105801	4	33105801	3600	2	1.95E-08	161	4.4	
DMR4:39324201	4	39324201	4700	4	2.79E-10	109	2.3	RNH1 (45 of 55)
DMR4:40592001	4	40592001	4300	8	1.25E-19	88	2	
DMR4:44703501	4	44703501	700	2	5.16E-13	20	2.8	dkey-256i11.2
DMR4:51339201	4	51339201	2800	3	6.93E-11	144	5.1	dkey-250k10.1
DMR4:51348201	4	51348201	700	2	4.42E-10	16	2.2	dkey-250k10.1
DMR4:51909501	4	51909501	3300	2	1.72E-08	105	3.1	
DMR4:52940101	4	52940101	6200	17	9.71E-19	258	4.1	dkey-56m15.9
DMR4:52956001	4	52956001	2900	9	8.77E-27	64	2.2	dkey-56m15.9
DMR4:53883901	4	53883901	7500	3	3.51E-08	292	3.8	
DMR4:63845601	4	63845601	2400	2	1.51E-11	55	2.2	
DMR4:67286701	4	67286701	5200	5	7.51E-19	86	1.6	ch211-209j12.4
DMR4:69395401	4	69395401	3300	3	9.10E-09	58	1.7	ch211-76m11.3
DMR4:70554901	4	70554901	2200	3	1.68E-16	107	4.8	
DMR4:70713201	4	70713201	1900	2	9.72E-13	40	2.1	ftr64
DMR4:70945701	4	70945701	6400	4	6.57E-09	176	2.7	CABZ01054394.4
DMR4:72017901	4	72017901	2400	8	1.62E-20	73	3	
DMR4:72074701	4	72074701	3400	7	1.19E-13	123	3.6	BX855614.4

DMR4:72107201	4	72107201	6500	7	8.44E-14	552	8.4	171551
DMR4:72489901	4	72489901	4400	2	3.62E-10	147	3.3	
DMR4:72738501	4	72738501	2500	2	8.41E-13	168	6.7	
DMR4:72819201	4	72819201	8300	3	1.73E-11	306	3.6	ptprb
DMR4:72841601	4	72841601	2200	3	7.08E-13	119	5.4	ptprb
DMR4:72895901	4	72895901	2600	13	1.17E-23	137	5.2	ptprb
DMR4:73006401	4	73006401	5600	3	2.75E-09	188	3.3	
DMR4:73033001	4	73033001	8500	3	9.47E-12	304	3.5	
DMR4:73098601	4	73098601	3900	17	4.62E-20	116	2.9	
DMR4:74120701	4	74120701	1400	2	2.34E-11	12	0.8	dkey-261j11.2
DMR4:74129901	4	74129901	8700	3	1.29E-15	403	4.6	dkey-261j11.2
DMR4:74143101	4	74143101	1500	3	1.83E-18	25	1.6	dkey-261j11.2
DMR4:75006601	4	75006601	1200	2	6.20E-14	43	3.5	ms4a17a.11
DMR4:75342001	4	75342001	1600	4	5.53E-13	47	2.9	NA
DMR4:75579501	4	75579501	1800	3	4.47E-12	133	7.3	CU467646.4
DMR4:76173401	4	76173401	3800	2	3.62E-12	82	2.1	
DMR5:2396701	5	2396701	2100	3	7.98E-12	26	1.2	
DMR5:3638301	5	3638301	1100	2	1.87E-10	15	1.3	MYO19
DMR5:3967401	5	3967401	23700	4	1.19E-08	648	2.7	prdx4
DMR5:4188901	5	4188901	5300	4	1.69E-14	81	1.5	PLA2G4C (3 of 4)
DMR5:4250601	5	4250601	8400	13	1.32E-13	154	1.8	FO834898.1
DMR5:4281001	5	4281001	3000	3	1.10E-11	44	1.4	FO834898.1
DMR5:4291501	5	4291501	2700	4	2.16E-13	88	3.2	FO834898.1
DMR5:4347901	5	4347901	2900	2	2.39E-14	81	2.7	
DMR5:4394001	5	4394001	10200	6	4.93E-13	169	1.6	angptl2a
DMR5:4405201	5	4405201	1800	4	2.33E-12	45	2.5	
DMR5:4432001	5	4432001	2200	3	8.92E-22	59	2.6	
DMR5:5272901	5	5272901	2500	5	8.36E-17	22	0.8	TNC (2 of 2)
DMR5:5466301	5	5466301	9600	14	2.54E-11	138	1.4	
DMR5:5631701	5	5631701	800	2	7.58E-13	28	3.5	
DMR5:5986201	5	5986201	2000	2	2.76E-09	54	2.7	tnks1bp1
DMR5:6586901	5	6586901	900	2	2.23E-10	10	1.1	
DMR5:6640501	5	6640501	600	2	9.48E-09	31	5.1	
DMR5:6976301	5	6976301	1000	2	1.85E-12	28	2.8	UNC5C (2 of 2)
DMR5:7035501	5	7035501	500	2	4.40E-08	13	2.6	bmpr1ba
DMR5:7069901	5	7069901	4200	2	2.20E-09	109	2.5	ANGPTL2 (3 of 3);bmpr1ba
DMR5:7178001	5	7178001	4300	3	5.13E-14	62	1.4	bmpr1ba
DMR5:8967401	5	8967401	2800	4	2.31E-13	59	2.1	gak
DMR5:8971701	5	8971701	1000	2	3.19E-09	33	3.3	gak
DMR5:8995601	5	8995601	4800	3	3.11E-09	118	2.4	gak
DMR5:10214801	5	10214801	2700	3	5.33E-12	73	2.7	
DMR5:10671601	5	10671601	4300	4	1.54E-12	107	2.4	
DMR5:11084501	5	11084501	2900	2	1.38E-09	49	1.6	
DMR5:13216101	5	13216101	3000	2	6.19E-09	98	3.2	mxd1
DMR5:13667801	5	13667801	7300	8	6.26E-11	218	2.9	npffr1l2
DMR5:17311701	5	17311701	700	2	3.37E-11	23	3.2	
DMR5:19369301	5	19369301	1600	4	1.00E-14	34	2.1	tchp
DMR5:19830401	5	19830401	8500	2	1.36E-09	209	2.4	coro1ca
DMR5:34749201	5	34749201	5700	2	8.45E-12	236	4.1	
DMR5:59962201	5	59962201	1000	4	3.42E-16	17	1.7	tmem132e
DMR5:60584201	5	60584201	2300	2	2.80E-20	28	1.2	doc2b
DMR5:60588801	5	60588801	1700	2	2.89E-10	54	3.1	doc2b
DMR5:63572301	5	63572301	3900	3	1.46E-08	91	2.3	
DMR5:63864401	5	63864401	4100	6	1.81E-11	32	0.7	
DMR5:64042001	5	64042001	2000	2	3.39E-10	24	1.2	

DMR5:64095201	5	64095201	3100	2	8.80E-09	77	2.4	
DMR5:64357401	5	64357401	2400	2	1.02E-14	55	2.2	pmpca
DMR5:64364101	5	64364101	900	3	1.61E-14	43	4.7	pmpca
DMR5:64414401	5	64414401	1900	8	1.25E-40	22	1.1	
DMR5:64654101	5	64654101	900	2	9.44E-10	27	3	
DMR5:64725501	5	64725501	1900	2	1.81E-11	37	1.9	
DMR5:66964801	5	66964801	1500	3	1.46E-10	26	1.7	zbtb20
DMR5:67041601	5	67041601	11900	6	6.19E-13	245	2	arhgap31
DMR5:67061901	5	67061901	1000	3	5.01E-10	45	4.5	b4galt4
DMR5:67190401	5	67190401	600	2	3.61E-18	25	4.1	
DMR5:67246001	5	67246001	900	2	2.76E-10	36	4	
DMR5:67301801	5	67301801	800	2	2.62E-10	27	3.3	gtf3aa
DMR5:67401401	5	67401401	2400	4	1.71E-13	71	2.9	slc25a11
DMR5:67408901	5	67408901	3300	2	8.35E-20	81	2.4	slc25a11
DMR5:67983101	5	67983101	1700	9	4.47E-18	61	3.5	
DMR5:68928901	5	68928901	1000	2	3.72E-12	23	2.3	
DMR5:69209001	5	69209001	2200	2	1.28E-09	24	1	ch211-154e10.1
DMR5:69439401	5	69439401	3100	8	1.58E-12	16	0.5	
DMR5:69910101	5	69910101	3800	2	2.41E-09	29	0.7	
DMR5:70252601	5	70252601	1000	2	8.58E-12	34	3.4	
DMR5:70272501	5	70272501	600	3	2.68E-23	35	5.8	
DMR5:70285901	5	70285901	6500	5	1.22E-22	106	1.6	
DMR5:70331501	5	70331501	1100	2	1.18E-08	22	2	
DMR5:70393901	5	70393901	8200	5	1.02E-27	220	2.6	
DMR5:70428201	5	70428201	2800	3	3.52E-12	11	0.3	
DMR5:70893201	5	70893201	1700	2	3.35E-13	18	1	
DMR5:70970501	5	70970501	3600	3	9.13E-14	76	2.1	gpsm1b
DMR5:71172001	5	71172001	500	2	1.61E-10	7	1.4	nup214
DMR5:71201101	5	71201101	2800	2	2.11E-12	54	1.9	fam78ab
DMR5:71402601	5	71402601	4800	3	1.32E-21	83	1.7	
DMR5:71456001	5	71456001	5000	3	3.91E-14	162	3.2	ddx54
DMR5:71582301	5	71582301	5400	2	2.52E-16	128	2.3	ddx54
DMR6:1312501	6	1312501	3200	3	2.83E-10	90	2.8	
DMR6:9689001	6	9689001	1300	3	1.93E-08	50	3.8	
DMR6:36138801	6	36138801	900	3	1.52E-09	48	5.3	ch211-205j18.1
DMR6:37840601	6	37840601	2100	3	3.76E-10	28	1.3	herc2
DMR6:41535401	6	41535401	1100	4	1.63E-14	21	1.9	hemk1
DMR6:48370601	6	48370601	700	5	6.12E-29	20	2.8	mov10a
DMR6:50732101	6	50732101	7200	2	7.95E-09	156	2.1	
DMR6:59227701	6	59227701	3900	4	9.38E-19	122	3.1	fam210b;shmt2
DMR6:59314501	6	59314501	5800	5	2.61E-15	54	0.9	fam210b
DMR6:59329201	6	59329201	4400	3	2.14E-12	134	3	fam210b
DMR6:59339901	6	59339901	7800	3	3.37E-18	150	1.9	fam210b
DMR6:59349401	6	59349401	4900	5	1.68E-24	92	1.8	fam210b
DMR6:59740801	6	59740801	5200	7	1.44E-39	163	3.1	NUFIP1
DMR6:59951801	6	59951801	6700	27	2.00E-21	196	2.9	pmepa1;kdm6al
DMR6:60175401	6	60175401	6400	20	1.54E-56	156	2.4	
DMR6:60185901	6	60185901	1200	4	1.29E-17	97	8	
DMR6:60224101	6	60224101	2100	2	5.77E-15	53	2.5	
DMR6:60227601	6	60227601	4500	3	1.90E-15	131	2.9	
DMR6:60258501	6	60258501	2300	2	1.27E-10	53	2.3	
DMR7:55101	7	55101	1800	4	1.27E-22	37	2	
DMR7:2052101	7	2052101	9400	4	4.91E-13	254	2.7	
DMR7:3622201	7	3622201	2100	3	8.66E-12	54	2.5	ch211-282j17.11
DMR7:4188701	7	4188701	2000	2	2.00E-08	86	4.3	

DMR7:10092301	7	10092301	4600	2	3.27E-10	62	1.3	
DMR7:10141901	7	10141901	2200	2	1.26E-11	49	2.2	
DMR7:25119701	7	25119701	4200	5	2.95E-15	145	3.4	
DMR7:42584401	7	42584401	1300	2	4.70E-13	54	4.1	
DMR7:47553201	7	47553201	2900	2	1.47E-17	24	0.8	ch211-186j3.6
DMR7:51293801	7	51293801	1600	4	2.85E-18	37	2.3	
DMR7:53854501	7	53854501	1800	2	2.36E-08	39	2.1	csnk1g1
DMR7:53972301	7	53972301	1800	2	1.25E-08	42	2.3	pacsin3
DMR7:54351401	7	54351401	2700	8	8.01E-14	67	2.4	fgf4
DMR7:56172201	7	56172201	3400	4	8.53E-14	40	1.1	sult5a1;ist1
DMR7:59082601	7	59082601	2300	2	2.68E-08	79	3.4	SEMA4F
DMR7:59099201	7	59099201	3100	2	5.48E-10	53	1.7	SEMA4F
DMR7:59417801	7	59417801	7100	4	4.01E-10	259	3.6	
DMR7:60200401	7	60200401	8400	3	1.23E-09	138	1.6	pcxb
DMR7:60330301	7	60330301	5500	2	1.80E-08	102	1.8	pcxb
DMR7:60561601	7	60561601	5900	19	4.14E-12	137	2.3	
DMR7:61403301	7	61403301	1800	3	6.39E-12	31	1.7	
DMR7:62898001	7	62898001	2900	2	1.49E-13	60	2	
DMR7:62988901	7	62988901	6100	3	6.33E-19	143	2.3	
DMR7:64599101	7	64599101	1700	2	1.20E-08	81	4.7	MMP15 (1 of 2)
DMR7:66282101	7	66282101	5300	4	2.84E-20	107	2	
DMR7:66368601	7	66368601	1600	3	4.46E-10	47	2.9	
DMR8:3112301	8	3112301	3300	4	1.16E-09	59	1.7	
DMR8:26093201	8	26093201	1100	6	5.53E-20	42	3.8	
DMR8:30671801	8	30671801	2600	2	7.14E-10	100	3.8	adora2aa
DMR8:30711401	8	30711401	1700	3	3.02E-10	43	2.5	upb1
DMR8:41765001	8	41765001	1600	2	6.96E-09	30	1.8	
DMR8:44073801	8	44073801	800	3	6.10E-13	14	1.7	
DMR8:44636501	8	44636501	1700	2	1.04E-12	43	2.5	grk5l
DMR8:45429401	8	45429401	800	4	9.53E-15	25	3.1	
DMR8:46092501	8	46092501	5400	2	5.82E-14	177	3.2	mtor
DMR8:46244801	8	46244801	4700	6	1.77E-14	122	2.5	mtor
DMR8:46527701	8	46527701	8900	14	6.87E-15	240	2.6	ch211-196g2.7
DMR8:47333201	8	47333201	1200	2	2.44E-10	33	2.7	pex10
DMR8:47975401	8	47975401	4600	9	8.64E-14	131	2.8	
DMR8:48032701	8	48032701	1200	3	2.28E-13	55	4.5	
DMR8:48309801	8	48309801	700	2	1.51E-08	18	2.5	PRDM16
DMR8:48794601	8	48794601	4400	3	4.02E-11	78	1.7	ch211-278p7.5
DMR8:48902001	8	48902001	7500	6	5.78E-17	193	2.5	tp73
DMR8:48999401	8	48999401	6000	3	2.39E-13	122	2	aak1a
DMR8:49121501	8	49121501	6500	5	2.39E-09	138	2.1	GOLM1
DMR8:53196501	8	53196501	3500	3	4.30E-09	201	5.7	cacna1db
DMR9:4770701	9	4770701	1100	4	1.52E-14	3	0.2	fmnl2a
DMR9:5760001	9	5760001	4100	2	2.20E-10	146	3.5	
DMR9:8577701	9	8577701	1700	4	4.72E-10	35	2	
DMR9:11792301	9	11792301	5300	2	5.93E-10	105	1.9	
DMR9:11886401	9	11886401	3900	4	2.71E-10	115	2.9	
DMR9:11931501	9	11931501	600	2	1.00E-08	11	1.8	
DMR9:19846401	9	19846401	6700	2	1.77E-13	282	4.2	pdxka
DMR9:19854101	9	19854101	3400	4	8.67E-13	111	3.2	
DMR9:19862601	9	19862601	7400	9	1.36E-12	268	3.6	
DMR9:19903201	9	19903201	5500	2	1.33E-11	138	2.5	ch211-141e20.5
DMR9:19936801	9	19936801	5400	3	4.01E-15	121	2.2	ch211-141e20.6
DMR9:20056001	9	20056001	7400	3	7.44E-12	286	3.8	
DMR9:20078301	9	20078301	6400	2	5.30E-17	142	2.2	

DMR9:20125601	9	20125601	3500	6	1.90E-10	65	1.8	
DMR9:23333701	9	23333701	4900	2	1.00E-08	81	1.6	lypd6b
DMR9:23490701	9	23490701	6300	5	1.17E-08	349	5.5	tmem163a
DMR9:24526901	9	24526901	1800	5	7.89E-21	107	5.9	tmeff2a
DMR9:24736901	9	24736901	5700	2	8.04E-10	135	2.3	5S_rRNA
DMR9:26576901	9	26576901	2800	2	4.37E-11	36	1.2	dkey-111i23.1
DMR9:43671001	9	43671001	3100	2	4.13E-09	36	1.1	znf385b
DMR9:43901601	9	43901601	4200	2	3.10E-10	81	1.9	
DMR9:49275001	9	49275001	2400	3	2.66E-15	111	4.6	
DMR9:49914201	9	49914201	1700	2	2.79E-09	38	2.2	
DMR9:50157501	9	50157501	1800	4	7.43E-13	108	6	ttc21b
DMR9:50379601	9	50379601	6300	5	2.16E-12	195	3	COBLL1 (2 of 2)
DMR9:50763401	9	50763401	7300	13	2.33E-17	145	1.9	
DMR9:51915301	9	51915301	5100	5	1.58E-10	135	2.6	NA
DMR9:52335301	9	52335301	2000	2	1.06E-09	75	3.7	
DMR9:52799301	9	52799301	900	4	4.49E-14	3	0.3	nme8
DMR9:52892901	9	52892901	2200	2	1.36E-09	35	1.5	nme8;smarcal1
DMR9:54539001	9	54539001	3700	5	1.54E-15	74	2	frmpd4
DMR9:55317901	9	55317901	400	2	1.48E-10	8	2	
DMR9:55330401	9	55330401	2100	2	9.14E-15	37	1.7	
DMR9:55347801	9	55347801	4900	2	3.72E-18	82	1.6	glra2
DMR9:55370401	9	55370401	4500	3	1.33E-10	142	3.1	fancb
DMR9:56011201	9	56011201	7100	2	7.52E-09	75	1	edar
DMR9:56080101	9	56080101	2300	10	5.67E-14	26	1.1	
DMR10:1817601	10	1817601	5100	2	1.30E-12	169	3.3	apc
DMR10:3196401	10	3196401	800	2	2.48E-10	20	2.5	pi4kaa
DMR10:3220601	10	3220601	1300	2	5.01E-15	56	4.3	pi4kaa
DMR10:3224801	10	3224801	2900	3	5.73E-17	60	2	pi4kaa
DMR10:3261601	10	3261601	2100	3	1.88E-14	40	1.9	
DMR10:3265501	10	3265501	2000	2	3.98E-09	106	5.3	
DMR10:3286701	10	3286701	2200	3	1.02E-14	100	4.5	slc25a1b
DMR10:4190701	10	4190701	1100	4	2.01E-29	49	4.4	
DMR10:4341701	10	4341701	3500	4	6.12E-21	150	4.2	
DMR10:4381801	10	4381801	1000	2	9.58E-10	40	4	
DMR10:4441901	10	4441901	2000	3	9.31E-10	76	3.8	
DMR10:4491001	10	4491001	3700	3	1.62E-13	64	1.7	
DMR10:7154301	10	7154301	1900	2	5.12E-29	57	3	psd3l
DMR10:7280501	10	7280501	2700	2	3.47E-11	39	1.4	
DMR10:7659201	10	7659201	2900	5	1.24E-21	70	2.4	
DMR10:7682801	10	7682801	3300	4	1.30E-09	143	4.3	
DMR10:7807601	10	7807601	500	2	8.38E-10	14	2.8	
DMR10:7963401	10	7963401	5200	2	5.73E-14	112	2.1	osbp2
DMR10:8034801	10	8034801	1300	2	1.07E-08	94	7.2	atp6v0a2a
DMR10:8160001	10	8160001	3600	3	2.31E-14	76	2.1	pstpip2
DMR10:8217601	10	8217601	1800	3	7.72E-13	58	3.2	DHX29
DMR10:10460601	10	10460601	4500	5	3.46E-09	104	2.3	sardh
DMR10:10471301	10	10471301	1600	3	2.21E-12	62	3.8	sardh
DMR10:10883401	10	10883401	1400	2	6.21E-12	31	2.2	
DMR10:14266501	10	14266501	6200	4	1.19E-16	147	2.3	
DMR10:14860501	10	14860501	3600	5	4.86E-17	147	4	
DMR10:15026401	10	15026401	1200	3	6.00E-13	18	1.5	dkey-88l16.3
DMR10:15765901	10	15765901	7200	2	7.48E-10	196	2.7	
DMR10:15787501	10	15787501	13100	4	5.03E-12	321	2.4	BX629350.3
DMR10:17582401	10	17582401	3100	2	1.65E-09	68	2.1	dkey-76p7.7
DMR10:17738101	10	17738101	3900	2	7.78E-11	57	1.4	

DMR10:18544001	10	18544001	1800	2	5.75E-18	14	0.7	
DMR10:21842801	10	21842801	1400	3	8.10E-11	33	2.3	pcdh1gb2;pcdh1g3
DMR10:22833001	10	22833001	1200	2	1.53E-08	16	1.3	pcolcea
DMR10:22979201	10	22979201	1300	3	5.96E-11	17	1.3	
DMR10:23082301	10	23082301	2100	2	1.59E-11	64	3	
DMR10:23455601	10	23455601	5800	3	3.74E-10	199	3.4	
DMR10:25871301	10	25871301	600	2	5.00E-12	21	3.5	trpc4a
DMR10:26248701	10	26248701	1500	2	9.26E-09	46	3	arfip2b
DMR10:26899601	10	26899601	1200	3	1.70E-10	21	1.7	
DMR10:27192001	10	27192001	4400	4	9.34E-11	95	2.1	cxadr
DMR10:27824201	10	27824201	6700	2	3.56E-08	112	1.6	
DMR10:28006501	10	28006501	2900	4	1.24E-13	80	2.7	
DMR10:29032301	10	29032301	1500	3	1.51E-09	39	2.6	
DMR10:32594301	10	32594301	800	2	1.85E-12	1	0.1	mogat2
DMR10:32607101	10	32607101	2900	5	4.23E-11	72	2.4	mogat2
DMR10:32665001	10	32665001	700	2	1.42E-09	13	1.8	mogat2
DMR10:33225101	10	33225101	2900	4	2.24E-16	50	1.7	myl10
DMR10:34645101	10	34645101	1900	2	5.40E-09	29	1.5	nbeaa
DMR10:36032901	10	36032901	4000	6	1.93E-11	28	0.7	
DMR10:36162701	10	36162701	500	2	2.64E-09	11	2.2	
DMR10:37363101	10	37363101	1200	3	1.95E-25	36	3	nf1b
DMR10:37896601	10	37896601	1900	4	4.86E-15	30	1.5	
DMR10:38110701	10	38110701	7300	3	5.18E-09	191	2.6	
DMR10:38684701	10	38684701	5000	3	7.27E-12	187	3.7	
DMR10:38708701	10	38708701	3000	2	4.25E-09	59	1.9	mmp30
DMR10:38792201	10	38792201	1100	2	4.64E-10	15	1.3	
DMR10:39732901	10	39732901	1600	2	4.16E-09	38	2.3	kirrel3a
DMR10:40062301	10	40062301	4200	3	1.39E-10	81	1.9	CLMP
DMR10:41094801	10	41094801	700	2	8.68E-19	56	8	antxr1b
DMR10:41342901	10	41342901	5100	5	1.70E-14	135	2.6	
DMR11:1219201	11	1219201	4400	5	3.30E-14	160	3.6	atp2b2
DMR11:1321101	11	1321101	7000	25	2.83E-29	159	2.2	iars
DMR11:1486301	11	1486301	7300	2	1.16E-09	212	2.9	acot8
DMR11:1631001	11	1631001	11200	2	9.17E-09	224	2	LRP1 (2 of 2)
DMR11:1689401	11	1689401	5200	5	1.45E-13	163	3.1	LRP1 (2 of 2)
DMR11:1703701	11	1703701	6000	3	2.04E-14	147	2.4	LRP1 (2 of 2)
DMR11:1746801	11	1746801	2700	2	7.37E-11	93	3.4	LRP1 (2 of 2)
DMR11:1765501	11	1765501	4600	2	2.34E-08	126	2.7	LRP1 (2 of 2)
DMR11:16463001	11	16463001	2700	3	1.26E-10	124	4.5	
DMR11:16469601	11	16469601	1800	3	4.44E-11	90	5	
DMR11:17705801	11	17705801	2100	3	1.89E-09	95	4.5	
DMR11:37308801	11	37308801	2200	2	2.05E-10	42	1.9	
DMR11:38399201	11	38399201	2100	2	5.89E-11	77	3.6	
DMR11:38487401	11	38487401	1000	2	3.42E-09	10	1	epha8
DMR11:39766801	11	39766801	1500	2	1.65E-12	18	1.2	
DMR11:40058201	11	40058201	5100	3	3.10E-13	106	2	
DMR11:41633401	11	41633401	2700	5	5.07E-14	15	0.5	
DMR12:1351201	12	1351201	6900	3	2.26E-10	206	2.9	pemt
DMR12:7709501	12	7709501	2800	3	5.84E-13	79	2.8	ank3b
DMR12:11179701	12	11179701	20600	2	4.00E-09	582	2.8	
DMR12:34960201	12	34960201	6200	2	6.83E-09	120	1.9	112285
DMR12:37594701	12	37594701	3100	2	1.75E-10	54	1.7	
DMR13:2632001	13	2632001	2000	4	2.48E-16	57	2.8	wdr11
DMR13:2665501	13	2665501	5100	10	2.16E-27	134	2.6	wdr11
DMR13:8301301	13	8301301	2900	2	3.19E-11	143	4.9	

DMR13:10424801	13	10424801	800	3	7.41E-14	17	2.1	ch73-54n14.2
DMR13:10440101	13	10440101	5800	4	9.79E-11	129	2.2	ch73-54n14.2
DMR13:12435901	13	12435901	4700	2	2.38E-11	43	0.9	enpep
DMR13:12612301	13	12612301	2200	4	1.40E-18	27	1.2	metap1
DMR13:13109801	13	13109801	3200	2	1.02E-13	87	2.7	fgfr3
DMR13:13196901	13	13196901	1400	3	6.61E-11	48	3.4	
DMR13:13233001	13	13233001	2500	2	4.26E-09	84	3.3	
DMR13:18077601	13	18077601	5800	5	2.51E-10	240	4.1	tet1
DMR13:21354201	13	21354201	400	2	1.15E-08	3	0.7	
DMR13:22908101	13	22908101	400	2	3.79E-10	27	6.7	supv3l1
DMR13:23021701	13	23021701	2700	2	2.90E-09	90	3.3	sorbs1
DMR13:23363001	13	23363001	3900	4	8.90E-12	104	2.6	prim2
DMR13:24034601	13	24034601	2500	3	2.78E-11	53	2.1	galnt2
DMR13:26639501	13	26639501	3600	2	1.10E-20	85	2.3	fancl
DMR13:26796301	13	26796301	700	2	1.79E-08	21	3	
DMR13:27724001	13	27724001	8900	20	5.84E-12	271	3	
DMR13:27747601	13	27747601	700	4	9.66E-11	19	2.7	
DMR13:28316601	13	28316601	2500	3	1.59E-14	56	2.2	
DMR13:28435101	13	28435101	1800	2	1.58E-08	37	2	
DMR13:29142601	13	29142601	5500	5	1.41E-13	231	4.2	MYOF (2 of 2)
DMR13:48571401	13	48571401	5800	2	1.51E-08	154	2.6	
DMR14:4045801	14	4045801	400	2	1.65E-10	3	0.7	dhrs13l1
DMR14:4297001	14	4297001	1800	2	7.56E-09	50	2.7	
DMR14:4352901	14	4352901	2700	2	1.15E-08	82	3	GABRA4
DMR14:6224301	14	6224301	2100	2	8.61E-11	45	2.1	ch211-198i6.4
DMR14:6407001	14	6407001	1400	3	2.52E-10	32	2.2	COL23A1 (2 of 2)
DMR14:7286901	14	7286901	3100	4	7.28E-14	93	3	
DMR14:8610101	14	8610101	2400	4	1.09E-11	62	2.5	153681
DMR14:8613701	14	8613701	2500	11	5.70E-32	57	2.2	153681
DMR14:23562401	14	23562401	6200	4	1.47E-14	110	1.7	
DMR14:43486101	14	43486101	2100	2	8.33E-09	17	0.8	
DMR14:46143801	14	46143801	700	4	1.99E-19	20	2.8	
DMR14:47320801	14	47320801	1500	6	4.22E-22	25	1.6	crybb1l1
DMR15:4629801	15	4629801	1900	2	8.02E-11	46	2.4	
DMR15:8415401	15	8415401	700	3	2.15E-15	17	2.4	
DMR15:14568101	15	14568101	2800	2	2.20E-16	90	3.2	numbl
DMR15:15205301	15	15205301	1000	5	2.73E-13	57	5.7	
DMR15:17310401	15	17310401	1700	2	1.34E-08	50	2.9	
DMR15:17327201	15	17327201	3500	4	3.29E-17	69	1.9	dhx40
DMR15:18099401	15	18099401	1800	2	1.22E-10	40	2.2	phldb1b
DMR15:28664901	15	28664901	400	2	4.49E-10	14	3.5	
DMR15:41807601	15	41807601	900	2	1.03E-09	26	2.8	
DMR15:43849201	15	43849201	5200	2	4.30E-10	84	1.6	
DMR16:1359501	16	1359501	2800	2	7.47E-10	34	1.2	cers2b
DMR16:1744201	16	1744201	2600	4	1.04E-11	53	2	
DMR16:22010501	16	22010501	4600	8	2.05E-13	172	3.7	
DMR16:29411601	16	29411601	600	3	9.05E-09	23	3.8	
DMR16:29822701	16	29822701	500	2	1.04E-12	4	0.8	
DMR16:32433501	16	32433501	3200	4	4.79E-09	62	1.9	
DMR16:35830501	16	35830501	1700	2	2.92E-13	22	1.2	
DMR16:36368801	16	36368801	2600	2	1.20E-12	45	1.7	
DMR16:37267901	16	37267901	900	5	8.56E-20	30	3.3	TSNARE1
DMR16:38693501	16	38693501	1700	3	2.41E-09	40	2.3	
DMR16:43445101	16	43445101	4600	6	1.86E-14	104	2.2	FO704821.1;ccdc127a
DMR16:44448301	16	44448301	500	2	1.94E-08	18	3.6	sult2st3

DMR16:54373001	16	54373001	7600	2	2.84E-09	218	2.8	xrcc1
DMR16:55305301	16	55305301	1000	2	1.89E-08	35	3.5	CNDP1
DMR17:656101	17	656101	3600	3	1.06E-11	91	2.5	ch211-193k8.5;dnajc17
DMR17:730801	17	730801	1000	3	5.23E-09	48	4.8	dnajc17
DMR17:1552001	17	1552001	1100	2	2.07E-09	96	8.7	NA
DMR17:2782201	17	2782201	3100	2	3.00E-12	75	2.4	
DMR17:4691101	17	4691101	4000	4	6.15E-25	94	2.3	KLHL29 (1 of 2)
DMR17:7873501	17	7873501	1700	3	2.43E-34	75	4.4	syne1b
DMR17:8203801	17	8203801	2900	7	3.40E-18	95	3.2	cdc42bpaa
DMR17:8778001	17	8778001	1200	2	2.80E-10	48	4	psmc1a
DMR17:14505201	17	14505201	1200	4	2.14E-24	13	1	daam1a
DMR17:14772501	17	14772501	3000	2	6.27E-09	67	2.2	ch211-266o15.1
DMR17:50358601	17	50358601	5100	3	2.75E-09	138	2.7	
DMR17:51251501	17	51251501	700	2	5.25E-10	15	2.1	
DMR17:51757501	17	51757501	3900	3	5.76E-21	176	4.5	numb
DMR17:52022801	17	52022801	1400	6	2.77E-12	58	4.1	
DMR17:52115501	17	52115501	6800	2	4.71E-11	181	2.6	
DMR17:52816001	17	52816001	3300	8	3.28E-18	99	3	meis2a
DMR17:52867901	17	52867901	3800	2	3.35E-14	85	2.2	C17H15orf41
DMR17:52913101	17	52913101	3300	3	1.08E-09	112	3.3	C17H15orf41
DMR17:53131201	17	53131201	4700	4	1.58E-10	72	1.5	
DMR18:2197401	18	2197401	2300	9	4.88E-11	35	1.5	
DMR18:2941701	18	2941701	7900	4	9.23E-12	189	2.3	clns1a
DMR18:2960001	18	2960001	3100	2	1.15E-08	81	2.6	
DMR18:2971401	18	2971401	1600	2	3.15E-09	74	4.6	rsf1a
DMR18:3188401	18	3188401	1500	3	3.84E-09	54	3.6	
DMR18:3276701	18	3276701	2700	2	1.63E-09	77	2.8	pak1
DMR18:3317301	18	3317301	1600	2	4.06E-09	48	3	
DMR18:3456301	18	3456301	5000	2	8.61E-09	118	2.3	eif2a
DMR18:8796301	18	8796301	900	2	4.31E-09	17	1.8	
DMR18:11004901	18	11004901	1600	2	9.16E-11	26	1.6	ch211-59c24.1
DMR18:36671501	18	36671501	800	2	2.33E-09	30	3.7	
DMR18:38110401	18	38110401	3900	4	1.70E-11	72	1.8	
DMR18:38463601	18	38463601	500	2	5.49E-09	10	2	
DMR18:39019801	18	39019801	6500	3	1.11E-09	134	2	myo5aa
DMR18:39628101	18	39628101	3600	2	3.41E-10	72	2	
DMR18:47695101	18	47695101	700	5	9.60E-19	11	1.5	
DMR18:48025101	18	48025101	1300	3	6.61E-13	56	4.3	
DMR18:48920001	18	48920001	2000	2	1.00E-10	87	4.3	ppp1r37
DMR18:50799701	18	50799701	4300	6	3.01E-18	65	1.5	ddb1
DMR19:23601	19	23601	3800	2	1.33E-09	99	2.6	gpd1c
DMR19:1454801	19	1454801	4100	2	9.38E-09	89	2.1	
DMR19:1622701	19	1622701	800	3	2.41E-10	34	4.2	
DMR19:17841801	19	17841801	1500	3	1.77E-09	107	7.1	
DMR19:35537801	19	35537801	800	4	4.22E-11	27	3.3	macf1a
DMR19:48419401	19	48419401	2400	2	6.48E-08	64	2.6	psmd3;btr30
DMR20:3615101	20	3615101	1100	2	3.23E-08	41	3.7	CABZ01071723.1
DMR20:4992601	20	4992601	4300	4	1.45E-09	133	3	arid1b
DMR20:5558801	20	5558801	3000	4	1.15E-12	50	1.6	nrxn3b
DMR20:9384301	20	9384301	2200	3	5.68E-18	40	1.8	BEGAIN
DMR20:15628101	20	15628101	500	3	1.12E-14	17	3.4	
DMR20:25707301	20	25707301	900	2	1.96E-08	47	5.2	cyp2j20
DMR20:28546801	20	28546801	4700	4	2.52E-10	105	2.2	dpf3
DMR20:30709801	20	30709801	2100	3	1.83E-13	53	2.5	ccr6a
DMR20:32207101	20	32207101	700	3	6.27E-14	35	5	grm1a

DMR20:33274101	20	33274101	1400	2	4.46E-10	11	0.7	ddx1
DMR20:54037401	20	54037401	1800	2	4.51E-09	74	4.1	
DMR20:54276001	20	54276001	1400	3	3.42E-15	53	3.7	
DMR20:54335201	20	54335201	2200	5	4.87E-13	92	4.1	
DMR20:54729401	20	54729401	900	3	1.44E-08	6	0.6	
DMR20:55005801	20	55005801	4000	2	3.16E-09	31	0.7	
DMR21:1920301	21	1920301	1400	3	3.51E-09	38	2.7	WDR7
DMR21:3157401	21	3157401	3800	3	2.16E-08	73	1.9	CTIF
DMR21:7127501	21	7127501	2100	3	2.70E-10	64	3	
DMR21:16032501	21	16032501	11500	3	1.09E-11	93	0.8	
DMR21:19248101	21	19248101	3700	8	3.22E-19	88	2.3	
DMR21:20216801	21	20216801	10800	2	2.03E-09	463	4.2	dkey-247m21.3
DMR21:24438801	21	24438801	1000	3	2.20E-09	42	4.2	
DMR21:24934801	21	24934801	5500	2	2.49E-12	208	3.7	
DMR21:27805601	21	27805601	3500	4	3.13E-12	91	2.6	nrxn2a
DMR21:29267701	21	29267701	3700	2	6.11E-17	83	2.2	BX537120.1
DMR21:29368001	21	29368001	2200	3	1.14E-11	98	4.4	171310;BX537120.1
DMR21:32661901	21	32661901	2500	2	2.75E-20	50	2	
DMR21:34921401	21	34921401	1900	2	1.59E-12	46	2.4	lipia
DMR21:35056001	21	35056001	4200	5	6.95E-10	111	2.6	
DMR21:35114601	21	35114601	1000	2	2.33E-11	30	3	
DMR21:35219601	21	35219601	3900	5	2.49E-11	83	2.1	ubtd2
DMR21:35403401	21	35403401	2500	3	1.15E-11	75	3	dkeyp-23e4.3
DMR21:35457401	21	35457401	6800	2	1.50E-13	157	2.3	dkeyp-23e4.3
DMR21:35759501	21	35759501	1300	2	6.39E-10	38	2.9	sgcd
DMR21:35919401	21	35919401	3800	2	1.68E-11	118	3.1	
DMR21:35931301	21	35931301	2200	2	1.18E-15	46	2	
DMR21:36036601	21	36036601	600	2	1.64E-09	16	2.6	
DMR21:36135601	21	36135601	1300	2	5.93E-10	35	2.6	
DMR21:36469801	21	36469801	700	2	1.69E-09	17	2.4	gabrb4
DMR21:42294601	21	42294601	3800	2	2.41E-10	102	2.6	
DMR21:42333301	21	42333301	6100	3	1.48E-10	131	2.1	
DMR21:44327201	21	44327201	6000	2	2.74E-11	76	1.2	gabra3
DMR22:52901	22	52901	2500	6	9.31E-09	161	6.4	mrpl20
DMR22:4466901	22	4466901	1000	4	2.63E-12	34	3.4	ch73-256j6.7;:ch73-256j6.5
DMR22:4470201	22	4470201	3400	3	9.97E-17	85	2.5	ch73-256j6.7;ch73-256j6.5
DMR22:4561501	22	4561501	700	2	9.99E-09	14	2	
DMR22:9624901	22	9624901	2200	2	3.48E-10	52	2.3	RNH1 (12 of 55)
DMR22:16239001	22	16239001	3600	3	7.93E-11	94	2.6	cdc14ab
DMR22:20722801	22	20722801	1300	2	1.89E-10	31	2.3	amh
DMR22:26599401	22	26599401	6100	4	1.65E-10	105	1.7	capn8;capn2l
DMR22:26625901	22	26625901	2400	2	7.46E-09	47	1.9	capn2l
DMR22:28193801	22	28193801	1500	2	1.34E-10	47	3.1	
DMR22:28243701	22	28243701	8300	3	1.72E-10	162	1.9	
DMR22:29724401	22	29724401	1600	2	4.24E-08	32	2	pdcd4b
DMR22:30129001	22	30129001	4800	3	4.04E-10	126	2.6	add3a
DMR22:30136601	22	30136601	1300	3	3.24E-08	37	2.8	add3a
DMR22:34908801	22	34908801	500	2	1.38E-10	35	7	slit1b
DMR22:37380301	22	37380301	2200	4	7.85E-13	63	2.8	
DMR22:37451201	22	37451201	2200	4	7.33E-13	13	0.5	ch73-334e23.1
DMR22:37456701	22	37456701	1500	2	3.82E-11	87	5.8	ch73-334e23.1
DMR23:2123201	23	2123201	1400	2	7.91E-15	30	2.1	
DMR23:2139901	23	2139901	800	2	1.03E-08	17	2.1	
DMR23:14135001	23	14135001	4700	3	2.23E-11	151	3.2	
DMR23:15622301	23	15622301	2200	2	8.88E-09	34	1.5	

DMR23:44152601	23	44152601	800	2	3.34E-17	28	3.5	CORIN
DMR23:45582101	23	45582101	9500	4	3.94E-10	250	2.6	PSIP1 (1 of 2)
DMR24:331201	24	331201	1400	3	2.59E-25	57	4	
DMR24:834001	24	834001	1300	2	7.78E-26	39	3	napga
DMR24:22719801	24	22719801	2700	7	9.97E-18	63	2.3	
DMR24:24876301	24	24876301	1500	2	1.45E-09	49	3.2	nupl1
DMR24:27134701	24	27134701	1500	6	1.11E-13	53	3.5	dip2ca
DMR24:32406101	24	32406101	1700	2	1.18E-10	40	2.3	
DMR24:33467001	24	33467001	1800	4	6.44E-14	53	2.9	
DMR24:41599801	24	41599801	2700	2	3.76E-08	81	3	
DMR25:1707901	25	1707901	2200	2	1.51E-08	14	0.6	
DMR25:1897301	25	1897301	700	2	6.00E-09	37	5.2	
DMR25:6345401	25	6345401	3800	2	2.20E-08	63	1.6	snx33
DMR25:35284401	25	35284401	2300	3	2.83E-10	79	3.4	cpne8
DMR25:36468801	25	36468801	1300	2	1.05E-09	45	3.4	wwox
DMR25:36680301	25	36680301	900	5	9.21E-12	15	1.6	rfwd3
