# Supplementary material for: Mercury-induced epigenetic transgenerational inheritance of abnormal neurobehavior is correlated with sperm epimutations in zebrafish
Source: PLoS One. 2017 May 2;12(5):e0176155. doi: 10.1371/journal.pone.0176155 (PMC5413066; doi:10.1371/journal.pone.0176155)
Supplement: S5 Table — Information for (A) F0 generation DMR cluster sperm list, and (B) F2 generation DMR cluster sperm list. The DMR cluster is presented with the specific DMR in the cluster listed, chromosome number, start and stop sites, cluster size and minimum p-value of the cluster identification. (PDF) [file pone.0176155.s008.pdf]

Supplemental Table S5

DMR Cluster List and Characteristics

A. F0

chr	start	stop	minP
4	46100000	50200000	0.027821639
4	55750000	60750000	0.013208126
4	65900000	70100000	1.81E-05

B. F2

chr	start	stop	minP
1	52800000	56450000	0.000130448
10	2350000	9200000	0.000614862
11	50000	3150000	0.02621217
17	51150000	53200000	0.02621217
18	1500000	4100000	0.02621217
2	4400000	8.00E+06	0.000614862
21	33950000	37350000	0.000130448
3	1350000	10100000	0.02621217
3	12050000	15950000	4.97E-07
4	70850000	74750000	0.000614862
5	2450000	7600000	0.02621217
5	62700000	72350000	3.71E-06
6	58200000	61250000	0.000130448
8	47150000	49250000	0.02621217
9	18150000	21750000	0.02621217
