# Supplementary material for: Mercury-induced epigenetic transgenerational inheritance of abnormal neurobehavior is correlated with sperm epimutations in zebrafish
Source: PLoS One. 2017 May 2;12(5):e0176155. doi: 10.1371/journal.pone.0176155 (PMC5413066; doi:10.1371/journal.pone.0176155)
Supplement: S6 Table — The DMR name, gene symbol, entrez gene identification, chromosome number, start position site, ensemble gene identifications, gene description, and gene classification category are presented. (PDF) [file pone.0176155.s009.pdf]

Supplemental Table S6 **F0 Sperm DMR Associated Genes**

DMR Name	Gene Symbol	Entrezgene	Chr	start_position	Ensemb #	Gene Description	Functional Category
DMR2:34637701	astn1	791157	2	34589765	ENSDARG00000068323	astrotactin 1	ECM
DMR3:53595001	col5a3a	100330523	3	53518593	ENSDARG00000098294	collagen - type V - alpha 3a	Cytoskeleton
DMR3:57175901	fscn2a	798075	3	57166577	ENSDARG00000059574	fascin actin-bundling protein 2a - retinal	Cytoskeleton
DMR4:6914401	dock4b	654774	4	6825021	ENSDARG00000024874	dedicator of cytokinesis 4b	Cell Cycle
DMR4:7479801	cald1l2	NA	4	7406749	ENSDARG00000070314	caldesmon 1 like 2	Cytoskeleton
DMR4:25320901	sfmbt2	NA	4	25311776	ENSDARG00000045519	Scm-like with four mbt domains 2	Transcription
DMR4:26986301	slc6a1l	NA	4	26983691	ENSDARG00000041205	solute carrier family 6 (neurotransmitter tr	Development
DMR4:29125601	fb11h05	563440	4	28860207	ENSDARG00000074009	fb11h05	NA
DMR4:29131601	fb11h05	563440	4	28860207	ENSDARG00000074009	fb11h05	NA
DMR4:29690601	ch211-214c20.1	NA	4	29666316	ENSDARG00000088082	ch211-214c20.1	NA
DMR4:30279501	RNH1 (6 of 55)	560084	4	30254666	ENSDARG00000089582	ribonuclease/angiogenin inhibitor 1	Translation
DMR4:30506601	dkey-199m13.6	NA	4	30451340	ENSDARG00000102799	dkey-199m13.6	NA
DMR4:33980601	dkey-28i19.3	NA	4	33967055	ENSDARG00000098177	dkey-28i19.3	NA
DMR4:34064901	U1	NA	4	34064976	ENSDARG00000102649	U1 spliceosomal RNA	Translation
DMR4:34064901	U1	NA	4	34066269	ENSDARG00000100840	U1 spliceosomal RNA	Translation
DMR4:36504301	dkeyp-87d1.10	NA	4	36421918	ENSDARG00000103473	dkeyp-87d1.10	NA
DMR4:37866401	RNH1 (39 of 55)	NA	4	37867118	ENSDARG00000102273	ribonuclease/angiogenin inhibitor 1	Translation
DMR4:39532701	dkey-16p6.1	NA	4	39529822	ENSDARG00000104850	dkey-16p6.1	NA
DMR4:39614001	174700	NA	4	39601670	ENSDARG00000091176	174700	NA
DMR4:40931001	ch211-231i17.4	NA	4	40929396	ENSDARG00000092596	ch211-231i17.4	NA
DMR4:43184201	ch211-226o13.1	NA	4	43172804	ENSDARG00000087714	ch211-226o13.1	NA
DMR4:44610401	ch211-162i8.7	NA	4	44507344	ENSDARG00000096216	ch211-162i8.7	NA
DMR4:44695001	dkey-256i11.2	557877	4	44666428	ENSDARG00000093713	dkey-256i11.2	NA
DMR4:44695001	ch211-162i8.7	NA	4	44507344	ENSDARG00000096216	ch211-162i8.7	NA
DMR4:44695001	TRIM14 (4 of 28)	NA	4	44691171	ENSDARG00000087699	tripartite motif containing 14	NA
DMR4:44702301	dkey-256i11.2	557877	4	44666428	ENSDARG00000093713	dkey-256i11.2	NA
DMR4:44702301	ch211-162i8.7	NA	4	44507344	ENSDARG00000096216	ch211-162i8.7	NA
DMR4:44992201	ch211-162i8.7	NA	4	44507344	ENSDARG00000096216	ch211-162i8.7	NA
DMR4:45259701	ch211-215p11.3	NA	4	45245543	ENSDARG00000101450	ch211-215p11.3	NA
DMR4:45955701	dkey-240b24.1	NA	4	45818007	ENSDARG00000098796	dkey-240b24.1	NA
DMR4:45970001	BX470149.1	NA	4	45969200	ENSDARG00000098426		NA
DMR4:45970001	dkey-240b24.1	NA	4	45818007	ENSDARG00000098796	dkey-240b24.1	NA
DMR4:47448201	ch211-42i6.2	NA	4	47446725	ENSDARG00000086130	ch211-42i6.2	NA
DMR4:47454401	ch211-42i6.2	NA	4	47446725	ENSDARG00000086130	ch211-42i6.2	NA
DMR4:47736101	NA	NA	4	47738340	ENSDARG00000088142	NA	NA
DMR4:47792001	ch211-197e7.3	NA	4	47771778	ENSDARG00000088616	ch211-197e7.3	NA
DMR4:47860901	ch211-197e7.2	NA	4	47848850	ENSDARG00000090532	ch211-197e7.2	NA
DMR4:47860901	ch211-197e7.3	NA	4	47771778	ENSDARG00000088616	ch211-197e7.3	NA
DMR4:47860901	dkey-5i16.1	NA	4	47841075	ENSDARG00000088000	dkey-5i16.1	NA
DMR4:47963601	ch211-197e7.1	NA	4	47949687	ENSDARG00000095200	ch211-197e7.1	NA
DMR4:48007701	RNH1 (7 of 55)	NA	4	48010069	ENSDARG00000090160	ribonuclease/angiogenin inhibitor 1	Translation
DMR4:48028101	RNH1 (7 of 55)	NA	4	48010069	ENSDARG00000090160	ribonuclease/angiogenin inhibitor 1	Translation
DMR4:48052301	RNH1 (7 of 55)	NA	4	48010069	ENSDARG00000090160	ribonuclease/angiogenin inhibitor 1	Translation
DMR4:48060201	RNH1 (7 of 55)	NA	4	48010069	ENSDARG00000090160	ribonuclease/angiogenin inhibitor 1	Translation
DMR4:48072401	RNH1 (7 of 55)	NA	4	48010069	ENSDARG00000090160	ribonuclease/angiogenin inhibitor 1	Translation
DMR4:48075001	RNH1 (7 of 55)	NA	4	48010069	ENSDARG00000090160	ribonuclease/angiogenin inhibitor 1	Translation
DMR4:48095701	RNH1 (7 of 55)	NA	4	48010069	ENSDARG00000090160	ribonuclease/angiogenin inhibitor 1	Translation
DMR4:48103601	RNH1 (7 of 55)	NA	4	48010069	ENSDARG00000090160	ribonuclease/angiogenin inhibitor 1	Translation
DMR4:48425601	173619	100006880	4	48337865	ENSDARG00000088961	173619	NA
DMR4:48454401	173619	100006880	4	48337865	ENSDARG00000088961	173619	NA
DMR4:48454401	dkey-16p6.1	NA	4	48445504	ENSDARG00000089179	dkey-16p6.1	NA
DMR4:48593601	173619	100006880	4	48337865	ENSDARG00000088961	173619	NA
DMR4:49333101	dkey-82i20.3	100005493	4	49306725	ENSDARG00000102735	dkey-82i20.3	NA
DMR4:49333101		NA	4	49336148	ENSDARG00000098306		NA
DMR4:49361201	dkey-82i20.3	100005493	4	49306725	ENSDARG00000102735	dkey-82i20.3	NA
DMR4:49361201		NA	4	49336148	ENSDARG00000098306		NA
DMR4:49379001	dkey-82i20.3	100005493	4	49306725	ENSDARG00000102735	dkey-82i20.3	NA
DMR4:49379001		NA	4	49336148	ENSDARG00000098306		NA
DMR4:49421301	dkey-82i20.3	100005493	4	49306725	ENSDARG00000102735	dkey-82i20.3	NA
DMR4:49436801	NA	NA	4	49429387	ENSDARG00000101729	NA	NA
DMR4:50122001	dkey-22a18.1	NA	4	50100997	ENSDARG00000099626	dkey-22a18.1	NA
DMR4:51339401	dkey-250k10.1	NA	4	51338256	ENSDARG00000102595	dkey-250k10.1	NA
DMR4:51347901	dkey-250k10.1	NA	4	51338256	ENSDARG00000102595	dkey-250k10.1	NA
DMR4:51353101	dkey-250k10.1	NA	4	51338256	ENSDARG00000102595	dkey-250k10.1	NA
DMR4:52939601	dkey-56m15.9	NA	4	52931823	ENSDARG00000103281	dkey-56m15.9	NA
DMR4:52956401	dkey-56m15.9	NA	4	52931823	ENSDARG00000103281	dkey-56m15.9	NA

DMR4:53449201	dkey-257e4.2	NA	4	53436793	ENSDARG00000101997	dkey-257e4.2	NA
DMR4:53590201	ZFP28 (17 of 19)	NA	4	53577718	ENSDARG00000103031	ZFP28 zinc finger protein	NA
DMR4:54485701	ch211-237a4.2	NA	4	54459778	ENSDARG00000098585	ch211-237a4.2	NA
DMR4:57066201	ch211-233h3.1	NA	4	57035021	ENSDARG00000102439	ch211-233h3.1	NA
DMR4:57094301	ch211-233h3.1	NA	4	57035021	ENSDARG00000102439	ch211-233h3.1	NA
DMR4:57106001	ch211-233h3.1	NA	4	57035021	ENSDARG00000102439	ch211-233h3.1	NA
DMR4:57147501	RNH1 (4 of 55)	NA	4	57146255	ENSDARG00000077697	ribonuclease/angiogenin inhibitor 1	Translation
DMR4:57147501	ch211-241n15.1	NA	4	57135167	ENSDARG00000076255	ch211-241n15.1	NA
DMR4:57230701	ch211-241n15.1	NA	4	57135167	ENSDARG00000076255	ch211-241n15.1	NA
DMR4:57308501	ch211-241n15.1	NA	4	57135167	ENSDARG00000076255	ch211-241n15.1	NA
DMR4:57453101	dkey-149m13.2	NA	4	57437413	ENSDARG00000094572	dkey-149m13.2	NA
DMR4:57635201	173710	569852	4	57611805	ENSDARG00000078728	173710	NA
DMR4:57782601	dkey-196n19.2	NA	4	57741506	ENSDARG00000090734	dkey-196n19.2	NA
DMR4:57928701	ch211-178j18.2	NA	4	57902138	ENSDARG00000092475	ch211-178j18.2	NA
DMR4:58020001	ch211-178j18.4	NA	4	58021494	ENSDARG00000096024	ch211-178j18.4	NA
DMR4:58047101	U1	NA	4	58051432	ENSDARG00000091853	U1 spliceosomal RNA	Translation
DMR4:58063601	U1	NA	4	58077458	ENSDARG00000090928	U1 spliceosomal RNA	Translation
DMR4:58063601	U1	NA	4	58076153	ENSDARG00000088668	U1 spliceosomal RNA	Translation
DMR4:58063601	U1	NA	4	58070876	ENSDARG00000091132	U1 spliceosomal RNA	Translation
DMR4:58063601	U1	NA	4	58080073	ENSDARG00000089029	U1 spliceosomal RNA	Translation
DMR4:58063601	U1	NA	4	58066220	ENSDARG00000087997	U1 spliceosomal RNA	Translation
DMR4:58063601	U1	NA	4	58074749	ENSDARG00000090006	U1 spliceosomal RNA	Translation
DMR4:58063601	U1	NA	4	58073457	ENSDARG00000091354	U1 spliceosomal RNA	Translation
DMR4:58063601	U1	NA	4	58078769	ENSDARG00000089332	U1 spliceosomal RNA	Translation
DMR4:58063601	U1	NA	4	58072181	ENSDARG00000088282	U1 spliceosomal RNA	Translation
DMR4:58063601	U1	NA	4	58064915	ENSDARG00000091196	U1 spliceosomal RNA	Translation
DMR4:58063601	U1	NA	4	58081377	ENSDARG00000088327	U1 spliceosomal RNA	Translation
DMR4:58063601	U1	NA	4	58063605	ENSDARG00000086219	U1 spliceosomal RNA	Translation
DMR4:58063601	U1	NA	4	58069566	ENSDARG00000088683	U1 spliceosomal RNA	Translation
DMR4:58063601	U1	NA	4	58067718	ENSDARG00000086900	U1 spliceosomal RNA	Translation
DMR4:58271901	BX465848.1	NA	4	58235813	ENSDARG00000104146		NA
DMR4:58271901	dkey-248e17.9	100537855	4	58189793	ENSDARG00000101524	dkey-248e17.9	NA
DMR4:58323301	dkey-248e17.7	NA	4	58323857	ENSDARG00000099036	dkey-248e17.7	NA
DMR4:58323301	dkey-248e17.9	100537855	4	58189793	ENSDARG00000101524	dkey-248e17.9	NA
DMR4:58637101	dkey-211i20.5	NA	4	58629786	ENSDARG00000105072	dkey-211i20.5	NA
DMR4:58637101	dkey-211i20.2	NA	4	58565040	ENSDARG00000103003	dkey-211i20.2	NA
DMR4:58654201	dkey-211i20.2	NA	4	58565040	ENSDARG00000103003	dkey-211i20.2	NA
DMR4:58668001	dkey-211i20.2	NA	4	58565040	ENSDARG00000103003	dkey-211i20.2	NA
DMR4:58705601	dkey-211i20.2	NA	4	58565040	ENSDARG00000103003	dkey-211i20.2	NA
DMR4:58720601	dkey-211i20.2	NA	4	58565040	ENSDARG00000103003	dkey-211i20.2	NA
DMR4:58888201	dkey-204l2.3	NA	4	58842629	ENSDARG00000093803	dkey-204l2.3	NA
DMR4:58954601	dkey-204l2.3	NA	4	58842629	ENSDARG00000093803	dkey-204l2.3	NA
DMR4:58967201	dkey-204l2.3	NA	4	58842629	ENSDARG00000093803	dkey-204l2.3	NA
DMR4:58969801	dkey-204l2.3	NA	4	58842629	ENSDARG00000093803	dkey-204l2.3	NA
DMR4:59125101	dkey-9p20.9	NA	4	59126826	ENSDARG00000092102	dkey-9p20.9	NA
DMR4:59125101	dkey-9p20.8	NA	4	59129413	ENSDARG00000095433	dkey-9p20.8	NA
DMR4:59125101	dkey-9p20.7	NA	4	59131210	ENSDARG00000095097	dkey-9p20.7	NA
DMR4:59125101	dkey-9p20.6	NA	4	59133007	ENSDARG00000092010	dkey-9p20.6	NA
DMR4:59125101	dkey-9p20.5	NA	4	59134804	ENSDARG00000093261	dkey-9p20.5	NA
DMR4:59125101	dkey-9p20.4	NA	4	59136601	ENSDARG00000092156	dkey-9p20.4	NA
DMR4:59554401	U1	NA	4	59559693	ENSDARG00000104060	U1 spliceosomal RNA	Translation
DMR4:59554401	U1	NA	4	59557083	ENSDARG00000103794	U1 spliceosomal RNA	Translation
DMR4:59554401	U1	NA	4	59588403	ENSDARG00000101376	U1 spliceosomal RNA	Translation
DMR4:59554401	U1	NA	4	59568828	ENSDARG00000099335	U1 spliceosomal RNA	Translation
DMR4:59554401	U1	NA	4	59566218	ENSDARG00000099671	U1 spliceosomal RNA	Translation
DMR4:59554401	U1	NA	4	59560998	ENSDARG00000101416	U1 spliceosomal RNA	Translation
DMR4:59554401	U1	NA	4	59567523	ENSDARG00000099992	U1 spliceosomal RNA	Translation
DMR4:59554401	U1	NA	4	59570133	ENSDARG00000101546	U1 spliceosomal RNA	Translation
DMR4:59554401	U1	NA	4	59562303	ENSDARG00000104796	U1 spliceosomal RNA	Translation
DMR4:59554401	U1	NA	4	59585793	ENSDARG00000100489	U1 spliceosomal RNA	Translation
DMR4:59554401	U1	NA	4	59554481	ENSDARG00000098307	U1 spliceosomal RNA	Translation
DMR4:59554401	U1	NA	4	59555778	ENSDARG00000100884	U1 spliceosomal RNA	Translation
DMR4:59554401	U1	NA	4	59580573	ENSDARG00000104151	U1 spliceosomal RNA	Translation
DMR4:59554401	U1	NA	4	59571438	ENSDARG00000102686	U1 spliceosomal RNA	Translation
DMR4:59554401	U1	NA	4	59584488	ENSDARG00000104001	U1 spliceosomal RNA	Translation
DMR4:59554401	U1	NA	4	59579268	ENSDARG00000103385	U1 spliceosomal RNA	Translation
DMR4:59554401	U1	NA	4	59574048	ENSDARG00000103387	U1 spliceosomal RNA	Translation
DMR4:59554401	U1	NA	4	59583183	ENSDARG00000101228	U1 spliceosomal RNA	Translation
DMR4:59554401	U1	NA	4	59576658	ENSDARG00000101153	U1 spliceosomal RNA	Translation

DMR4:59554401	U1	NA	4	59575353	ENSDARG00000103325	U1 spliceosomal RNA	Translation
DMR4:59554401	U1	NA	4	59581878	ENSDARG00000104168	U1 spliceosomal RNA	Translation
DMR4:59554401	U1	NA	4	59572743	ENSDARG00000105216	U1 spliceosomal RNA	Translation
DMR4:59554401	U1	NA	4	59564913	ENSDARG00000103812	U1 spliceosomal RNA	Translation
DMR4:59554401	U1	NA	4	59563608	ENSDARG00000102231	U1 spliceosomal RNA	Translation
DMR4:59554401	U1	NA	4	59589708	ENSDARG00000098648	U1 spliceosomal RNA	Translation
DMR4:59554401	U1	NA	4	59577963	ENSDARG00000101993	U1 spliceosomal RNA	Translation
DMR4:59554401	U1	NA	4	59587098	ENSDARG00000101487	U1 spliceosomal RNA	Translation
DMR4:59554401	U1	NA	4	59558388	ENSDARG00000103990	U1 spliceosomal RNA	Translation
DMR4:59779301	dkey-199m13.1	NA	4	59770725	ENSDARG00000100335	dkey-199m13.1	NA
DMR4:59905301	dkey-146c18.5	NA	4	59889283	ENSDARG00000098094	dkey-146c18.5	NA
DMR4:59925901	RNH1 (37 of 55)	NA	4	59917259	ENSDARG00000102212	ribonuclease/angiogenin inhibitor 1	Translation
DMR4:59925901	dkey-146c18.5	NA	4	59889283	ENSDARG00000098094	dkey-146c18.5	NA
DMR4:59955601	dkey-146c18.5	NA	4	59889283	ENSDARG00000098094	dkey-146c18.5	NA
DMR4:63181501	dkey-9i5.5	NA	4	63148826	ENSDARG00000105217	dkey-9i5.5	NA
DMR4:63477901	dkey-14o6.1	NA	4	63458198	ENSDARG00000098383	dkey-14o6.1	NA
DMR4:63477901	ZFP28 (8 of 19)	NA	4	63430193	ENSDARG00000098126	ZFP28 zinc finger protein	NA
DMR4:64608901	174311	100126141	4	64539369	ENSDARG00000099048	174311	NA
DMR4:64608901	174311	100137121	4	64539369	ENSDARG00000099048	174311	NA
DMR4:64644301	174311	100126141	4	64539369	ENSDARG00000099048	174311	NA
DMR4:64644301	174311	100137121	4	64539369	ENSDARG00000099048	174311	NA
DMR4:66478801	dkey-237g15.2	NA	4	66451358	ENSDARG00000100302	dkey-237g15.2	NA
DMR4:66478801	dkey-16p6.1	NA	4	66450508	ENSDARG00000099262	dkey-16p6.1	NA
DMR4:66805101	dkey-264f17.1	NA	4	66796875	ENSDARG00000100219	dkey-264f17.1	NA
DMR4:66805101	dkey-264f17.5	NA	4	66796189	ENSDARG00000104502	dkey-264f17.5	NA
DMR4:67433401	U1	NA	4	67453741	ENSDARG00000105257	U1 spliceosomal RNA	Translation
DMR4:67433401	U1	NA	4	67437127	ENSDARG00000102263	U1 spliceosomal RNA	Translation
DMR4:67433401	U1	NA	4	67435849	ENSDARG00000101603	U1 spliceosomal RNA	Translation
DMR4:67433401	U1	NA	4	67457575	ENSDARG00000099856	U1 spliceosomal RNA	Translation
DMR4:67433401	U1	NA	4	67451185	ENSDARG00000104723	U1 spliceosomal RNA	Translation
DMR4:67433401	U1	NA	4	67440961	ENSDARG00000103758	U1 spliceosomal RNA	Translation
DMR4:67433401	U1	NA	4	67456297	ENSDARG00000101440	U1 spliceosomal RNA	Translation
DMR4:67433401	U1	NA	4	67439683	ENSDARG00000105211	U1 spliceosomal RNA	Translation
DMR4:67433401	U1	NA	4	67446073	ENSDARG00000104772	U1 spliceosomal RNA	Translation
DMR4:67433401	U1	NA	4	67452463	ENSDARG00000104411	U1 spliceosomal RNA	Translation
DMR4:67433401	U1	NA	4	67444795	ENSDARG00000100852	U1 spliceosomal RNA	Translation
DMR4:67433401	U1	NA	4	67438405	ENSDARG00000102481	U1 spliceosomal RNA	Translation
DMR4:67433401	U1	NA	4	67458853	ENSDARG00000101834	U1 spliceosomal RNA	Translation
DMR4:67433401	U1	NA	4	67447351	ENSDARG00000103557	U1 spliceosomal RNA	Translation
DMR4:67433401	U1	NA	4	67443517	ENSDARG00000102979	U1 spliceosomal RNA	Translation
DMR4:67433401	U1	NA	4	67455019	ENSDARG00000104503	U1 spliceosomal RNA	Translation
DMR4:67433401	U1	NA	4	67442239	ENSDARG00000101570	U1 spliceosomal RNA	Translation
DMR4:67433401	U1	NA	4	67448629	ENSDARG00000102169	U1 spliceosomal RNA	Translation
DMR4:67433401	U1	NA	4	67449907	ENSDARG00000100841	U1 spliceosomal RNA	Translation
DMR4:67491001	ch211-120c15.2	NA	4	67483655	ENSDARG00000102819	ch211-120c15.2	NA
DMR4:67494501	ch211-120c15.2	NA	4	67483655	ENSDARG00000102819	ch211-120c15.2	NA
DMR4:67506201	ch211-120c15.2	NA	4	67483655	ENSDARG00000102819	ch211-120c15.2	NA
DMR4:67527001	dkey-246j6.2	NA	4	67518275	ENSDARG00000098384	dkey-246j6.2	NA
DMR4:67527001	ch211-120c15.2	NA	4	67483655	ENSDARG00000102819	ch211-120c15.2	NA
DMR4:67553101	dkey-246j6.2	NA	4	67518275	ENSDARG00000098384	dkey-246j6.2	NA
DMR4:67553101	ch211-120c15.2	NA	4	67483655	ENSDARG00000102819	ch211-120c15.2	NA
DMR4:67560501	dkey-246j6.2	NA	4	67518275	ENSDARG00000098384	dkey-246j6.2	NA
DMR4:67560501	ch211-120c15.2	NA	4	67483655	ENSDARG00000102819	ch211-120c15.2	NA
DMR4:67567601	ch211-120c15.3	101885268	4	67567117	ENSDARG00000101807	ch211-120c15.3	NA
DMR4:67567601	dkey-246j6.2	NA	4	67518275	ENSDARG00000098384	dkey-246j6.2	NA
DMR4:67567601	ch211-120c15.2	NA	4	67483655	ENSDARG00000102819	ch211-120c15.2	NA
DMR4:67615701	ch211-120c15.2	NA	4	67483655	ENSDARG00000102819	ch211-120c15.2	NA
DMR4:67696201	174944	NA	4	67686010	ENSDARG00000105091	174944	NA
DMR4:67751901	dkey-238o14.6	NA	4	67756436	ENSDARG00000104561	dkey-238o14.6	NA
DMR4:67828601	dkey-238o14.9	565962	4	67836797	ENSDARG00000103307	dkey-238o14.9	NA
DMR4:67928701	U1	NA	4	67942851	ENSDARG00000101801	U1 spliceosomal RNA	Translation
DMR4:67928701	U1	NA	4	67961121	ENSDARG00000104510	U1 spliceosomal RNA	Translation
DMR4:67928701	U1	NA	4	67974171	ENSDARG00000104971	U1 spliceosomal RNA	Translation
DMR4:67928701	U1	NA	4	67957206	ENSDARG00000104021	U1 spliceosomal RNA	Translation
DMR4:67928701	U1	NA	4	67968951	ENSDARG00000101241	U1 spliceosomal RNA	Translation
DMR4:67928701	U1	NA	4	67941546	ENSDARG00000099261	U1 spliceosomal RNA	Translation
DMR4:67928701	U1	NA	4	67971561	ENSDARG00000098156	U1 spliceosomal RNA	Translation
DMR4:67928701	U1	NA	4	67948071	ENSDARG00000100682	U1 spliceosomal RNA	Translation
DMR4:67928701	U1	NA	4	67931133	ENSDARG00000104464	U1 spliceosomal RNA	Translation

DMR4:67928701	U1	NA	4	67949376	ENSDARG00000102136	U1 spliceosomal RNA	Translation
DMR4:67928701	U1	NA	4	67940241	ENSDARG00000103168	U1 spliceosomal RNA	Translation
DMR4:67928701	U1	NA	4	67967646	ENSDARG00000103475	U1 spliceosomal RNA	Translation
DMR4:67928701	U1	NA	4	67932411	ENSDARG00000104909	U1 spliceosomal RNA	Translation
DMR4:67928701	U1	NA	4	67950681	ENSDARG00000103896	U1 spliceosomal RNA	Translation
DMR4:67928701	U1	NA	4	67963731	ENSDARG00000099012	U1 spliceosomal RNA	Translation
DMR4:67928701	U1	NA	4	67946766	ENSDARG00000103389	U1 spliceosomal RNA	Translation
DMR4:67928701	U1	NA	4	67976781	ENSDARG00000101854	U1 spliceosomal RNA	Translation
DMR4:67928701	U1	NA	4	67958511	ENSDARG00000102190	U1 spliceosomal RNA	Translation
DMR4:67928701	U1	NA	4	67966341	ENSDARG00000101031	U1 spliceosomal RNA	Translation
DMR4:67928701	U1	NA	4	67935021	ENSDARG00000104055	U1 spliceosomal RNA	Translation
DMR4:67928701	U1	NA	4	67953291	ENSDARG00000100579	U1 spliceosomal RNA	Translation
DMR4:67928701	U1	NA	4	67929828	ENSDARG00000098548	U1 spliceosomal RNA	Translation
DMR4:67928701	U1	NA	4	67954596	ENSDARG00000099041	U1 spliceosomal RNA	Translation
DMR4:67928701	U1	NA	4	67944156	ENSDARG00000100615	U1 spliceosomal RNA	Translation
DMR4:67928701	U1	NA	4	67955901	ENSDARG00000102485	U1 spliceosomal RNA	Translation
DMR4:67928701	U1	NA	4	67937631	ENSDARG00000098948	U1 spliceosomal RNA	Translation
DMR4:67928701	U1	NA	4	67962426	ENSDARG00000100937	U1 spliceosomal RNA	Translation
DMR4:67928701	U1	NA	4	67975476	ENSDARG00000100369	U1 spliceosomal RNA	Translation
DMR4:67928701	U1	NA	4	67945461	ENSDARG00000099296	U1 spliceosomal RNA	Translation
DMR4:67928701	U1	NA	4	67951986	ENSDARG00000100125	U1 spliceosomal RNA	Translation
DMR4:67928701	U1	NA	4	67933716	ENSDARG00000090218	U1 spliceosomal RNA	Translation
DMR4:67928701	U1	NA	4	67970256	ENSDARG00000104988	U1 spliceosomal RNA	Translation
DMR4:67928701	U1	NA	4	67972866	ENSDARG00000099734	U1 spliceosomal RNA	Translation
DMR4:67928701	U1	NA	4	67936326	ENSDARG00000100984	U1 spliceosomal RNA	Translation
DMR4:67928701	U1	NA	4	67959816	ENSDARG00000098814	U1 spliceosomal RNA	Translation
DMR4:67928701	U1	NA	4	67965036	ENSDARG00000103161	U1 spliceosomal RNA	Translation
DMR4:67928701	U1	NA	4	67978060	ENSDARG00000099341	U1 spliceosomal RNA	Translation
DMR4:67928701	U1	NA	4	67938936	ENSDARG00000103126	U1 spliceosomal RNA	Translation
DMR4:68042201	dkey-3h2.4	NA	4	67990652	ENSDARG00000098444	dkey-3h2.4	NA
DMR4:68086601	dkey-3h2.4	NA	4	67990652	ENSDARG00000098444	dkey-3h2.4	NA
DMR4:68116701	dkey-3h2.4	NA	4	67990652	ENSDARG00000098444	dkey-3h2.4	NA
DMR4:68128701	dkey-3h2.4	NA	4	67990652	ENSDARG00000098444	dkey-3h2.4	NA
DMR4:68175701	dkey-3h2.4	NA	4	67990652	ENSDARG00000098444	dkey-3h2.4	NA
DMR4:68185601	dkey-3h2.4	NA	4	67990652	ENSDARG00000098444	dkey-3h2.4	NA
DMR4:68210601	dkey-3h2.4	NA	4	67990652	ENSDARG00000098444	dkey-3h2.4	NA
DMR4:68260701	dkey-3h2.4	NA	4	67990652	ENSDARG00000098444	dkey-3h2.4	NA
DMR4:68306001	dkey-29j8.1	NA	4	68290913	ENSDARG00000101272	dkey-29j8.1	NA
DMR4:68326801	TRIM14 (24 of 28)	NA	4	68315077	ENSDARG00000103371	tripartite motif containing 14	NA
DMR4:68702301	dkey-28k24.2	NA	4	68686839	ENSDARG00000104787	dkey-28k24.2	NA
DMR4:68804101	dkey-254e13.6	NA	4	68765919	ENSDARG00000103324	dkey-254e13.6	NA
DMR4:69312801	ch211-76m11.3	NA	4	69292827	ENSDARG00000104890	ch211-76m11.3	NA
DMR4:69312801	ch211-76m11.5	NA	4	69278330	ENSDARG00000100006	ch211-76m11.5	NA
DMR4:69312801	ch211-76m11.11	NA	4	69106827	ENSDARG00000101828	ch211-76m11.11	NA
DMR4:69367501	ch211-76m11.8	NA	4	69340358	ENSDARG00000098305	ch211-76m11.8	NA
DMR4:69367501	ch211-76m11.3	NA	4	69292827	ENSDARG00000104890	ch211-76m11.3	NA
DMR4:69367501	ch211-76m11.5	NA	4	69278330	ENSDARG00000100006	ch211-76m11.5	NA
DMR4:69367501	ch211-76m11.11	NA	4	69106827	ENSDARG00000101828	ch211-76m11.11	NA
DMR4:69450801	ch211-76m11.5	NA	4	69278330	ENSDARG00000100006	ch211-76m11.5	NA
DMR4:69450801	ch211-76m11.11	NA	4	69106827	ENSDARG00000101828	ch211-76m11.11	NA
DMR4:69458101	ch211-76m11.5	NA	4	69278330	ENSDARG00000100006	ch211-76m11.5	NA
DMR4:69458101	ch211-76m11.11	NA	4	69106827	ENSDARG00000101828	ch211-76m11.11	NA
DMR4:69461901	ch211-76m11.5	NA	4	69278330	ENSDARG00000100006	ch211-76m11.5	NA
DMR4:69461901	ch211-76m11.11	NA	4	69106827	ENSDARG00000101828	ch211-76m11.11	NA
DMR4:69498101	ch211-76m11.5	NA	4	69278330	ENSDARG00000100006	ch211-76m11.5	NA
DMR4:69498101	ch211-76m11.11	NA	4	69106827	ENSDARG00000101828	ch211-76m11.11	NA
DMR4:69560401	dkey-27n6.4	NA	4	69552224	ENSDARG00000101757	dkey-27n6.4	NA
DMR4:69675101	dkeyp-4f2.1	NA	4	69668871	ENSDARG00000103781	dkeyp-4f2.1	NA
DMR4:71674701	162958	334070	4	71652635	ENSDARG00000091869	162958	NA
DMR4:71674701	162958	799470	4	71652635	ENSDARG00000091869	162958	NA
DMR4:73395701	PHF21B	NA	4	73251704	ENSDARG00000087887	PHD finger protein 21B	Protease
DMR4:74622301	ch211-106j21.5	NA	4	74611258	ENSDARG00000104966	ch211-106j21.5	NA
DMR4:74622301	dkey-261j11.2	NA	4	74084251	ENSDARG00000101551	dkey-261j11.2	NA
DMR4:74622301	174944	103911006	4	74050132	ENSDARG00000099327	174944	NA
DMR4:76592301	pacsin2	404601	4	76548382	ENSDARG00000078014	protein kinase C and casein kinase substrate in neurons 2	Signaling
DMR4:76592301	terfa	192316	4	76587747	ENSDARG00000039302	telomeric repeat binding factor a	Development
DMR4:76592301	CKAP4	406549	4	76590519	ENSDARG00000032405	cytoskeleton-associated protein 4	Cytoskeleton

DMR5:433201	thap1	692315	5	424738	ENSDARG00000059020	THAP domain containing - apoptosis associated protein 1	Apoptosis
DMR7:19951901	CU019646.1	794738	7	19895191	ENSDARG00000036414	Uncharacterized protein [Source:UniProtKB/TrEMBL;Acc:E7F0F4]	NA
DMR8:52201101	tcf7l1b	30556	8	52121215	ENSDARG00000007369	transcription factor 7-like 1b (T-cell specific - HMG-box)	Transcription
DMR8:52435501	gins4	572562	8	52429014	ENSDARG00000016044	GINS complex subunit 4 (Sld5 homolog)	NA
DMR8:53196401	cacna1db	NA	8	53026462	ENSDARG00000101589	calcium channel - voltage-dependent - L type - alpha 1D subunit - b	Signaling
DMR9:53496501	CABZ01054962.1	799204	9	53471604	ENSDARG00000088672	Uncharacterized protein	NA
DMR10:6863601	ppip5k2	449862	10	6822414	ENSDARG00000078441	diphosphoinositol pentakisphosphate kinase 2	Signaling
DMR11:27863601	ch211-220m17.6	566750	11	27862229	ENSDARG00000022570	ch211-220m17.6	NA
DMR11:29151701	arhgef10la	799435	11	29083968	ENSDARG00000088630	Rho guanine nucleotide exchange factor (GEF) 10-like a	Signaling
DMR14:51898101	atp6v0e1	450017	14	51855440	ENSDARG00000101794	ATPase - H+ transporting - lysosomal V0 subunit e1	Signaling
DMR15:30691901	msi2b	394084	15	30543904	ENSDARG00000032614	musashi RNA-binding protein 2b	Translation
DMR15:39669001	robo1	30769	15	39431053	ENSDARG00000026784	roundabout - axon guidance receptor - homolog 1 (Drosophila)	Receptor
DMR15:39828301	t2gtl4a	NA	15	39782778	ENSDARG00000094788	tol2 transposon-derived gene trap locus 4A	NA
DMR15:39906501	CR626875.1	NA	15	39907939	ENSDARG00000103175		NA
DMR15:39906501	t2gtl4a	NA	15	39782778	ENSDARG00000094788	tol2 transposon-derived gene trap locus 4A	NA
DMR15:47004301	inppl1a	NA	15	46988351	ENSDARG00000104222	inositol polyphosphate phosphatase-like 1a	Signaling
DMR16:36379601	CR974440.1	NA	16	36395410	ENSDARG00000102416	NA	NA
DMR17:50945101	aqr	393436	17	50930341	ENSDARG00000016775	aquarius intron-binding spliceosomal factor	Translation
DMR17:51062001	paplna	562930	17	51034383	ENSDARG00000027867	papilin a - proteoglycan-like sulfated glycoprotein	ECM
DMR17:51144601	trappc12	791162	17	51142175	ENSDARG00000060101	trafficking protein particle complex 12	NA
DMR20:5911101	nrxn3b	570698	20	5518645	ENSDARG00000062693	neurexin 3b	Receptor
DMR21:18842201	dkey-65l23.2	793688	21	18840494	ENSDARG00000093234	dkey-65l23.2	NA
DMR21:18842201	dkey-65l23.1	NA	21	18830340	ENSDARG00000094538	dkey-65l23.1	NA
DMR22:53601	mrpl20	751640	22	19300	ENSDARG00000090462	mitochondrial ribosomal protein L20	Translation
DMR22:676601	arl8a	567222	22	673036	ENSDARG00000078686	ADP-ribosylation factor-like 8A	Signaling
DMR22:3303901	gipc3	724013	22	3284403	ENSDARG00000053074	GIPC PDZ domain containing family - member 3	Metabolism
DMR22:34701701	CU896691.1	NA	22	34657153	ENSDARG00000087901	Uncharacterized protein	NA
DMR22:35403901	HTR3C	NA	22	35401647	ENSDARG00000077959	5-hydroxytryptamine (serotonin) receptor 3C - ionotropic	Receptor
DMR23:8733301	rgs19	100170801	23	8671601	ENSDARG00000077385	regulator of G-protein signaling 19	Signaling
DMR23:39923401	ppp1r14c	568055	23	39914946	ENSDARG00000077341	protein phosphatase 1 - regulatory (inhibitor) subunit 14C	Signaling
DMR23:44716801	eif4e2rs1	393732	23	44622538	ENSDARG00000015835	eukaryotic translation initiation factor 4E family member 2 related sequence 1	Translation
DMR23:44716801	DLG4 (1 of 2)	100148055	23	44502592	ENSDARG00000039385	discs - large homolog 4 (Drosophila)	Development
DMR24:2446201	rreb1a	338237	24	2350529	ENSDARG00000063701	ras responsive element binding protein 1a	Signaling
