# Supplementary material for: Mercury-induced epigenetic transgenerational inheritance of abnormal neurobehavior is correlated with sperm epimutations in zebrafish
Source: PLoS One. 2017 May 2;12(5):e0176155. doi: 10.1371/journal.pone.0176155 (PMC5413066; doi:10.1371/journal.pone.0176155)
Supplement: S7 Table — The DMR name, gene symbol, entrez gene identification, chromosome number, start position site, ensemble gene identifications, gene description, and gene classification category are presented. (PDF) [file pone.0176155.s010.pdf]

Supplemental Table S7 **F2 Sperm DMR Associated Genes**

DMR Name	Gene Symbol	Entrezgene	Chr	start_position	Ensembl #	Gene Description	Functional Category
DMR1:138101	f10	282670	1	135903	ENSDARG00000088581	coagulation factor X	Protein Binding
DMR1:138101	PROZ (2 of 2)	558106	1	144284	ENSDARG00000076900	protein Z - vitamin K-dependent plasma glycoprotein	NA
DMR1:138101	f7i	282671	1	127250	ENSDARG00000075827	coagulation factor VIIi	Protein Binding
DMR1:1820401	PTGFRN (1 of 2)	101884699	1	1793237	ENSDARG00000075505	prostaglandin F2 receptor inhibitor	NA
DMR1:30600701	rims1b	568486	1	30570770	ENSDARG00000078902	regulating synaptic membrane exocytosis 1b	NA
DMR1:30614301	rims1b	568486	1	30570770	ENSDARG00000078902	regulating synaptic membrane exocytosis 1b	NA
DMR1:30638601	rims1b	568486	1	30570770	ENSDARG00000078902	regulating synaptic membrane exocytosis 1b [	NA
DMR1:44449201	mucms1	NA	1	44448541	ENSDARG00000069566	mucin - multiple PTS and SEA group - member 1	NA
DMR1:47308001	cfap58	561732	1	47140387	ENSDARG00000069435	cilia and flagella associated protein 58	NA
DMR1:48885801	ch211-281g13.5	100148091	1	48880007	ENSDARG00000074052	ch211-281g13.5	NA
DMR1:54091401	136791	678565	1	54089292	ENSDARG00000091320	136791	NA
DMR1:54259701	crtac1a	559626	1	54256871	ENSDARG00000059826	cartilage acidic protein 1a	NA
DMR1:54466201	mb	393558	1	54460292	ENSDARG00000031952	myoglobin	Binding Protein
DMR1:54529001	mri1	100002302	1	54513499	ENSDARG00000075754	methylthioribose-1-phosphate isomerase 1	Translation
DMR1:54750201	SIGLEC1 (24 of 51)	NA	1	54760763	ENSDARG00000090329	sialic acid binding Ig-like lectin 1 - sialoadhesin	NA
DMR1:54750201	SIGLEC1 (51 of 51)	NA	1	54752427	ENSDARG00000103489	sialic acid binding Ig-like lectin 1 - sialoadhesin	NA
DMR1:54788101	SIGLEC1 (51 of 51)	NA	1	54752427	ENSDARG00000103489	sialic acid binding Ig-like lectin 1 - sialoadhesin	NA
DMR1:54966901	dkey-9c18.12	100536772	1	54965547	ENSDARG00000041399	dkey-9c18.12	NA
DMR1:55989101	CABZ01059415.2	100334082	1	55990642	ENSDARG00000087345	Uncharacterized protein	NA
DMR1:57335801	ch211-114l13.4	796740	1	57330427	ENSDARG00000078246	ch211-114l13.4	NA
DMR1:57340601	ch211-114l13.4	796740	1	57330427	ENSDARG00000078246	ch211-114l13.4	NA
DMR1:57373101	caspbl	566185	1	57370077	ENSDARG00000094433	caspase b - like	Apoptosis
DMR2:1532301	adgrl2b.1	101882597	2	1371893	ENSDARG00000075899	adhesion G protein-coupled receptor L2b - tandem duplicate 1	ECM
DMR2:2001801	pth1ra	30629	2	1947342	ENSDARG00000020957	parathyroid hormone 1 receptor a	Receptor
DMR2:4909301	tnk2b	559119	2	4888417	ENSDARG00000104409	tyrosine kinase - non-receptor - 2b	Signaling
DMR2:4915001	tnk2b	559119	2	4888417	ENSDARG00000104409	tyrosine kinase - non-receptor - 2b	Signaling
DMR2:5487101	DUSP18	100005114	2	5485182	ENSDARG00000078598	dual specificity phosphatase 18	Signaling
DMR2:6025601	tmem125b	797385	2	6022020	ENSDARG00000075980	transmembrane protein 125b	NA
DMR2:6174001	aldh9a1b	399481	2	6155489	ENSDARG00000037061	aldehyde dehydrogenase 9 family - member A1b	Metabolism
DMR2:6392701	otol1a	553759	2	6391757	ENSDARG00000001771	otolin 1a	NA
DMR2:6417001	SMC6	557127	2	6409741	ENSDARG00000058719	structural maintenance of chromosomes 6	NA
DMR2:8129401	ephb3a	30313	2	8125959	ENSDARG00000031548	eph receptor B3a	Receptor
DMR2:10654201	scinlb	406363	2	10612980	ENSDARG00000058348	scinderin like b	NA
DMR2:11311101	lrrc53	101882576	2	11299712	ENSDARG00000097415	leucine rich repeat containing 53	NA
DMR2:34632601	astn1	791157	2	34589765	ENSDARG00000068323	astrotactin 1	NA
DMR2:44005601	nlrb5	NA	2	43998985	ENSDARG00000090699	NOD-like receptor family B - member 5	NA
DMR2:56637701	CABZ01024426.1	NA	2	56641206	ENSDARG00000011283	Uncharacterized protein	NA
DMR2:58874601	PTPRM	NA	2	58864898	ENSDARG00000089172	protein tyrosine phosphatase - receptor type - M	Signaling
DMR3:247801	BX004816.2	NA	3	254567	ENSDARG00000069019	Uncharacterized protein	NA
DMR3:364001	dkey-30g5.2	NA	3	357563	ENSDARG00000094838	dkey-30g5.2	NA
DMR3:398501	BX004816.4	100007517	3	402206	ENSDARG00000087012	Uncharacterized protein	NA
DMR3:2308001	ch211-254c8.1	NA	3	2309078	ENSDARG00000092176	ch211-254c8.1	NA
DMR3:2308001	dkey-73p2.2	NA	3	2282163	ENSDARG00000087429	dkey-73p2.2	NA
DMR3:3324801	CR388047.1	NA	3	3324452	ENSDARG00000101705	NA	NA
DMR3:4201201	dkey-36h5.1	NA	3	4189421	ENSDARG00000044235	dkey-36h5.1	NA
DMR3:4742901	slc25a38a	767662	3	4733901	ENSDARG00000059805	solute carrier family 25 - member 38a	NA
DMR3:5784601	5S_rRNA	NA	3	5787688	ENSDARG00000085478	5S ribosomal RNA	Translation
DMR3:5802301	TGFBR3L	100535410	3	5801330	ENSDARG00000088795	transforming growth factor - beta receptor III-like	Receptor
DMR3:5878601	pkn1b	100007897	3	5868579	ENSDARG00000062748	protein kinase N1b	Signaling
DMR3:5887101	pkn1b	100007897	3	5868579	ENSDARG00000062748	protein kinase N1b	Signaling
DMR3:5897901	pkn1b	100007897	3	5868579	ENSDARG00000062748	protein kinase N1b	Signaling
DMR3:6323901	ch73-144l3.2	NA	3	6317624	ENSDARG00000104099	ch73-144l3.2	NA
DMR3:6469201	ch211-12p12.4	NA	3	6439777	ENSDARG00000103806	ch211-12p12.4	NA
DMR3:6877601	mast1b	796144	3	6835018	ENSDARG00000088789	microtubule associated serine/threonine kinase 1b	Signaling
DMR3:7581301	174234	798270	3	7577248	ENSDARG00000102401	174234	NA
DMR3:7581301	174234	100538208	3	7577248	ENSDARG00000102401	174234	NA
DMR3:7581301	158517	NA	3	7492049	ENSDARG00000104611	158517	NA
DMR3:7581301	113443	777726	3	7417227	ENSDARG00000100803	113443	NA
DMR3:7925701	hook2	394086	3	7877216	ENSDARG00000099235	hook microtubule-tethering protein 2	Cytoskeleton
DMR3:7959301	ubn2b	561642	3	7954040	ENSDARG00000100508	ubinuclein 2b	NA
DMR3:8006101	ubn2b	561642	3	7954040	ENSDARG00000100508	ubinuclein 2b	NA
DMR3:8026501	trim35-25	561511	3	8034371	ENSDARG00000101055	tripartite motif containing 35-25	NA
DMR3:8026501	trim35-21	561579	3	8018550	ENSDARG00000102303	tripartite motif containing 35-21	NA
DMR3:8048501	trim35-25	561511	3	8034371	ENSDARG00000101055	tripartite motif containing 35-25	NA
DMR3:8063401	trim35-23	561453	3	8066950	ENSDARG00000102368	tripartite motif containing 35-23	NA
DMR3:8076201	trim35-22	561381	3	8078766	ENSDARG00000102264	tripartite motif containing 35-22	NA
DMR3:8086001	trim35-22	561381	3	8078766	ENSDARG00000102264	tripartite motif containing 35-22	NA
DMR3:8100101	TRIM35 (28 of 38)	561314	3	8101271	ENSDARG00000100721	tripartite motif containing 35	NA
DMR3:8104701	TRIM35 (28 of 38)	561314	3	8101271	ENSDARG00000100721	tripartite motif containing 35	NA
DMR3:8115901	TRIM35 (26 of 38)	NA	3	8120937	ENSDARG00000100373	tripartite motif containing 35	NA
DMR3:8126701	TRIM35 (26 of 38)	NA	3	8120937	ENSDARG00000100373	tripartite motif containing 35	NA
DMR3:8271901	TRIM35 (9 of 38)	541547	3	8254194	ENSDARG00000039108	tripartite motif containing 35	NA
DMR3:8287501	TRIM35 (9 of 38)	541547	3	8254194	ENSDARG00000039108	tripartite motif containing 35	NA
DMR3:8287501	CABZ01019905.1	NA	3	8294404	ENSDARG00000100364		NA
DMR3:8418501	ch73-322f21.2	NA	3	8420015	ENSDARG00000097075	ch73-322f21.2	NA
DMR3:8418501	trim35-10	799678	3	8412853	ENSDARG00000033071	tripartite motif containing 35-10	NA
DMR3:12500901	abca3b	564344	3	12440613	ENSDARG00000100524	ATP-binding cassette - sub-family A (ABC1) - member 3b	Receptor
DMR3:14007701	ch211-108d22.2	793004	3	14007244	ENSDARG00000097615	ch211-108d22.2	NA
DMR3:14011501	ch211-108d22.2	793004	3	14007244	ENSDARG00000097615	ch211-108d22.2	NA
DMR3:14015501	ch211-108d22.2	793004	3	14007244	ENSDARG00000097615	ch211-108d22.2	NA

DMR3:14081101	5S_rRNA	NA	3	14094820	ENSDARG00000091318	5S ribosomal RNA	Translation
DMR3:14175401	lppr2a	393428	3	14151274	ENSDARG00000101478	lipid phosphate phosphatase-related protein type 2a	Signaling
DMR3:14217601	lppr2a	393428	3	14151274	ENSDARG00000101478	lipid phosphate phosphatase-related protein type 2a	Signaling
DMR3:14257601	tmem56b	449652	3	14238210	ENSDARG00000098224	transmembrane protein 56b	NA
DMR3:21315301	ch73-54a8.2	NA	3	21248509	ENSDARG00000097315	ch73-54a8.2	NA
DMR3:28391001	12-Sep	564600	3	28371577	ENSDARG00000019191	septin 12	Cytoskeleton
DMR3:28587101	gsg1l	664746	3	28587975	ENSDARG00000037390	gsg1-like	NA
DMR3:28587101	dkey-20j1.2	NA	3	28519125	ENSDARG00000096409	dkey-20j1.2	NA
DMR3:30394101	syt3	100000615	3	30384523	ENSDARG00000075830	synaptotagmin III	Protein Binding
DMR3:30941901	tceb2	192341	3	30938434	ENSDARG00000037980	transcription elongation factor B (SIII) - polypeptide 2	Transcription
DMR3:30941901	TCEB2 (2 of 2)	NA	3	30935945	ENSDARG00000097471	transcription elongation factor B (SIII) - polypeptide 2 (18kDa - elongin B)	Transcription
DMR3:31850101	kcnc3a	559096	3	31821431	ENSDARG00000055855	potassium voltage-gated channel - Shaw-related subfamily - member 3a	Transport
DMR3:59892001	xylt2	563446	3	59886578	ENSDARG00000059557	xylosyltransferase II	Metabolism
DMR4:3494601	grm8a	792371	4	3464722	ENSDARG00000077654	glutamate receptor - metabotropic 8a	Receptor
DMR4:4873301	ptprz1b	553019	4	4825515	ENSDARG00000020871	protein tyrosine phosphatase - receptor-type - Z polypeptide 1b	Signaling
DMR4:29125001	fb11h05	563440	4	28860207	ENSDARG00000074009	fb11h05	NA
DMR4:29687601	ch211-214c20.1	NA	4	29666316	ENSDARG00000088082	ch211-214c20.1	NA
DMR4:29687601	BX248122.1	NA	4	29696528	ENSDARG00000100661		NA
DMR4:30277801	RNH1 (6 of 55)	560084	4	30254666	ENSDARG00000089582	ribonuclease/angiogenin inhibitor 1	Translation
DMR4:39324201	RNH1 (45 of 55)	571990	4	39287490	ENSDARG00000103755	ribonuclease/angiogenin inhibitor 1	Translation
DMR4:44703501	dkey-256i11.2	557877	4	44666428	ENSDARG00000093713	dkey-256i11.2	NA
DMR4:44703501	ch211-162i8.7	NA	4	44507344	ENSDARG00000096216	ch211-162i8.7	NA
DMR4:51339201	dkey-250k10.1	NA	4	51338256	ENSDARG00000102595	dkey-250k10.1	NA
DMR4:51348201	dkey-250k10.1	NA	4	51338256	ENSDARG00000102595	dkey-250k10.1	NA
DMR4:52940101	dkey-56m15.9	NA	4	52931823	ENSDARG00000103281	dkey-56m15.9	NA
DMR4:52956001	dkey-56m15.9	NA	4	52931823	ENSDARG00000103281	dkey-56m15.9	NA
DMR4:67286701	ch211-209j12.4	100333986	4	67142315	ENSDARG00000099403	ch211-209j12.4	NA
DMR4:67286701	ch211-209j12.1	NA	4	67271628	ENSDARG00000101634	ch211-209j12.1	NA
DMR4:67286701		NA	4	67272096	ENSDARG00000101594		NA
DMR4:69395401	ch211-76m11.3	NA	4	69292827	ENSDARG00000104890	ch211-76m11.3	NA
DMR4:69395401	ch211-76m11.5	NA	4	69278330	ENSDARG00000100006	ch211-76m11.5	NA
DMR4:69395401	ch211-76m11.11	NA	4	69106827	ENSDARG00000101828	ch211-76m11.11	NA
DMR4:70713201	ftr64	562311	4	70704404	ENSDARG00000101514	finTRIM family - member 64	NA
DMR4:70945701	CABZ01054394.4	101885937	4	70942037	ENSDARG00000098324		NA
DMR4:70945701	CABZ01054394.2	103910994	4	70951709	ENSDARG00000089715	Uncharacterized protein	NA
DMR4:72074701	BX855614.4	100333583	4	72069720	ENSDARG00000103751	NA	NA
DMR4:72107201	171551	798325	4	72087887	ENSDARG00000073708	171551	NA
DMR4:72819201	ptprb	100149130	4	72814552	ENSDARG00000076624	protein tyrosine phosphatase - receptor type - b	Signaling
DMR4:72841601	ptprb	100149130	4	72814552	ENSDARG00000076624	protein tyrosine phosphatase - receptor type - b	Signaling
DMR4:72895901	ptprb	100149130	4	72814552	ENSDARG00000076624	protein tyrosine phosphatase - receptor type - b	Signaling
DMR4:74120701	dkey-261j11.2	NA	4	74084251	ENSDARG00000101551	dkey-261j11.2	NA
DMR4:74120701	174944	103911006	4	74050132	ENSDARG00000099327	174944	NA
DMR4:74120701	RNH1 (53 of 55)	NA	4	74109908	ENSDARG00000104956	ribonuclease/angiogenin inhibitor 1	Translation
DMR4:74129901	dkey-261j11.2	NA	4	74084251	ENSDARG00000101551	dkey-261j11.2	NA
DMR4:74129901	174944	103911006	4	74050132	ENSDARG00000099327	174944	NA
DMR4:74143101	dkey-261j11.2	NA	4	74084251	ENSDARG00000101551	dkey-261j11.2	NA
DMR4:74143101	174944	103911006	4	74050132	ENSDARG00000099327	174944	NA
DMR4:75006601	ms4a17a.11	550363	4	74998757	ENSDARG00000053561	membrane-spanning 4-domains - subfamily A - member 17A.11	NA
DMR4:75342001	NA	NA	4	75309080	ENSDARG00000089296	NA	NA
DMR4:75579501	CU467646.4	NA	4	75565209	ENSDARG00000099945		NA
DMR4:75579501	dkey-240n22.7	NA	4	75470342	ENSDARG00000092241	dkey-240n22.7	NA
DMR5:3638301	MYO19	562279	5	3619704	ENSDARG00000073761	myosin XIX	Mitochondria
DMR5:3967401	prdx4	570477	5	3965601	ENSDARG00000069013	peroxiredoxin 4	Electron Transport
DMR5:4188901	PLA2G4C (3 of 4)	100332119	5	4172490	ENSDARG00000045982	phospholipase A2 - group IVC (cytosolic - calcium-independent)	Metabolism
DMR5:4250601	FO834898.1	101882086	5	4246450	ENSDARG00000102152	NA	NA
DMR5:4281001	FO834898.1	101882086	5	4246450	ENSDARG00000102152	NA	NA
DMR5:4291501	FO834898.1	101882086	5	4246450	ENSDARG00000102152	NA	NA
DMR5:4394001	angptl2a	569092	5	4372659	ENSDARG00000024030	angiopoietin-like 2a	Growth Factor
DMR5:5272901	TNC (2 of 2)	100149028	5	5215683	ENSDARG00000078362	tenascin C	ECM
DMR5:5986201	tnks1bp1	497601	5	5953586	ENSDARG00000068760	tankyrase 1 binding protein 1	Metabolism
DMR5:6976301	UNC5C (2 of 2)	NA	5	6902191	ENSDARG00000099133	unc-5 homolog C (C. elegans)	Development
DMR5:7035501	bmpr1ba	30742	5	6996909	ENSDARG00000104100	bone morphogenetic protein receptor - type Iba	Receptor
DMR5:7069901	ANGPTL2 (3 of 3)	100536883	5	7071316	ENSDARG00000098596	angiopoietin-like 2	Growth Factor
DMR5:7069901	bmpr1ba	30742	5	6996909	ENSDARG00000104100	bone morphogenetic protein receptor - type Iba	Receptor
DMR5:7178001	bmpr1ba	30742	5	6996909	ENSDARG00000104100	bone morphogenetic protein receptor - type Iba	Receptor
DMR5:8967401	gak	100151158	5	8936560	ENSDARG00000090654	cyclin G associated kinase	Signaling
DMR5:8971701	gak	100151158	5	8936560	ENSDARG00000090654	cyclin G associated kinase	Signaling
DMR5:8995601	gak	100151158	5	8936560	ENSDARG00000090654	cyclin G associated kinase	Signaling
DMR5:13216101	mxd1	570082	5	13206742	ENSDARG00000032039	MAX dimerization protein 1	Transcription
DMR5:13667801	npffr1l2	570189	5	13665350	ENSDARG00000062631	neuropeptide FF receptor 1 like 2	Receptor
DMR5:19369301	tchp	678595	5	19363994	ENSDARG00000035605	trichoplein - keratin filament binding	Cytoskeleton
DMR5:19830401	coro1ca	317741	5	19815719	ENSDARG00000035598	coronin - actin binding protein - 1Ca	Cytoskeleton
DMR5:59962201	tmem132e	564044	5	59845417	ENSDARG00000090830	transmembrane protein 132E	NA
DMR5:60584201	doc2b	NA	5	60249798	ENSDARG00000088293	double C2-like domains - beta	Metabolism
DMR5:60588801	doc2b	NA	5	60249798	ENSDARG00000088293	double C2-like domains - beta	Metabolism
DMR5:64357401	pmpca	492801	5	64344144	ENSDARG00000102607	peptidase (mitochondrial processing) alpha	Protease
DMR5:64364101	pmpca	492801	5	64344144	ENSDARG00000102607	peptidase (mitochondrial processing) alpha	Protease
DMR5:66964801	zbtb20	568779	5	66854811	ENSDARG00000005586	zinc finger and BTB domain containing 20	Transcription
DMR5:67041601	arhgap31	101885883	5	66992838	ENSDARG00000059472	Rho GTPase activating protein 31	Signaling
DMR5:67061901	b4galt4	492760	5	67057417	ENSDARG00000104042	UDP-Gal:betaGlcNAc beta 1 -4- galactosyltransferase - polypeptide 4	Metabolism
DMR5:67301801	gtf3aa	445389	5	67302476	ENSDARG00000030267	general transcription factor IIIAa	Transcription

DMR5:67401401	slc25a11	415189	5	67397951	ENSDARG00000035741	solute carrier family 25 (mitochondrial carrier; oxoglutarate carrier) - member 11	Mitochondria
DMR5:67408901	slc25a11	415189	5	67397951	ENSDARG00000035741	solute carrier family 25 (mitochondrial carrier; oxoglutarate carrier) - member 11	Mitochondria
DMR5:69209001	ch211-154e10.1	NA	5	69208901	ENSDARG00000094605	ch211-154e10.1	NA
DMR5:70970501	gpsm1b	NA	5	70956674	ENSDARG00000054874	G-protein signaling modulator 1b	Signaling
DMR5:71172001	nup214	NA	5	71099967	ENSDARG00000016212	nucleoporin 214	Nuclear Matrix
DMR5:71201101	fam78ab	100002878	5	71198499	ENSDARG00000088222	family with sequence similarity 78 - member Ab	NA
DMR5:71456001	ddx54	286777	5	71409979	ENSDARG00000105286	DEAD (Asp-Glu-Ala-Asp) box polypeptide 54	Transcription
DMR5:71582301	ddx54	286777	5	71409979	ENSDARG00000105286	DEAD (Asp-Glu-Ala-Asp) box polypeptide 54	Transcription
DMR6:36138801	ch211-205j18.1	NA	6	36138810	ENSDARG00000097518	ch211-205j18.1	NA
DMR6:37840601	herc2	NA	6	37774880	ENSDARG00000073841	HECT and RLD domain containing E3 ubiquitin protein ligase 2	Metabolism
DMR6:41535401	hemk1	560620	6	41512599	ENSDARG00000078894	HemK methyltransferase family member 1	Metabolism
DMR6:48370601	mov10a	566858	6	48349656	ENSDARG00000015829	Mov10 RISC complex RNA helicase a	NA
DMR6:59227701	fam210b	619270	6	58922776	ENSDARG00000052638	family with sequence similarity 210 - member B	NA
DMR6:59227701	shmt2	100144628	6	59204233	ENSDARG00000104414	serine hydroxymethyltransferase 2 (mitochondrial)	Metabolism
DMR6:59314501	fam210b	619270	6	58922776	ENSDARG00000052638	family with sequence similarity 210 - member B	NA
DMR6:59329201	fam210b	619270	6	58922776	ENSDARG00000052638	family with sequence similarity 210 - member B	NA
DMR6:59339901	fam210b	619270	6	58922776	ENSDARG00000052638	family with sequence similarity 210 - member B	NA
DMR6:59349401	fam210b	619270	6	58922776	ENSDARG00000052638	family with sequence similarity 210 - member B	NA
DMR6:59740801	NUFIP1	NA	6	59739185	ENSDARG00000101024	nuclear fragile X mental retardation protein interacting protein 1	NA
DMR6:59951801	pmepa1	436853	6	59814875	ENSDARG00000039325	prostate transmembrane protein - androgen induced 1	NA
DMR6:59951801	kdm6al	569207	6	59922011	ENSDARG00000059794	lysine (K)-specific demethylase 6A - like	NA
DMR7:3622201	ch211-282j17.11	557100	7	3619727	ENSDARG00000042752	ch211-282j17.11	NA
DMR7:3622201	ch211-282j17.10	557030	7	3608933	ENSDARG00000070850	ch211-282j17.10	NA
DMR7:47553201	ch211-186j3.6	556807	7	47143275	ENSDARG00000078088	ch211-186j3.6	NA
DMR7:53854501	csnk1g1	494092	7	53831696	ENSDARG00000104342	casein kinase 1 - gamma 1	Signaling
DMR7:53972301	pacsin3	393964	7	53952504	ENSDARG00000099339	protein kinase C and casein kinase substrate in neurons 3	Signaling
DMR7:54351401	fgf4	570435	7	54347408	ENSDARG00000105230	fibroblast growth factor 4	Growth Factor
DMR7:54351401	ch211-96b20.2	NA	7	54339827	ENSDARG00000098634	ch211-96b20.2	NA
DMR7:56172201	sult5a1	767718	7	56128616	ENSDARG00000007769	sulfotransferase family 5A - member 1	Metabolism
DMR7:56172201	ist1	321460	7	56171157	ENSDARG00000051888	increased sodium tolerance 1 homolog (yeast)	NA
DMR7:59082601	SEMA4F	NA	7	59032057	ENSDARG00000100308	sema domain - immunoglobulin domain (Ig) - transmembrane domain (TM) and short cytoplasmic domain	Signaling
DMR7:59099201	SEMA4F	NA	7	59032057	ENSDARG00000100308	sema domain - immunoglobulin domain (Ig) - transmembrane domain (TM) and short cytoplasmic domain	Signaling
DMR7:60200401	pcxb	58068	7	60151878	ENSDARG00000051939	pyruvate carboxylase b	Metabolism
DMR7:60330301	pcxb	58068	7	60151878	ENSDARG00000051939	pyruvate carboxylase b	Metabolism
DMR7:64599101	MMP15 (1 of 2)	100332426	7	64569906	ENSDARG00000013072	matrix metallopeptidase 15 (membrane-inserted)	Protease
DMR8:30671801	adora2aa	561701	8	30654845	ENSDARG00000033706	adenosine A2a receptor a	Receptor
DMR8:30711401	upb1	322660	8	30690197	ENSDARG00000011521	ureidopropionase - beta	Metabolism
DMR8:44636501	grk5l	567452	8	44629634	ENSDARG00000078815	G protein-coupled receptor kinase 5 like	Signaling
DMR8:46092501	mtor	324254	8	46069730	ENSDARG00000053196	mechanistic target of rapamycin (serine/threonine kinase)	Signaling
DMR8:46244801	mtor	324254	8	46069730	ENSDARG00000053196	mechanistic target of rapamycin (serine/threonine kinase)	Signaling
DMR8:46527701	ch211-196g2.7	NA	8	46529014	ENSDARG00000088067	ch211-196g2.7	NA
DMR8:46527701	sult1st3	368270	8	46505998	ENSDARG00000018361	sulfotransferase family 1 - cytosolic sulfotransferase 3	Metabolism
DMR8:47333201	pex10	449821	8	47329338	ENSDARG00000041511	peroxisomal biogenesis factor 10	Metabolism
DMR8:48309801	PRDM16	NA	8	48106771	ENSDARG00000078324	PR domain containing 16	NA
DMR8:48794601	ch211-278p7.5	NA	8	48790192	ENSDARG00000090171	ch211-278p7.5	NA
DMR8:48902001	tp73	368221	8	48859431	ENSDARG00000017953	tumor protein p73	Transcription
DMR8:48999401	aak1a	568999	8	48976998	ENSDARG00000011855	AP2 associated kinase 1a	Signaling
DMR8:49121501	GOLM1	393249	8	49103270	ENSDARG00000025858	golgi membrane protein 1	Golgi
DMR8:53196501	cacna1db	NA	8	53026462	ENSDARG00000101589	calcium channel - voltage-dependent - L type - alpha 1D subunit - b	Signaling
DMR9:4770701	fmnl2a	100006456	9	4764057	ENSDARG00000012586	formin-like 2a	Cytoskeleton
DMR9:19846401	pdxka	791775	9	19812494	ENSDARG00000088959	pyridoxal (pyridoxine - vitamin B6) kinase a	Metabolism
DMR9:19903201	ch211-141e20.5	NA	9	19904713	ENSDARG00000092425	ch211-141e20.5	NA
DMR9:19903201	ch211-141e20.2	NA	9	19870700	ENSDARG00000093349	ch211-141e20.2	NA
DMR9:19936801	ch211-141e20.6	NA	9	19939482	ENSDARG00000091958	ch211-141e20.6	NA
DMR9:19936801	ch211-141e20.2	NA	9	19870700	ENSDARG00000093349	ch211-141e20.2	NA
DMR9:23333701	lypd6b	554606	9	23329427	ENSDARG00000077643	LY6/PLAUR domain containing 6B	NA
DMR9:23490701	tmem163a	564964	9	23444374	ENSDARG00000079858	transmembrane protein 163a	NA
DMR9:24526901	tmeff2a	100004613	9	24438315	ENSDARG00000051824	transmembrane protein with EGF-like and two follistatin-like domains 2a	NA
DMR9:24736901	5S_rRNA	NA	9	24737010	ENSDARG00000080950	5S ribosomal RNA	Translation
DMR9:26576901	dkey-111i23.1	100537093	9	26388755	ENSDARG00000094811	dkey-111i23.1	NA
DMR9:43671001	znf385b	368405	9	43437937	ENSDARG00000006065	zinc finger protein 385B	Transcription
DMR9:50157501	ttc21b	569823	9	50111092	ENSDARG00000012368	tetratricopeptide repeat domain 21B	NA
DMR9:50379601	COBLL1 (2 of 2)	100332245	9	50347321	ENSDARG00000102829	cordon-bleu WH2 repeat protein-like 1	NA
DMR9:51915301	NA	NA	9	51916345	ENSDARG00000103688	NA	NA
DMR9:52799301	nme8	103911697	9	52796547	ENSDARG00000102567	NA	NA
DMR9:52892901	nme8	100037319	9	52872158	ENSDARG00000098944	NME/NM23 family member 8	NA
DMR9:52892901	smarcal1	560412	9	52812379	ENSDARG00000102265	SWI/SNF related - matrix associated - actin dependent regulator of chromatin - subfamily a-like 1	Epigenetic
DMR9:54539001	frmpd4	403129	9	54510852	ENSDARG00000075685	FERM and PDZ domain containing 4	Signaling
DMR9:55347801	glra2	NA	9	55334456	ENSDARG00000075012	glycine receptor - alpha 2	Receptor
DMR9:55370401	fancb	NA	9	55367242	ENSDARG00000067596	Fanconi anemia - complementation group B	NA
DMR9:56011201	edar	796370	9	55999636	ENSDARG00000099088	ectodysplasin A receptor	Receptor
DMR10:1817601	apc	386762	10	1822160	ENSDARG00000058868	adenomatous polyposis coli	Cytoskeleton
DMR10:3196401	pi4kaa	556956	10	3185947	ENSDARG00000076724	phosphatidylinositol 4-kinase - catalytic - alpha a	Signaling
DMR10:3220601	pi4kaa	556956	10	3185947	ENSDARG00000076724	phosphatidylinositol 4-kinase - catalytic - alpha a	Signaling
DMR10:3224801	pi4kaa	556956	10	3185947	ENSDARG00000076724	phosphatidylinositol 4-kinase - catalytic - alpha a	Signaling
DMR10:3286701	slc25a1b	795332	10	3269207	ENSDARG00000076381	slc25a1 solute carrier family 25 (mitochondrial carrier; citrate transporter) - member 1b	NA

DMR10:7154301	psd3l	NA	10	7038821	ENSDARG00000104820	pleckstrin and Sec7 domain containing 3 - like	Signaling
DMR10:7963401	osbp2	794414	10	7958020	ENSDARG00000053487	oxysterol binding protein 2	Receptor
DMR10:8034801	atp6v0a2a	561469	10	8030046	ENSDARG00000035538	ATPase - H+ transporting - lysosomal V0 subunit a2a	Metabolism
DMR10:8160001	pstpip2	100537857	10	8142980	ENSDARG00000089569	proline-serine-threonine phosphatase interacting protein 2	Signaling
DMR10:8217601	DHX29	553505	10	8196016	ENSDARG00000040326	DEAH (Asp-Glu-Ala-His) box polypeptide 29	NA
DMR10:10460601	sardh	394103	10	10429090	ENSDARG00000058102	sarcosine dehydrogenase	Metabolism
DMR10:10471301	sardh	394103	10	10429090	ENSDARG00000058102	sarcosine dehydrogenase	Metabolism
DMR10:15026401	dkey-88l16.3	NA	10	15024858	ENSDARG00000094055	dkey-88l16.3	NA
DMR10:15787501	BX629350.3	NA	10	15789429	ENSDARG00000099062	NA	NA
DMR10:17582401	dkey-76p7.7	NA	10	17576176	ENSDARG00000095267	dkey-76p7.7	NA
DMR10:17582401	slc2a11l	564849	10	17528511	ENSDARG00000062873	solute carrier family 2 (facilitated glucose transporter) - member 11-like	Transport
DMR10:21842801	pcdh1gb2	503573	10	21719677	ENSDARG00000101307	protocadherin 1 gamma b 2	ECM
DMR10:21842801	pcdh1g3	553987	10	21703066	ENSDARG00000099646	protocadherin 1 gamma 3	ECM
DMR10:21842801	pcdh1g18	554005	10	21780364	ENSDARG00000099447	protocadherin 1 gamma 18	ECM
DMR10:21842801	pcdh1gc5	503571	10	21832573	ENSDARG00000104826	protocadherin 1 gamma c 5	ECM
DMR10:21842801	pcdh1g9	NA	10	21744170	ENSDARG00000057519	protocadherin 1 gamma 9	ECM
DMR10:21842801	pcdh1gb9	553940	10	21761087	ENSDARG00000088475	protocadherin 1 gamma b 9	ECM
DMR10:21842801	pcdh1g11	NA	10	21751082	ENSDARG00000101665	protocadherin 1 gamma 11	ECM
DMR10:21842801	pcdh1gc6	503572	10	21836289	ENSDARG00000103950	protocadherin 1 gamma c 6	ECM
DMR10:21842801	162322	100003438	10	21825832	ENSDARG00000099035	162322	NA
DMR10:21842801	pcdh1g26	554012	10	21829275	ENSDARG00000099931	protocadherin 1 gamma 26	ECM
DMR10:21842801	pcdh1g1	553983	10	21693447	ENSDARG00000104007	protocadherin 1 gamma 1	ECM
DMR10:21842801	pcdh1g2	553986	10	21699273	ENSDARG00000101232	protocadherin 1 gamma 2	ECM
DMR10:21842801	pcdh1g30	554014	10	21843649	ENSDARG00000101865	protocadherin 1 gamma 30	ECM
DMR10:21842801	pcdh1g29	554013	10	21839895	ENSDARG00000104497	protocadherin 1 gamma 29	ECM
DMR10:21842801	pcdh1g22	323975	10	21819530	ENSDARG00000103013	protocadherin 1 gamma 22	ECM
DMR10:22833001	pcolcea	563867	10	22819881	ENSDARG00000056913	procollagen C-endopeptidase enhancer a	Protease
DMR10:25871301	trpc4a	102725537	10	25867549	ENSDARG00000070507	transient receptor potential cation channel - subfamily C - member 4a	Transport
DMR10:26248701	arfip2b	550342	10	26244066	ENSDARG00000056664	ADP-ribosylation factor interacting protein 2b	Translation
DMR10:27192001	cxadr	791793	10	27134330	ENSDARG00000043658	coxsackie virus and adenovirus receptor	Receptor
DMR10:32594301	mogat2	450063	10	32590949	ENSDARG00000019228	monoacylglycerol O-acyltransferase 2	Metabolism
DMR10:32607101	mogat2	450063	10	32590949	ENSDARG00000019228	monoacylglycerol O-acyltransferase 2	Metabolism
DMR10:32665001	mogat2	450063	10	32590949	ENSDARG00000019228	monoacylglycerol O-acyltransferase 2	Metabolism
DMR10:33225101	myl10	550569	10	33227953	ENSDARG00000062592	myosin - light chain 10 - regulatory	Cytoskeleton
DMR10:34645101	nbeaa	541373	10	34482439	ENSDARG00000070080	neurobeachin a	Development
DMR10:37363101	nf1b	564518	10	37324870	ENSDARG00000004184	neurofibromin 1b	Development
DMR10:38708701	mmp30	678531	10	38700055	ENSDARG00000045887	matrix metallopeptidase 30	Protease
DMR10:39732901	kirrel3a	571887	10	39555113	ENSDARG00000075806	kin of IRRE like 3 a	NA
DMR10:40062301	CLMP	NA	10	40031942	ENSDARG00000003145	CXADR-like membrane protein	Development
DMR10:41094801	antxr1b	556289	10	41052466	ENSDARG00000074075	anthrax toxin receptor 1b	Receptor
DMR11:1219201	atp2b2	557430	11	1122111	ENSDARG00000063433	ATPase - Ca++ transporting - plasma membrane 2	Signaling
DMR11:1321101	iars	334393	11	1311033	ENSDARG00000007955	isoleucyl-tRNA synthetase	Metabolism
DMR11:1486301	acot8	450052	11	1485983	ENSDARG00000014138	acyl-CoA thioesterase 8	Metabolism
DMR11:1631001	LRP1 (2 of 2)	NA	11	1630547	ENSDARG00000097827	low density lipoprotein receptor-related protein 1	Receptor
DMR11:1689401	LRP1 (2 of 2)	NA	11	1630547	ENSDARG00000097827	low density lipoprotein receptor-related protein 1	Receptor
DMR11:1703701	LRP1 (2 of 2)	NA	11	1630547	ENSDARG00000097827	low density lipoprotein receptor-related protein 1	Receptor
DMR11:1746801	LRP1 (2 of 2)	NA	11	1630547	ENSDARG00000097827	low density lipoprotein receptor-related protein 1	Receptor
DMR11:1765501	LRP1 (2 of 2)	NA	11	1630547	ENSDARG00000097827	low density lipoprotein receptor-related protein 1	Receptor
DMR11:38487401	epha8	570677	11	38449178	ENSDARG00000023609	eph receptor A8	Receptor
DMR12:1351201	pemt	393127	12	1287084	ENSDARG00000103614	phosphatidylethanolamine N-methyltransferase	Metabolism
DMR12:7709501	ank3b	100126016	12	7612388	ENSDARG00000077582	ankyrin 3b	Cytoskeleton
DMR12:34960201	112285	561476	12	34950573	ENSDARG00000053323	112285	NA
DMR13:2632001	wdr11	558865	13	2599773	ENSDARG00000075245	WD repeat domain 11	NA
DMR13:2665501	wdr11	558865	13	2599773	ENSDARG00000075245	WD repeat domain 11	NA
DMR13:10424801	ch73-54n14.2	NA	13	10362445	ENSDARG00000091900	ch73-54n14.2	NA
DMR13:10440101	ch73-54n14.2	NA	13	10362445	ENSDARG00000091900	ch73-54n14.2	NA
DMR13:12435901	enpep	504088	13	12411159	ENSDARG00000057064	glutamyl aminopeptidase	Protease
DMR13:12612301	metap1	503783	13	12474346	ENSDARG00000033440	methionyl aminopeptidase 1	Protease
DMR13:13109801	fgfr3	58129	13	13085956	ENSDARG00000004782	fibroblast growth factor receptor 3	Receptor
DMR13:18077601	tet1	101883702	13	18066195	ENSDARG00000075230	tet methylcytosine dioxygenase 1	Epigenetic
DMR13:22908101	supv3l1	570852	13	22907135	ENSDARG00000077728	suppressor of var1 - 3-like 1 (S. cerevisiae)	NA
DMR13:23021701	sorbs1	NA	13	23002216	ENSDARG00000103435	sorbin and SH3 domain containing 1	Cytoskeleton
DMR13:23363001	prim2	407653	13	23357832	ENSDARG00000052721	primase - DNA - polypeptide 2	Transcription
DMR13:24034601	galnt2	570248	13	23938127	ENSDARG00000003829	UDP-N-acetyl-alpha-D-galactosamine:polypeptide N-acetylgalactosaminyltransferase 2	Metabolism
DMR13:26639501	fancl	406255	13	26573447	ENSDARG00000007885	Fanconi anemia - complementation group L	Protease
DMR13:29142601	MYOF (2 of 2)	559066	13	29108284	ENSDARG00000017128	myoferlin	ECM
DMR14:4045801	dhrs13l1	402945	14	4044395	ENSDARG00000098746	dehydrogenase/reductase (SDR family) member 13 like 1	Metabolism
DMR14:4352901	GABRA4	NA	14	4339747	ENSDARG00000013389	gamma-aminobutyric acid (GABA) A receptor - alpha 4	Receptor
DMR14:6224301	ch211-198i6.4	NA	14	6222903	ENSDARG00000105183	ch211-198i6.4	NA
DMR14:6407001	COL23A1 (2 of 2)	NA	14	6322136	ENSDARG00000095339	collagen - type XXIII - alpha 1	Cytoskeleton
DMR14:8610101	153681	751719	14	8587515	ENSDARG00000086017	153681	NA
DMR14:8613701	153681	751719	14	8587515	ENSDARG00000086017	153681	NA
DMR14:47320801	crybb1l1	553473	14	47316817	ENSDARG00000007576	crystallin - beta B1 - like 1	Development
DMR15:14568101	numbl	497616	15	14500202	ENSDARG00000101949	numb homolog (Drosophila)-like	Signaling
DMR15:17327201	dhx40	570225	15	17322221	ENSDARG00000041586	DEAH (Asp-Glu-Ala-His) box polypeptide 40	Transcription
DMR15:18099401	phldb1b	570275	15	18012375	ENSDARG00000079378	pleckstrin homology-like domain - family B - member 1b	NA
DMR16:1359501	cers2b	565295	16	1338362	ENSDARG00000058992	ceramide synthase 2b	Metabolism
DMR16:37267901	TSNARE1	101882035	16	37212994	ENSDARG00000088650	t-SNARE domain containing 1	NA
DMR16:43445101	FO704821.1	100151586	16	43445282	ENSDARG00000104344	Uncharacterized protein	NA
DMR16:43445101	ccdc127a	751722	16	43443996	ENSDARG00000099190	coiled-coil domain containing 127a	NA
DMR16:44448301	sult2st3	777792	16	44324431	ENSDARG00000028367	sulfotransferase family 2 - cytosolic sulfotransferase 3	Metabolism

DMR16:54373001	xrcc1	445480	16	54351049	ENSDARG00000009494	X-ray repair complementing defective repair in Chinese hamster cells 1	DNA Repair
DMR16:55305301	CNDP1	619255	16	55296448	ENSDARG00000069583	carnosine dipeptidase 1 (metallopeptidase M20 family)	Protease
DMR17:656101	ch211-193k8.5	NA	17	643986	ENSDARG00000100894	ch211-193k8.5	NA
DMR17:656101	dnajc17	393216	17	626928	ENSDARG00000104959	DnaJ (Hsp40) homolog - subfamily C - member 17	Protein Binding
DMR17:730801	dnajc17	393216	17	626928	ENSDARG00000104959	DnaJ (Hsp40) homolog - subfamily C - member 17	Protein Binding
DMR17:1552001	NA	NA	17	1527965	ENSDARG00000014717	NA	NA
DMR17:4691101	KLHL29 (1 of 2)	100334314	17	4421283	ENSDARG00000043799	kelch-like family member 29	
DMR17:7873501	syne1b	NA	17	7687722	ENSDARG00000063068	spectrin repeat containing - nuclear envelope 1b	Epigenetic
DMR17:8203801	cdc42bpaa	NA	17	8166170	ENSDARG00000104283	CDC42 binding protein kinase alpha (DMPK-like) a	Signaling
DMR17:8778001	psmc1a	336786	17	8741836	ENSDARG00000030537	proteasome (prosome - macropain) 26S subunit - ATPase - 1a	Protease
DMR17:14505201	daam1a	557451	17	14459424	ENSDARG00000015059	dishevelled associated activator of morphogenesis 1a	Development
DMR17:14772501	ch211-266o15.1	560900	17	14723371	ENSDARG00000057681	ch211-266o15.1	NA
DMR17:51757501	numb	692064	17	51732871	ENSDARG00000027279	numb homolog (Drosophila)	Signaling
DMR17:52816001	meis2a	170454	17	52736268	ENSDARG00000098240	Meis homeobox 2a	Transcription
DMR17:52867901	C17H15orf41	NA	17	52854307	ENSDARG00000070461	chromosome 15 open reading frame 41	NA
DMR17:52913101	C17H15orf41	NA	17	52854307	ENSDARG00000070461	chromosome 15 open reading frame 41	NA
DMR18:2941701	clns1a	30699	18	2941658	ENSDARG00000103027	chloride channel - nucleotide-sensitive - 1A	Transport
DMR18:2971401	rsf1a	NA	18	2967706	ENSDARG00000102589	remodeling and spacing factor 1a	Binding Protein
DMR18:3276701	pak1	373103	18	3268382	ENSDARG00000103959	p21 protein (Cdc42/Rac)-activated kinase 1	Signaling
DMR18:3456301	eif2a	573991	18	3455556	ENSDARG00000101061	eukaryotic translation initiation factor 2A	Translation
DMR18:11004901	ch211-59c24.1	567512	18	10998743	ENSDARG00000042189	ch211-59c24.1	NA
DMR18:39019801	myo5aa	562188	18	38918910	ENSDARG00000061635	myosin Vaa	Cytoskeleton
DMR18:48920001	ppp1r37	562516	18	48869580	ENSDARG00000078458	protein phosphatase 1 - regulatory subunit 37	Signaling
DMR18:50799701	ddb1	NA	18	50803251	ENSDARG00000074431	damage-specific DNA binding protein 1	DNA Repair
DMR19:23601	gpd1c	406615	19	24933	ENSDARG00000036942	glycerol-3-phosphate dehydrogenase 1c	Metabolism
DMR19:35537801	macf1a	562190	19	35452782	ENSDARG00000028533	microtubule-actin crosslinking factor 1a	Cytoskeleton
DMR19:48419401	psmd3	393544	19	48388925	ENSDARG00000018124	proteasome (prosome - macropain) 26S subunit - non-ATPase - 3	Protease
DMR19:48419401	btr30	393394	19	48322065	ENSDARG00000102591	bloodthirsty-related gene family - member 30	NA
DMR19:48419401	btr30	103909280	19	48322065	ENSDARG00000102591	bloodthirsty-related gene family - member 30	NA
DMR20:3615101	CABZ01071723.1	NA	20	3418897	ENSDARG00000017795	Uncharacterized protein	NA
DMR20:4992601	arid1b	569589	20	4855142	ENSDARG00000092503	AT rich interactive domain 1B (SWI1-like)	Epigenetic
DMR20:5558801	nrxn3b	570698	20	5518645	ENSDARG00000062693	neurexin 3b	Receptor
DMR20:9384301	BEGAIN	563117	20	9367910	ENSDARG00000043673	brain-enriched guanylate kinase-associated	Signaling
DMR20:25707301	cyp2j20	797309	20	25698437	ENSDARG00000094057	cytochrome P450 - family 2 - subfamily J - polypeptide 20	Metabolism
DMR20:28546801	dpf3	562738	20	28531696	ENSDARG00000025309	D4 - zinc and double PHD fingers - family 3	Transcription
DMR20:30709801	ccr6a	558616	20	30707668	ENSDARG00000087474	chemokine (C-C motif) receptor 6a	Receptor
DMR20:32207101	grm1a	555576	20	32144506	ENSDARG00000026796	glutamate receptor - metabotropic 1a	Receptor
DMR20:33274101	ddx1	556790	20	33272289	ENSDARG00000032117	DEAD (Asp-Glu-Ala-Asp) box helicase 1	Translation
DMR21:1920301	WDR7	NA	21	1904090	ENSDARG00000100274	WD repeat domain 7	NA
DMR21:3157401	CTIF	100537309	21	3090902	ENSDARG00000090617	CBP80/20-dependent translation initiation factor	Translation
DMR21:20216801	dkey-247m21.3	559089	21	20193366	ENSDARG00000001906	dkey-247m21.3	NA
DMR21:27805601	nrxn2a	558326	21	27599593	ENSDARG00000061454	neurexin 2a	NA
DMR21:29267701	BX537120.1	559695	21	29258056	ENSDARG00000098587	NA	NA
DMR21:29368001	171310	100124620	21	29326179	ENSDARG00000099580	171310	NA
DMR21:29368001	BX537120.1	559695	21	29258056	ENSDARG00000098587		NA
DMR21:34921401	lipia	445105	21	34910543	ENSDARG00000007108	lipase - member Ia	Metabolism
DMR21:35219601	ubtd2	436991	21	35181320	ENSDARG00000069184	ubiquitin domain containing 2	Protease
DMR21:35403401	dkeyp-23e4.3	566257	21	35347886	ENSDARG00000074819	dkeyp-23e4.3	NA
DMR21:35457401	dkeyp-23e4.3	566257	21	35347886	ENSDARG00000074819	dkeyp-23e4.3	NA
DMR21:35759501	sgcd	324961	21	35617273	ENSDARG00000098573	sarcoglycan - delta (dystrophin-associated glycoprotein)	Cytoskeleton
DMR21:36469801	gabrb4	566514	21	36393096	ENSDARG00000099096	gamma-aminobutyric acid (GABA) A receptor - beta 4	Receptor
DMR21:44327201	gabra3	NA	21	44306279	ENSDARG00000090883	gamma-aminobutyric acid (GABA) A receptor - alpha 3	Receptor
DMR22:52901	mrpl20	751640	22	19300	ENSDARG00000090462	mitochondrial ribosomal protein L20	Translation
DMR22:4466901	ch73-256j6.7	NA	22	4435183	ENSDARG00000092336	ch73-256j6.7	NA
DMR22:4466901	ch73-256j6.5	NA	22	4426983	ENSDARG00000078519	ch73-256j6.5	NA
DMR22:4470201	ch73-256j6.7	NA	22	4435183	ENSDARG00000092336	ch73-256j6.7	NA
DMR22:4470201	ch73-256j6.5	NA	22	4426983	ENSDARG00000078519	ch73-256j6.5	NA
DMR22:9624901	RNH1 (12 of 55)	569265	22	9605488	ENSDARG00000093402	ribonuclease/angiogenin inhibitor 1	
DMR22:16239001	cdc14ab	565969	22	16165001	ENSDARG00000057016	cell division cycle 14Ab	Cell Cycle
DMR22:20722801	amh	493624	22	20722204	ENSDARG00000014357	anti-Mullerian hormone	Hormone
DMR22:26599401	capn8	337730	22	26380642	ENSDARG00000055715	calpain 8	Protease
DMR22:26599401	capn8	103909603	22	26380642	ENSDARG00000055715	calpain 8	Protease
DMR22:26599401	capn2l	445091	22	26423303	ENSDARG00000034211	calpain 2 - (m/II) large subunit - like	Protease
DMR22:26599401	capn2l	550505	22	26423303	ENSDARG00000034211	calpain 2 - (m/II) large subunit - like	Protease
DMR22:26625901	capn2l	445091	22	26423303	ENSDARG00000034211	calpain 2 - (m/II) large subunit - like	Protease
DMR22:26625901	capn2l	550505	22	26423303	ENSDARG00000034211	calpain 2 - (m/II) large subunit - like	Protease
DMR22:29724401	pdcd4b	321061	22	29709788	ENSDARG00000041022	programmed cell death 4b	Apoptosis
DMR22:30129001	add3a	556762	22	30098050	ENSDARG00000040874	adducin 3 (gamma) a	Cytoskeleton
DMR22:30136601	add3a	556762	22	30098050	ENSDARG00000040874	adducin 3 (gamma) a	Cytoskeleton
DMR22:34908801	slit1b	561685	22	34867222	ENSDARG00000099446	slit homolog 1b (Drosophila)	Development
DMR22:37451201	ch73-334e23.1	NA	22	37412522	ENSDARG00000095844	ch73-334e23.1	NA
DMR22:37456701	ch73-334e23.1	NA	22	37412522	ENSDARG00000095844	ch73-334e23.1	NA
DMR23:44152601	CORIN	100320926	23	44137901	ENSDARG00000101281	corin - serine peptidase	Protease
DMR23:45582101	PSIP1 (1 of 2)	402866	23	45487012	ENSDARG00000077405	PC4 and SFRS1 interacting protein 1	NA
DMR23:45582101	PSIP1 (1 of 2)	407619	23	45487012	ENSDARG00000077405	PC4 and SFRS1 interacting protein 1	NA
DMR24:834001	napga	541353	24	742695	ENSDARG00000006617	N-ethylmaleimide-sensitive factor attachment protein - gamma a	Metabolism
DMR24:24876301	nupl1	406510	24	24863033	ENSDARG00000033965	nucleoporin like 1	Nuclear Matrix
DMR24:27134701	dip2ca	563890	24	26944115	ENSDARG00000062154	DIP2 disco-interacting protein 2 homolog Ca (Drosophila)	Development
DMR25:6345401	snx33	100001421	25	6323636	ENSDARG00000014954	sorting nexin 33	Signaling
DMR25:35284401	cpne8	100332028	25	35269605	ENSDARG00000025189	copine VIII	NA
DMR25:36468801	wwox	NA	25	36389835	ENSDARG00000007614	WW domain containing oxidoreductase	Metabolism
DMR25:36680301	rfwd3	100329327	25	36679404	ENSDARG00000087752	ring finger and WD repeat domain 3	Signaling
